# Few‐layer Black Phosphorous Catalyzes Radical Additions to Alkenes Faster than Low‐valence Metals

**DOI:** 10.1002/cctc.201902276

**Published:** 2020-02-25

**Authors:** María Tejeda‐Serrano, Vicent Lloret, Bence G. Márkus, Ferenc Simon, Frank Hauke, Andreas Hirsch, Antonio Doménech‐Carbó, Gonzalo Abellán, Antonio Leyva‐Pérez

**Affiliations:** ^1^ Instituto de Tecnología Química Universitat Politècnica de València-Consejo Superior de Investigaciones Científicas Avda. de los Naranjos s/n 46022 Valencia Spain; ^2^ Friedrich-Alexander-Universität Erlangen-Nürnberg (FAU) Nikolaus-Fiebiger Straße 10 91058 Erlangen Germany; ^3^ Zentralinstitut für neue Materialien und Prozesstechnik (ZMP) Dr.-Mack Straße 81 90762 Fürth Germany; ^4^ Department of Physics Budapest University of Technology and Economics PO Box 91 H-1521 Budapest Hungary; ^5^ MTA-BME Lendület Spintronics Research Group (PROSPIN) POBox 91 H-1521 Budapest Hungary; ^6^ Departamento de Química Analítica Universitat de València Dr. Moliner 50 46100 Burjassot, València Spain; ^7^ Instituto de Ciencia Molecular (ICMol) Universidad de Valencia Catedrático José Beltrán 2 46980 Paterna, Valencia Spain

**Keywords:** black phosphorus, *p*-block catalysis, radical addition, alkenes, iron, 2D materials

## Abstract

The substitution of catalytic metals by *p*‐block main elements has a tremendous impact not only in the fundamentals but also in the economic and ecological fingerprint of organic reactions. Here we show that few‐layer black phosphorous (FL‐BP), a recently discovered and now readily available 2D material, catalyzes different radical additions to alkenes with an initial turnover frequency (TOF_0_) up to two orders of magnitude higher than representative state‐of‐the‐art metal complex catalysts at room temperature. The corresponding electron‐rich BP intercalation compound (BPIC) KP_6_ shows a nearly twice TOF_0_ increase with respect to FL‐BP. This increase in catalytic activity respect to the neutral counterpart also occurs in other 2D materials (graphene vs. KC_8_) and metal complex catalysts (Fe^0^ vs. Fe^2−^ carbon monoxide complexes). This reactive parallelism opens the door for cross‐fertilization between 2D materials and metal catalysts in organic synthesis.

## Graphical abstract

Phosphorene catalyzes with extraordinary activity radical additions to alkenes, faster than optimized metal catalysts, opening the door for future applications of 2D heteroatom materials in organic synthesis.

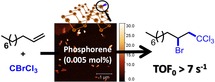

## Introduction

**Figure 1:**
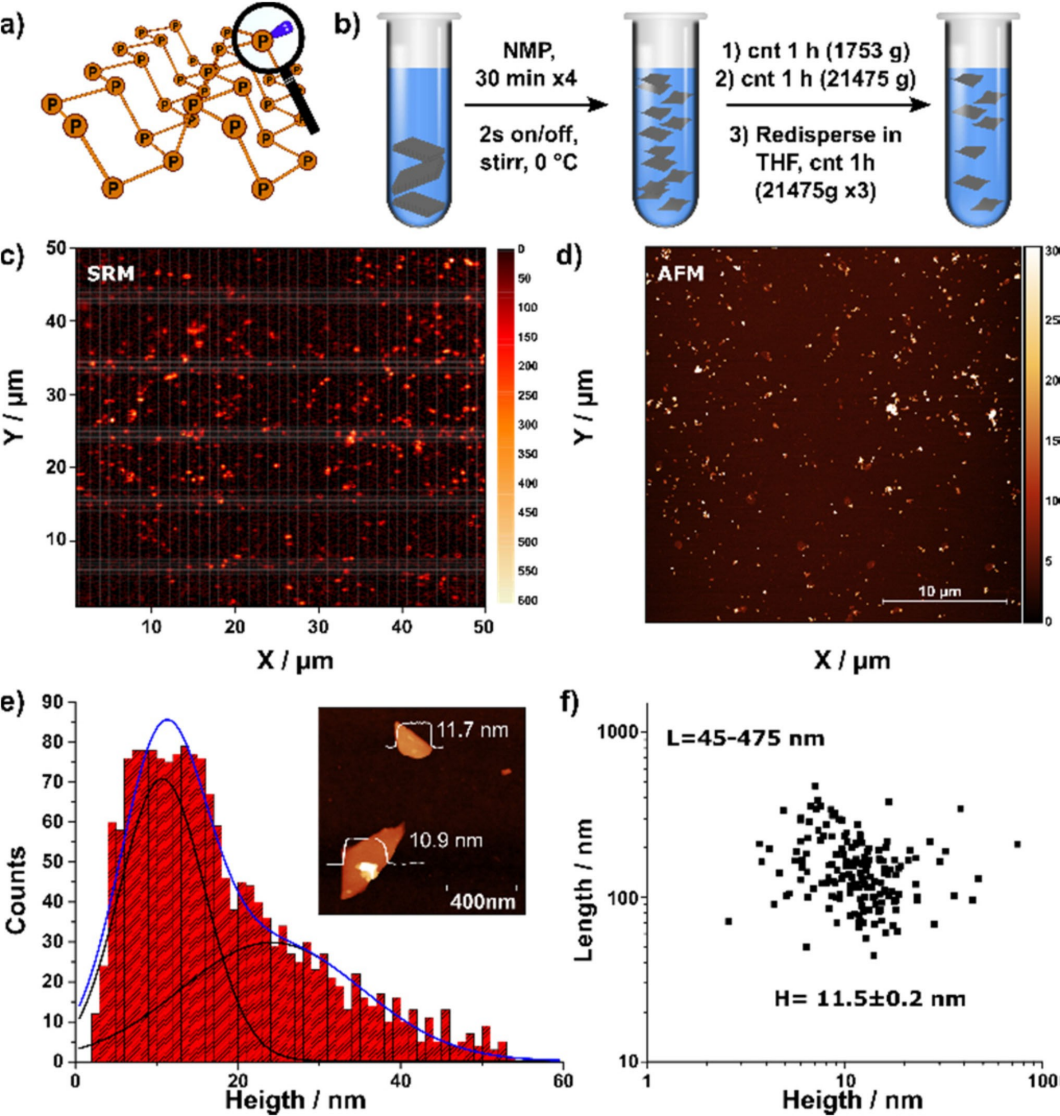
a) Schematic representation of the BP structure highlighting the presence of lone pair electrons. b) General scheme of the liquid phase exfoliation and sequential solvent exchange process from NMP to THF. c) Wide area Scanning Raman Microscopy image of FL‐BP deposited on Si−SiO_2_ substrates. d) corresponding AFM image. e) Histogram of the apparent thickness of the exfoliated FL‐BP obtained from AFM. The inset shows an AFM image of two nanosheets along with its corresponding height profile of ca. 11 and 12 nm, respectively. f) Plot of the nanosheet length as a function of the flake height considering a total amount of 170 replicates. The average thickness is H=11.5±0.2 nm and lateral sizes are ranging from 45 to 475 nm.

The search for *p*‐block main element compounds to substitute generally more toxic, expensive and less available metal catalysts, is a current topic of much interest.^1^ The replacement of late‐heavy by first‐row transition metals, based on isovalence, isoelectronics and isolobal orbital analogies, among others,^2^ has been continued with metal‐free soluble nitrogen‐, sulfur‐ and phosphorous‐containing molecules,^3^ including insoluble compounds such as graphene,^4^ fullerenes^5^ and carbon nitrides.^6^ However, most of the examples reported involve a two‐electron rather than a one‐electron redox process, since the latter is generally more difficult to handle for *p*‐block main elements beyond photoinduced processes.6b Looking back into the analogies between late‐heavy and first‐row transition metals, one finds that the ordered aggregation of metals in the form of clusters and nanoparticles enables encumbered electronic states, otherwise severely restricted in atomic systems, since the metal atoms cooperate to stabilize intermediate, atypical electronic states.^7^ This effect is particularly effective for low‐valence states and, for instance, reduced gold nanoparticles act as electron sinks for catalytic radical reactions that otherwise would not occur.^8^ Following this rationale, one might expect that redox‐active *p*‐block elements in low oxidation state will enable radical reactions after stabilization of electron‐rich intermediates by a suitable designed atomic network. In this sense,^9^ post‐graphene monoelemental two dimensional (2D) materials of Group 15, also called 2D‐pnictogens (P, As, Sb, and Bi), represent a promising alternative due to their large chemically active surface and their ability to adsorb and stabilize unsaturated organic molecules through van der Waals interactions.^10^ Specifically, black phosphorus (BP) consists of sp^3^ hybridized P atoms showing a puckered structure with a dative electron lone pair located on every surface atom (Figure 1a). The availability of these surface atom orbitals for external reactants together with the cooperativity of the atomic network and the possibility of breaking temporarily P−P bonds to exchange one electron,^11^ would make, in principle, this 2D material a potential catalyst for radical reactions. To the best of our knowledge, examples of neat P aggregates as catalysts in organic synthesis are very scarce,^12^ besides P alloyed metal nanoparticles.^13^

## Results and Discussion

### Synthesis of few‐layer black phosphorus (FL‐BP)

In order to study radical reactions catalyzed by BP nanosheets in conventional organic solvents, liquid phase exfoliation (LPE) of bulk BP into high‐quality few‐layers (FL‐BP) has been developed in a two‐step process. Firstly, dispersions of BP in NMP were obtained in an Ar‐filled glove‐box (<0.1 ppm O_2_ and <0.1 ppm H_2_O), avoiding oxidation and decomposition of the catalyst.^14^ Afterwards, the FL‐BP was transferred to THF by sequential ultracentrifugation and re‐dispersion, leading to stable dispersions with a P concentration of 0.001 wt% determined by ICP and with <0.01 wt% of NMP (See experimental in SI for a detailed procedure). Figure 1c shows an excellent wide‐area correlation between topographic atomic force microscopy (AFM) and scanning Raman microscopy (SRM) of the as‐prepared nanosheet dispersions spin‐coated on SiO_2_/Si wafers. The sample statistics show FL‐BP flakes 11.5 nm in thickness and 45–475 nm in lateral dimensions (Figure 1b and SI1). The A^1^_g_/A^2^_g_>0.4 intensity ratio statistics (average 0.79) indicate the absence of oxidation (see Figures S1 and S2 for additional characterization).12b, 15

### Radical addition of perhalomethanes to alkenes

Table 1 shows the catalytic results for a challenging metal‐catalyzed radical reaction at room temperature, *i. e*. the coupling between 1‐decene **1** and CBrCl_3_ **2**, which generally requires to proceed >0.1 mol% of metal catalyst without radical promoters, a high excess of **2** (generally employed as a solvent) and/or heating conditions (see Tables S1 and S2 for a complete set of catalysts and reaction conditions).^16^ In contrast to any previous metal catalyst, FL‐BP catalyzes the coupling with 0.005 mol% and a TOF_0_≈7.1 s^−1^ (entry 1), almost 3 orders of magnitude higher than the well‐known catalyst Fe(CO)_5_,^17^ where the TOF_0_ is 0.014 s^−1^ (entry 6). The reaction with FL‐BP catalyst can be run at ten‐time higher scale without significant depletion in the catalytic activity (see SI), to give a 50 % isolated yield of **3**. Other representative 2D and redox‐active nanoparticulated materials such as graphene, single‐walled carbon nanotubes (SWCNT), boron nitride, nanotitania, nanoceria and, remarkably, pristine, non‐exfoliated BP, do not catalyze the reaction (entries 3–8) at any catalyst loading tested. In contrast, the more electron‐rich black phosphorus intercalation compound (BPIC) material KP_6_,^18^ which basically consists of K atoms intercalated between the FL‐BP structure (similarly to alkali graphite intercalated compounds, GICs) and donating its odd electron to the 2D structure, gives the highest TOF_0_ of all compounds tested, although with a lower final yield of **3** (10 s^−1^, entry 9), and the GIC KC_8_ 19 also showed an excellent TOF_0_=0.76 s^−1^ and 52 % yield (entry 10). These results suggest that the more electron rich the 2D material, the higher the catalytic activity.

**Table 1:** Catalytic results for the radical coupling between **1** and **2**. The new formed bonds are highlighted in black. The catalyst mol % is the lowest to achieve maximum TOF_0_ after 24 h.

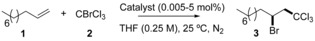			
Entry	Catalyst [mol %]	TOF_0_ [s^−1^]	Yield [3, %]
1	FL‐BP (0.005)	7.123	51
2	Fe(CO)_5_ (5)	0.014	60
3	Graphene (5)	–	0
4	SWCT (5)	–	0
5	Boron nitride (5)	–	0
6	Nanotitania (5)	–	0
7	Nanoceria (5)	–	0
8	BP (0.005–5)	–	0
9	KP_6_ (3)	10.036	6
10	KC_8_ (5)	0.765	52
11	Na_2_Fe_2_(CO)_8_ (5)	0.018	81
12	FeCl_3_ (5)	–	0
13	CuCl (5)	–	0
14	NiCl_2_ (5)	–	0
15	RuCl_2_(PPh_3_)_2_ (5)	–	0

To gain further insights into the by FL‐BP catalyzed radical coupling reaction, we followed the reaction between 1‐hexene **4** and CBrCl_3_ **2** *in‐situ* by liquid Raman spectroscopy, cyclic voltammetry, gas‐chromatography coupled to mass spectrometry (GC‐MS) and nuclear magnetic resonance (NMR), which allows the simultaneous identification and characterization of reagents, products and the FL‐BP catalyst during reaction.

**Figure 2:**
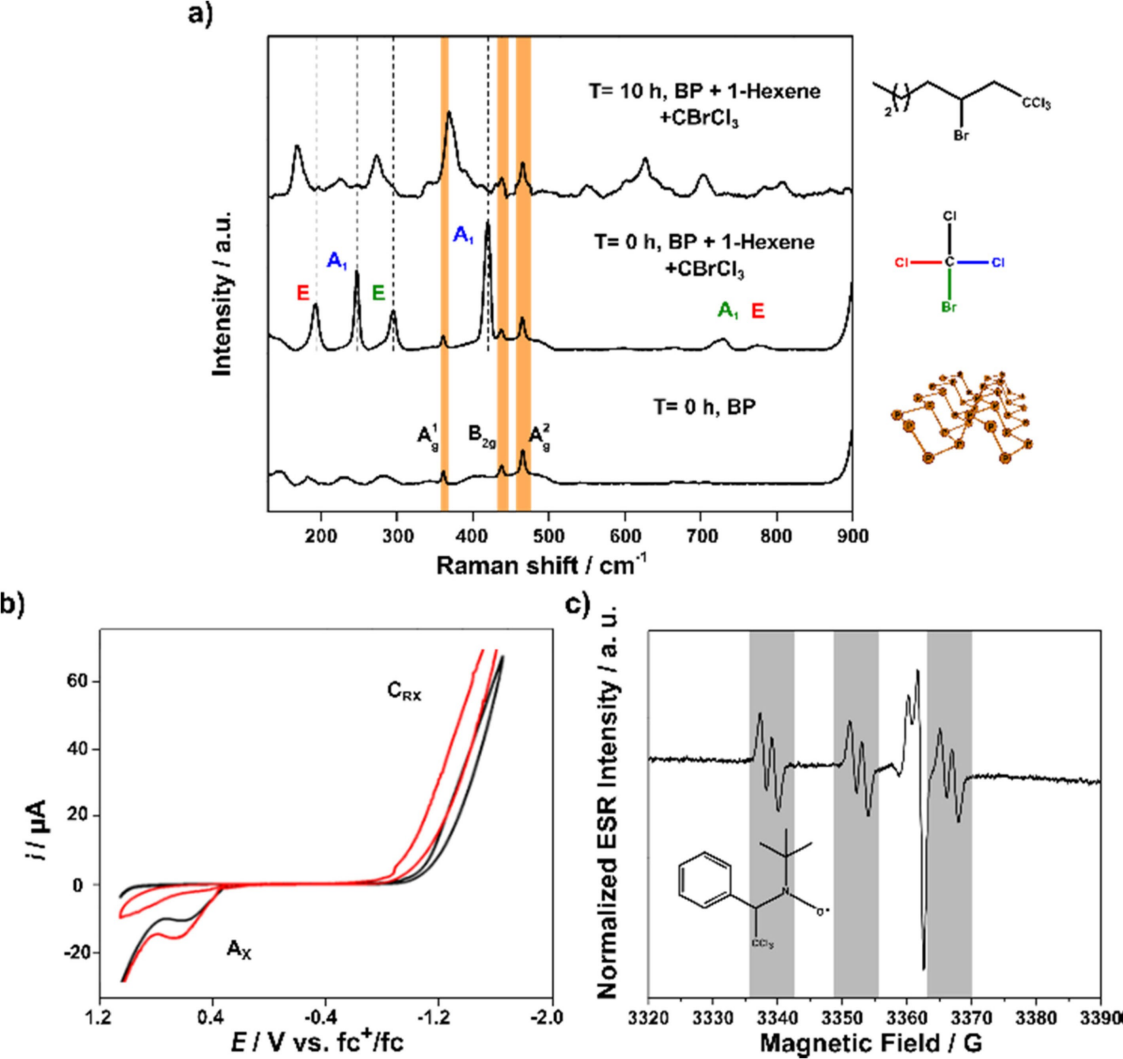
a) *In‐situ* liquid Raman spectra of BP in THF, after the addition of 1‐hexene **4** and CBrCl_3_ **2** (t=0 h), and after 10 h reaction time. The BP modes can be observed during the reaction and the corresponding bands of **2** disappear with time, giving rise to the new spectroscopic bands of the product. The different vibrational modes of C−Cl, Cl−C−Cl and C−Br are assigned with different colors. These results have been backed by GC‐MS and NMR analysis. b) Cyclic voltammograms at glassy carbon electrode of a 10 mM **1** plus 10 mM **2** solution in 0.10 M Bu_4_NPF_6_/DMSO before (red) and after (black) adding catalytic FL‐BP. Potential scan rate 50 mV s^−1^. c) Emergent PBN EPR signal (see also Figure S24d) due to the formation of PBN−CCl_3_ adduct in presence of FL‐BP in THF.

Figure 2 shows the Raman spectra of the FL‐BP dispersion in THF (t=0 h), in which the A^1^_g_, B_2g_ and A^2^_g_ BP vibrational bands can be seen at 362, 440 and 466 cm^−1^, respectively (see also Figures S3–5). In turn, the Raman spectra of **4**, presents a main characteristic band at 1641 cm^−1^ (Figure S5), which corresponds to the strong and polarized alkene C=C stretching vibration, whereas CBrCl_3_ presents six bands in total: a main vibrational mode at 423 cm^−1^, three bending modes at 191, 247 and 294 cm^−1^, and two stretching bands at 719 and 776 cm^−1^ (see control spectra of the pure compounds and THF in Figures S3 and S5).^20^ In presence of FL‐BP, the coupling between **4** and **2** leads to the product 3‐bromo‐1,1,1‐trichloroheptane **5** (t=10 h), in which the C−Br vibration decreases to give a characteristic band at 372 cm^−1^, and three Raman bands emerge at 168, 224 and 275 cm^−1^.

Figure 2 also shows the cyclic voltammograms recorded during the reaction between **1** and **2** with (black) and without (red) FL‐BP as a catalyst (**1** remains silent under the experimental conditions and the voltammogram of **2** can be seen in Figure S6). The intensity of both the reduction and the coupled oxidation signals of **2** (C_RX_ and A_x_, respectively) decreases in the presence of the FL‐BP catalyst, and this intensity further decreases with additional FL‐BP amounts (Figure S7). However, no apparent changes in the oxidation state of P occur, in contrast with the clear oxidation that suffers FL‐BP when the reactants are not present (Figure S8).^21^ The stability FL‐BP after the reaction was also evaluated by SRM by drop casting the dispersions in SiO_2_ wafers and washing with 2‐propanol and acetone (Figure S9), and the A^1^_g_/A^2^_g_ ratio exhibits a value higher than 0.4, thus corroborating that the structure remains un‐oxidized after the reaction. These electrochemical and spectroscopic data support that FL‐BP catalyzes the radical coupling between **1** (or **4**) and **2**, and that the P atoms do not change its oxidation state during reaction.

The fact that pristine BP is inactive as a catalyst (entry 8 in Table 1) suggests that a previously well‐delaminated BP material is essential for the catalysis. Indeed, four new samples of FL‐BP with different concentrations were prepared by dilution of a mother dispersion (Figure S10) and the radical reaction was only catalyzed by the well exfoliated, low concentrated FL‐BP dispersions and not by any BP sample at >1 mM concentration. *In‐situ* Raman and SRM studies (Figures S11 and 12) show the rapid disappearance of the characteristic KP_6_ intercalation bands at around 290 and 400 cm^−1^, which confirms a strong agglomeration of the KP_6_ flakes during reaction and explains the rapid deactivation of this extremely active but unstable catalyst (entry 9 in Table 1). These results confirm the need of an exfoliated BP material to catalyze the radical coupling.

**Figure 3:**
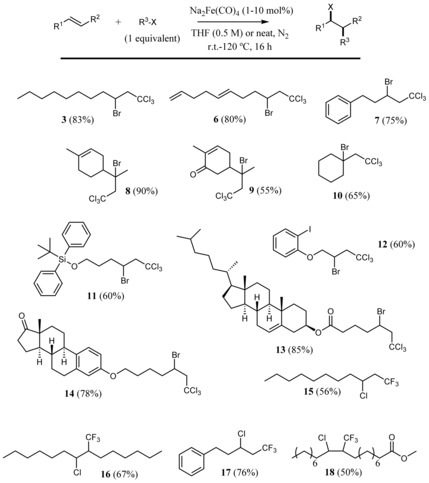
Radical additions of trihalomethyl halides to alkenes catalyzed by Na_2_Fe(CO)_4_. Specific reaction conditions: Products **3**–**14** isolated after 16 h at 25 °C in THF (0.5 M) with 10 mol% catalyst; products **15**–**18** isolated after 16 h at 120 °C under solventless conditions and with 1 mol% catalyst, CF_3_SO_2_Cl used as a reagent.

**Figure 4:**
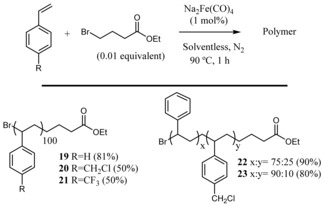
Radical polymerization catalyzed by Na_2_Fe(CO)_4_. Products **19**–**23** isolated after 1 h at 90 °C under solventless conditions and with 1 mol% catalyst. Polymerizations were not catalyzed by FL‐BP under the indicated reaction conditions.

At this point, since the more electron‐rich KP_6_ and KC_8_ catalyze the radical coupling between **1** and **2** faster than FL‐BP and graphene, respectively, different low‐valence Fe^1−^ and Fe^2−^ complexes^22^ were prepared and tested as catalysts, and compared with the benchmark catalyst Fe(CO)_5_. Table 1 (entry 11) shows the higher catalytic activity of Collman's reagent derivative Na_2_Fe_2_(CO)_8_ compared to Fe(CO)_5_ (see Table S1 for other low‐valence Fe complexes) and, by far, compared to other metal catalysts (entries 11–15, notice that these metal salts only catalyze the radical coupling with an excess of **2** under heating conditions). Low‐valence Fe complexes are routinely used as reagents for 2*e* ^−^ carbon−carbon couplings^23^ but not so used as catalysts for radical couplings.^24^ Kinetic studies (Figures S13 and S14) corroborate the high intrinsic catalytic activity of low‐valence Fe and indicate that Fe(CO)_5_ and Fe^2+^ salts may evolve under reaction conditions to the more active low‐valence Fe species, according to *in‐situ* Fourier‐transform infrared spectroscopy (FT‐IR, Figure S15), ^1^H and ^13^C NMR (Figure S16) and electrochemical measurements (Figures S17 and S18). Figure 3 shows that an array of bromo‐ and chloro‐substituted trichloro, and more challenging trifluoro‐, alkyl compounds^25^ **6**–**18** could be synthesized under these mild reaction conditions, i. e. equimolar amounts of alkene and alkyl halide at room temperature for the atom‐transfer radical addition (ATRA). Moreover, Figure 4 shows that, under similar reaction conditions, polymers **19**–**23** could be prepared after atom‐transfer radical addition polymerization (ATRP) reactions. The products are decorated with other functional groups such as hindered internal alkenes, ketones, acid‐sensitive silylethers and ethers, esters and other halides, which cannot be done with previously reported methodologies, particularly for natural products (Tables S2 and S3, see Refs. therein). Thus, it can be said that the observed increasing catalytic activity of 2D materials with electron richness also applies to Fe complex catalysts and enables a new synthetic procedure for sensitive organic compounds.

### Reaction mechanism

**Figure 5:**
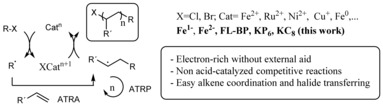
Plausible mechanism for the FL‐BP and low valence Fe‐catalyzed atom‐transfer radical addition (ATRA) and polymerization (ATRP) reactions. R=Ar, alkyl.

In order to determine if the electronic parallelism found for the main element 2D materials and metal complexes as catalysts for the radical coupling of **1** and **2** does not only occur in reactive but also in mechanistic terms, kinetic studies at different concentrations of reagents either with FL‐BP or Na_2_Fe(CO)_4_ catalyst were carried out (Figures S19 and S20, respectively). The results give the same experimental rate equation for both catalysts, i. e. v_0_=k_exp_[catalyst][**1**][**2**]. The nature and bonding of the halide atom transferred from **2** was studied by electrochemistry and the results show that only Br and not Cl anions are released during the coupling with both FL‐BP and Na_2_Fe(CO)_4_ catalysts,^26^ by a 1*e* ^−^ process (Figure S21).^27^, ^28^ Indeed, the results support the formation of a transient P−Br bond after radical dissociation of **2** 16a and then coupling with alkene **1**.^29^ This mechanistic proposition nicely engages with the ability of FL‐BP to couple with alkyl halides by temporal breaking of lattice P−P bonds^11^ and also with the easy chemically‐induced polymerization of alkenes.^30^ For low‐valence Fe complexes, this proposal is also validated by the crystallographic characterization of insertion products of perfluoromethane halides^31^ and alkyl chlorides^32^ in Na_2_Fe_2_CO_8_. In the case of FL‐BP, electron paramagnetic resonance (EPR) proves the radical mechanism, confirmed by the addition of the *N*‐tert‐butyl‐α‐phenylnitrone (PBN) spin trap, which is known to interact with CCl_3_ radicals.^33^ In the presence of FL‐BP in THF, a homolytic cleavage of the C−Br bond of CBrCl_3_ forms CCl_3_ radicals, which are rapidly trapped. The intensity gain of the PBN bands is a clear signal of the successful reduction of the molecule, and the characteristic N triplet signal (l=1) in Figure 2c appears due to a hyperfine coupling of the electron to the nitrogen nuclei. The additional splitting of the triplet is caused by H nuclei (l=1/2) in the spin adduct PBN−CCl_3_, and no other signals corresponding to the addition of Cl atoms to PBN are observed (Figure S22). Altogether, these results strongly support that the mechanism of both FL‐BP and low‐valence Fe catalysts for the radical coupling of **1** and **2** is essentially the same, also similar to typical metal catalysts, as shown in Figure 5.

### Other radical additions to alkenes

**Figure 6:**
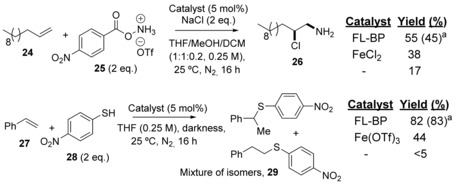
Other radical additions to alkenes catalyzed by FL‐BP. Reaction conditions optimized for FL‐BP. GC yields. Tf: trifluoromethanesulfonate. [a] Between brackets, yields at ten‐time higher scale (0.5 and 1 mmol, respectively).

Figure 6 shows the use of FL‐BP as a catalyst for other radical additions to alkenes typically catalyzed by Fe compounds.^34^ The results confirm the higher catalytic activity of (FL‐BP) for these radical couplings compared to the representative Fe‐based catalysts, under optimized conditions for FL‐BP and under reasonable mass scales. Notice that **25** and **28** were used as coupling partners since nitrobenzene derivatives better adsorb and react on the FL‐BP surface, thus taking advantage of the adsorption properties of FL‐BP to carry out the catalysis.12a

## Conclusions

The 2D material FL‐BP catalyzes with extraordinary activity different radical additions to alkenes. The electron‐rich counterpart KP_6_ is even more catalytically active, and this increase in catalytic activity with electron richness also applies not only to other main element 2D materials such as graphene (vs. KC_8_) but also to low‐valence Fe complexes (vs. Fe^0^). The use of catalytic FL‐BP constitutes a seminal and very promising starting point to design efficient radical catalysts based in 2D *p*‐block elements, and the parallelism found between 2D materials and Fe complexes opens the door for cross‐fertilization studies between these two apparently separated catalytic areas.

## Experimental Section

**Synthesis of FL‐BP**: LPE under inert conditions was achieved by tip sonication in an argon‐filled glovebox (<0.1 ppm O_2_ and <0.1 ppm H_2_O). The starting concentration of BP was 2 mg mL^−1^ in NMP according tot he manual 80 % is about 56 W effective power and the FL‐BP was achieved by using a Bandelin Sonoplus 3100, 80 % amplitude, four intervals of 30 min (2 h in total), pulse 2 s on, 2 s off, stirring and cooling the dispersion (0 °C) to avoid high temperatures and the decomposition of BP flakes. After the exfoliation, the FL‐BP dispersion were centrifuged for 1 h. at 1753 g, the supernatant was transferred to a new vial and the dispersions were further centrifuged at 21475 g. The FL‐BP precipitate was separated from NMP and re‐dispersed in anhydrous THF. Solvent exchange was achieved after repeating the last process 3 times, ensuring that most of the NMP has been exchanged in the dispersion. The concentration of the dispersion was quantified by ICP, presenting 0.001 wt% of phosphorus. The concentration of the dispersions can be modified by appropriated dilutions.

**Typical reaction procedure for the reaction between 1‐decene 1 and CBrCl_3_ 2 with FL‐BP**: In the glove box, 0.5 ml of FL‐BP (0.005 mol%) in dry THF was placed in a 2 mL vial equipped with a magnetic stirrer. Then, 1‐decene **1** (50 μl, 0.5 mmol, 1 eq) and CBrCl_3_ **2** (25 μl, 0.5 mmol, 1 eq) were added. The reaction mixture was magnetically stirred at room temperature for 16 h. At the end of the reaction, the crude product was purified by column chromatography eluting with heptane or hexane/ethyl acetate to give the product as a clear oil (87 mg, 0.26 mmol, 51 %), as analyzed by GC, GC‐MS, and NMR spectroscopy.

## Conflict of interest

The authors declare no conflict of interest.

## Supporting information

As a service to our authors and readers, this journal provides supporting information supplied by the authors. Such materials are peer reviewed and may be re‐organized for online delivery, but are not copy‐edited or typeset. Technical support issues arising from supporting information (other than missing files) should be addressed to the authors.

Supplementary
