# Supplementary material for: Few‐layer Black Phosphorous Catalyzes Radical Additions to Alkenes Faster than Low‐valence Metals
Source: ChemCatChem. 2020 Feb 25;12(8):2226–32. doi: 10.1002/cctc.201902276 (PMC7216949; doi:10.1002/cctc.201902276)
Supplement: Supplementary file 1 — Supplementary [file CCTC-12-2226-s001.pdf]

ChemCatChem

Supporting Information

Few-layer Black Phosphorous Catalyzes Radical Additions to Alkenes Faster than Low-valence Metals

María Tejeda-Serrano, Vicent Lloret, Bence G. Márkus, Ferenc Simon, Frank Hauke, Andreas Hirsch, Antonio Doménech-Carbó,* Gonzalo Abellán,* and Antonio Leyva-Pérez*© 2020 The Authors. Published by Wiley-VCH Verlag GmbH & Co. KGaA.
This is an open access article under the terms of the Creative Commons Attribution License, which permits use, distribution and reproduction in any medium, provided the original work is properly cited.

Table of Contents

Experimental Procedures	*SI3*
General	SI3
Solvent and catalyst preparation	SI3
Synthesis of alkene precursors	SI3
Electrochemistry	SI4
Reaction procedures	SI4
Results and Discussion	*SI6*
Tables S1–S3	SI6
Figures S1–S22	SI7
Compound characterization	*SI25*
NMR spectra	SI29
References	*SI59*
Author contributions	*SI60*

Experimental Procedures

General. Reagents were obtained from commercial sources (Merck–Aldrich) and were used without further purification otherwise indicated. Dried and deareated solvents were obtained after treatment with purification resins, having less than 50 ppm of water. All the products obtained were characterised by GC–MS, ^{1}H–, ^{13}C–, and ^{19}F–NMR, and DEPT. When available, the characterisation given in the literature was used for comparison. Gas chromatographic analyses were performed in an instrument equipped with a 25 m capillary column of 1% phenylmethylsilicone. GC/MS analyses were performed on a spectrometer equipped with the same column as the GC and operated under the same conditions. ^{1}H, ^{13}C, DEPT and ^{19}F were recorded in a 7T instrument using the appropriate solvent with a suitable internal standard (TMS or hexafluorobenzene). Absorption spectra were recorded on an UV/Vis spectrophotometer (UV0811M209, Varian). Solid IR spectra of the compounds were recorded on a Jasko 460 plus spectrophotometer by previous mixture with KBr. IR spectra of the liquids were recorded on a Jasko 460 plus spectrophotometer by impregnating the windows with a dichloromethane solution of the compound and leaving to evaporate before analysis. IR peaks are defined as very intense (vi), intense (i), medium (m), low (l) and broad (br).

Solvent preparation.
NMP and THF anhydrous were purchased from Sigma Aldrich Co. (Germany). NMP and THF were degassed using the "Freeze–Pump–Thaw" method to remove oxygen, before introduced into the glove box (ca. 7 cycles). THF was further distilled over a Na/K alloy to ensure that the water content was <1ppm. The water content was determined by a coulometric Karl Fischer titrator.
Catalyst preparation.
Synthesis of FL–BP: LPE under inert conditions was achieved by tip sonication in an argon–filled glovebox (<0.1 ppm O_2 and <0.1 ppm H_2O). The starting concentration of BP was 2 mg mL^{-1} in NMP and the FL–BP was achieved by using a Bandelin Sonoplus 3100, 80% amplitude, four intervals of 30 min (2h in total), pulse 2 s on, 2 s off, stirring and cooling the dispersion (0°C) to avoid high temperatures and the decomposition of BP flakes. After the exfoliation, the FL–BP dispersion were centrifuged for 1h. at 1753 g, the supernatant was transferred to a new vial and the dispersions were further centrifuged at 21475 g. The FL–BP precipitate was separated from NMP and re–dispersed in anhydrous THF. Solvent exchange was achieved after repeating the last process 3 times, ensuring that most of the NMP has been exchanged in the dispersion. The concentration of the dispersion was quantified by ICP, presenting 0.001 wt% of phosphorus. The concentration of the dispersions can be modified by appropriated dilutions.
Synthesis of KP_6: Sample preparation was carried out in an argon–filled LabMaster Pro sp glovebox (MBraun), equipped with a gas purifier and solvent vapor removal unit, with an oxygen and water content <0.1 ppm. Firstly the BP crystals (obtained from smart elements) were thoroughly grinded to obtain a homogeneous fine powder, then we mixed progressively the BP with a stoichiometric amount of potassium (1/6 eq.) in glass vials at controlled temperature. Once the materials were in close contact, the temperature was slowly increased until 70 °C stirring gently the material. Finally, the samples were homogenized by a thermal treatment for ca. 48 hours. Once the intercalation was achieved, 5 mL of distilled THF were added and the dispersions were tip–sonicated for 15 min (0.2 on/off pulse at 25%).[1]
Synthesis of KC_8: Sample preparation was carried out in an argon–filled Labmasterpro sp glovebox (MBraun), equipped with a gas purifier and solvent vapor removal unit, with an oxygen and water content <0.1 ppm. Firstly, the SGN18 graphite powder/graphite crystal was thoroughly grinded to obtain a homogeneous fine powder, then we mixed progressively the graphene with a stoichiometric amount of potassium (1/8 eq.) in glass vials at controlled temperature. Once the materials were in close contact, the temperature was slowly increased until 200 °C stirring the material. Finally, the samples were homogenized by a thermal treatment for ca. 48 hours. Once the intercalation was achieved, 5 mL of distilled THF were added and the dispersions were tip–sonicated for 15 min (0.2 on/off pulse at 25%).[2]
Synthesis of the $Na_2Fe_2(CO)_8$–4THF: Under nitrogen atmosphere, $Na_2Fe(CO)_4$–1.5 dioxane (1.022 g, 2.95 mmol, 1 eq) was added to a dry bottom flask equipped with a magnetic stirrer. Then, a solution of $Fe(CO)_5$ (388 µl, 2.95 mmol, 1 eq) in THF (20 ml) was added to the solid and magnetically stirred at room temperature overnight. The precipitate formatted was dried under a nitrogen flow at 40 ºC to obtain the product as a brown solid (1.80 g, 90%).
Synthesis of Fe complex $[(Me_4Fe)(MeLi)][Li(OEt_2)]_2$: A solution of $FeCl_3$ (0.595 g, 3.67 mmol, 1 eq) in Et_2O anhydrous (10 ml) was slowly added to a solution of MeLi (1.6 M in Et_2O, 11.2 ml, 17.9 mmol, 4.8 eq) in Et_2O (5 ml) at -78 °C. The resulting dark brown mixture was stirred at that temperature for 4 h and then for another 4 h at -30 °C, whereby colour changes to red–brown. The precipitated salts were filtered off under N_2 flow at -30 °C, the resulting filtrate was concentrated and then kept at –30 °C for 1 d. The precipitated complex was obtained in traces and stored at -30 ºC. However, the product is thermally unstable even under inert atmosphere at temperatures above 0°C and vigorously ignites in air.

<u>Synthesis of alkene precursors.</u>
Synthesis of alkene S1: In a round bottom flask equipped with a magnetic stirrer, imidazole (378 mg, 5.5 mmol, 1.1 eq) was dissolved in dry CH_2Cl_2 (3 mL). Then, 4–penten–1–ol (521 µl, 5 mmol, 1eq) and TBDPSCl (1.5 ml, 5.5 mmol, 1.1eq) were added to the solution. A white solid appears and the reaction was stirred at room temperature during 16 h. After this time, the reaction mixture was diluted with Et_2O (20 mL) and washed with brine (2 × 20 mL). The organic layer was dried with $MgSO_4$ anhydrous, filtered and concentrated. The residue was purified by column chromatography eluting with *n*–hexane to give the pure product as a white solid (1.43 g, 88%).
Synthesis of alkene S2: In a round bottom flask equipped with a magnetic stirrer, 2–iodophenol (1.10 g, 5.0 mmol, 1 eq) and potassium carbonate (0.7 g, 5.0 mmol, 1.0 equiv) were dissolved in DMF (10 mL). Then, allyl bromide (525 µl, 6.0 mmol, 1.2 eq) was slowly added to the solution. The reaction mixture was allowed to stir at room temperature for 16 h. The reaction mixture was quenched with H_2O (3

mL) and extracted with ethyl acetate (3 x 15 mL). The combined organic layer was dried with $MgSO_4$ anhydrous, filtered and concentrated. The residue was purified by column chromatography eluting with *n*–hexane and ethyl acetate (20:1) to give the pure product as a yellowish oil (1.24 g, 95%).

Synthesis of alkene S3: In a round bottom flask equipped with a magnetic stirrer, 5–hexanoic acid (606 µl, 5 mmol, 1eq) was dissolved in dry CH_2Cl_2 (5 mL). Then, 4–(dimethylamino)pyridine (62 mg, 0.5 mmol, 0.1 eq) and cholesterol (2.3 g, 6 mmol, 1,2 eq) were added to the solution. The reaction mixture was cooled to 0ºC and *N,N'*–dicyclohexylcarbodiimide (1.2 g, 5.5 mmol, 1.1 eq) was added. The reaction mixture was stirred for 5 min at 0°C and 3 h at room temperature. After this time, precipitated urea was filtered off and the filtrate was concentrated. The residue was solved in CH_2Cl_2 and, if necessary, filtered free of any further precipitated urea. The CH_2Cl_2 solution was washed 0.5M HCl (2 x20 mL) and with saturated $NaHCO_3$ solution (20 mL). The organic layer was dried with $MgSO_4$ anhydrous, filtered and concentrated. The residue was purified by column chromatography eluting with a mixture of *n*–hexane and ethyl acetate (90:10) to give the pure product as a yellowish solid (1.84 g, 76%).

Synthesis of alkene S4: In a round bottom flask equipped with a magnetic stirrer, estrone (500 mg, 1.8 mmol, 1eq) and K_2CO_3 (253 mg, 1.8 mmol, 1 eq) were dissolved in dry ACN (10 mL). Then, 6–bromo–1–hexene (260 µl, 1.8 mmol, 1 eq) was added to the solution. The reaction mixture was heated to reflux and was stirred for 16 h. After this time, the reaction mixture was cooled to room temperature and was diluted with CH_2Cl_2 (20 mL), washed with distilled water (2 × 20 mL) and saturated $NaHCO_3$ solution (20 mL). The organic layer was dried with $MgSO_4$ anhydrous, filtered and concentrated. The residue was purified by column chromatography eluting with a mixture of *n*–hexane and ethyl acetate (90:10) to give the pure product as a white solid (125 mg, 20%).

Electrochemistry.

With Fe complexes. Voltammetric measurements were performed at glassy carbon working electrode in solutions of the different compounds and their mixtures in 0.10 M Bu_4NPF_6/MeCN. A CH I660 potentiostat was used in an electrochemical cell using Pt mesh auxiliary electrode and a Pt wire pseudo–reference electrode to avoid water contamination. The measured potentials can be passed to the ferrocene/ferrocenium scale by subtracting 0.16 V. The solutions were optionally deaerated by bubbling Ar for 10 min.

With FL–BP. Voltammetric experiments were performed using a CH 660c equipment using 10 mM DMSO and MeCN solutions of 1–decene, $CBrCl_3$, and/or a 0.002 mM solution of FL–BP optionally de–aerated by bubbling Ar for 15 min. Bu_4NPF_6 was used as a supporting electrolyte. Glassy carbon electrode (geometrical area 0.071 cm^2) was used as a working electrode, completing the three–electrode arrangement with a Pt mesh counter electrode and a Pt wire pseudo–reference electrode. Ferrocene was used as an internal standard for potential measurements.

Reaction procedures.

Typical reaction procedure for the reaction between 1–decene 1 and $CBrCl_3$ 2 with FL–BP at 5 mmol scale: In the glove box, 5 ml of FL–BP (0.005 mol%) in dry THF was placed in a 2 mL vial equipped with a magnetic stirrer. Then, 1–decene **1** (0.5 ml, 5 mmol, 1 eq) and $CBrCl_3$ **2** (250 µl, 5 mmol, 1 eq) were added. The reaction mixture was magnetically stirred at room temperature for 16 h. At the end of the reaction, the crude product was purified by column chromatography eluting with heptane or hexane/ethyl acetate to give the products as a clear oil, in similar yields to 0.5 mmol reactions, as analysed by GC, GC–MS, and NMR spectroscopy.

Typical reaction procedure for the reaction between 1–decene 1 and $CBrCl_3$ 2 with other solid catalysts: In a 2 mL vial equipped with a magnetic stirrer, the solid catalyst (0.025–0.25 mmol, 5–50 mol%) was placed under nitrogen atmosphere, and dry THF (0.5 mL) was added. Then, 1–decene **1** (50 µl, 0.5 mmol, 1 eq) and $CBrCl_3$ **2** (25 µl, 0.5 mmol, 1 eq) were added. The reaction mixture was magnetically stirred at room temperature for 16 h. At the end of the reaction, the crude product was purified by column chromatography eluting with heptane or hexane/ethyl acetate to give the products as a clear oil, as analysed by GC, GC–MS, and NMR spectroscopy.

Typical reaction procedure for the reaction between 1–decene 1 and $CBrCl_3$ 2 with metals: In a 2 mL vial equipped with a magnetic stirrer, $Na_2Fe(CO)_4$–1.5 dioxane (8.6–17.3 mg, 0.025–0.05 mmol, 5–10 mol% Fe) was placed under nitrogen atmosphere, and dry THF (0.5 mL) was added. Then, 1–decene **1** (50 µl, 0.5 mmol, 1 eq) and $CBrCl_3$ **2** (25 µl, 0.5 mmol, 1 eq) were added. The reaction mixture was magnetically stirred at room temperature for 16 h. At the end of the reaction, the crude product was purified by column chromatography eluting with heptane or hexane/ethyl acetate to give the products as a clear oils, as analysed by GC, GC–MS, and NMR spectroscopy.

Typical reaction procedure for trifluoromethylation reactions: In a double–walled 6 mL vial equipped with a magnetic stirrer, $Na_2Fe(CO)_4$–1.5 dioxane (3.2 mg, 0.0093 mmol, 1 mol% Fe) was placed under nitrogen atmosphere, and the reactor was closed with a screw cap connected to a manometer. Then, 1–decene **1** (187 µL, 0.93 mmol, 1 eq) and CF_3SO_2Cl (100 µL, 0.93 mmol, 1 eq) were added. The reaction mixture was placed in a pre–heated oil bath at 120 °C and magnetically stirred for 4−16 h. A pressure increase of 2 bar was observed, caused by the SO_2 formed during reaction. After that time, the crude product was purified by column chromatography eluting with heptane or hexane/ethyl acetate to give the product **15** as a clear oil (136 mg, 0.46 mmol, 56%), as analysed by GC, GC–MS, and NMR spectroscopy.

Typical reaction procedure for chloroamination of 1–dodecene 24: In a 2 mL vial equipped with a magnetic stirrer, 1–dodecene **24** (11 µL, 0.05 mmol, 1 eq), nitroderivative **25**[3] (33.6 mg, 0.1 mmol, 2 eq) and NaCl (6 mg, 2 eq.) were added. Then, the corresponding catalyst and the solvent mixture (THF/MeOH/DCM, 1:1:0.2, 0.25M) was added in a dry box (a partial concentration of the FL–BP dispersion to the required one was previously carried out), and the vial was sealed. Then, the reaction mixture was taken out of the dry box and magnetically stirred at room temperature for 16 h. After that time, the crude product was treated with an aqueous 1M solution of NaOH (210 µL, 0.2 mmol), extracted with DCM (twice) and analysed by GC and GC–MS using *n*–decane as an external standard. The same reaction was performed at ten times more scale, to give a similar but slightly lower yield (ca. 10% lower).

Typical reaction procedure for hydrothioalkoxylation of styrene 27: In a 2 mL vial equipped with a magnetic stirrer, styrene **27** (14 μL, 0.1 mmol, 1 eq), *para*–nitrothiophenol **28** (31 mg, 0.2 mmol, 2 eq), the corresponding catalyst and THF (0.8 mL, 0.25M, a partial concentration of the FL–BP dispersion to the required one was previously carried out) were added in a dry box, and the vial was sealed. Then, the reaction mixture was taken out of the dry box and magnetically stirred at room temperature for 16 h. After that time, the crude product was filtered if needed and analysed by GC and GC–MS using *n*–dodecane as an external standard. The same reaction was performed at ten times more scale, to give a similar GC yield.

Other techniques.

Raman spectroscopy: Raman spectra were acquired on a WITec's Raman microscope alpha300 R confocal microscope equipped with an automated XYZ stage. All measurements were conducted using an excitation wavelength of 532 nm, with an acquisition time of 1 s and gratings of 600 and 1800 grooves/mm depending on the experiment. The used laser intensity was 3 mW. For Raman mappings, the step sizes were in the 0.2–0.5 μm range depending on the experiments, and areas of 50x50, 30x30 and 5x5 μm were measured.

Atomic Force Microscopy: AFM was carried out using a Bruker Dimension Icon microscope in Scan–Asyst mode. The samples were prepared by spin coating a solution of a given sample at 5000 rpm. Bruker Scanasyst–Air silicon tips on nitride levers with a spring constant of 0.4 N/m were used to obtain images resolved by 512 × 512 or 1024 × 1024 pixels.

Absorption Spectroscopy: The absorbance of the samples was measured on a Perkin Elmer Lambda 1050 spectrometer in extinction mode, in quartz cuvettes with a path length of 0.4 cm. The cuvettes were precisely sealed inside the glovebox with a cup and parafilm in order to avoid oxygen.

Electron paramagnetic resonance: EPR spectra were recorded on a commercial Magnettech MS–300 X–band spectrometer equipped with a rectangular TE102 microwave cavity, operated at room temperature. During the measurements the microwave power was 0.5 mW to avoid saturation of the lines. Quartz ampules were filled in with 1 mL of the different samples and were kept under Ar during the EPR measurements.

Results and Discussion

Tables.

Table S1 Additional catalyst screening for the radical coupling between **1** and **2**. See reaction scheme in the main text. The indicated reaction conditions for each catalyst are the mildest found to achieve full conversion and highest selectivity of **3**.

Entry	Catalyst	Solvent	2 (eq)	T (ºC)	TOF_0 (s^{-1})	Yield of 3 (%)
1	$FeCl_2$	**2** (0.15M)	20	50	–	–
2				90	0.001	70
3	$Fe(acac)_3$			90	0.001	12
4	CuCl			100	–	–
5	$NiCl_2$			50	–	–
6				100	0.003	95
7	$RuCl_2(PPh_3)_2$			25	–	–
8				50	0.005	80
9	Ferrocene			50	–	–
10				90	0.015	40
11	$Fe(CO)_5$			25	0.001	98
12				–5	0.002	4
13	Ferrocene	Toluene (0.5M)	1	90	0.012	60
14	$Fe(CO)_5$			25	0.012	75
15	CuCl			50	–	–
16				100	0.001	10
17	$NiCl_2$			50	–	–
18				100	0.003	77
19	$RuCl_2(PPh_3)_2$			25	–	20
20				50	0.005	30
21	$Fe(CO)_5$	CH_3CN (0.5M)		25	0.015	75
22		CH_2Cl_2 (0.5M)		25	0.001	13
23		Hexane (0.5M)		25	<0.001	20
24		THF (0.5M)		25	0.014	60
25	$Na_2Fe(CO)_4$			25	0.004	72
26	$Na_2Fe_2(CO)_8$			25	0.018	81
27	$[(Me_4Fe)(MeLi)][Li(OEt_2)]_2$			–10	0.001	80
28	$Fe(MgPh)_2$			25	0.001	43

Table S2 Representative catalysts in the literature for the Kharasch reaction, with substrates converted in reasonable yields (>50%) under the indicated reaction conditions.

Entry	Catalyst	T (ºC)	Solvent	Substrates	Reference
1	$(AcO)_2$, $h\nu$, iodine	60–70	$CBrCl_3$	Styrene, 1–octene, 2–octene, vinyl acetate, isobutilene, propilene, ethylene, allyl chloride	[4]
2	$FeCl_2/CuCl_2$ Bz_2O_2, reducing agent	80	MeOH MeCN PrOH	Buten–2–ene, buten–1–ene, oct– l–ene, oct–2–ene, styrene, acrylonitrile, methyl acrylate, ethyl acrylate	[5]
3	$Fe(CO)_5$ $Me_3NFe(CO)_4$	25	$CBrCl_3$	terminal keto–alkenes and cyclic alkenes	[6]
4	Fe (solid)	80	DMF DCE	1–octene, butane, hexene	[7]
5	CuCl/tertiary amines	80	DCE	ω–alkenyl–trichloroacetamides	[8]
6	$(PCy_3)_2Cl_2RuCH{=}Ph$	65–80	$CHCl_3$	Styrenes and acrylates	[9]
7	$Fe(acac)_3/Et_3N$	80	Toluene	1–Hexene, ciclohexene	[10]
8	$[Rh(cod)Cl]_2$/phosphines	–78	Toluene/hexane	Styrenes, alkenyl pyridines and indoles	[11]

Table S3 Results for sensitive compounds under typical Kharasch reaction conditions with $FeCl_2$ catalyst (5 mol%, $CBrCl_3$ as a solvent, 90 ºC).

Entry	Reactant	Results
1	Limonene	The reactant was totally degraded and product was not formed
2	Carvone	The reactant was partially degraded and product was not formed
3	S3	The product was isomerized

Figures.

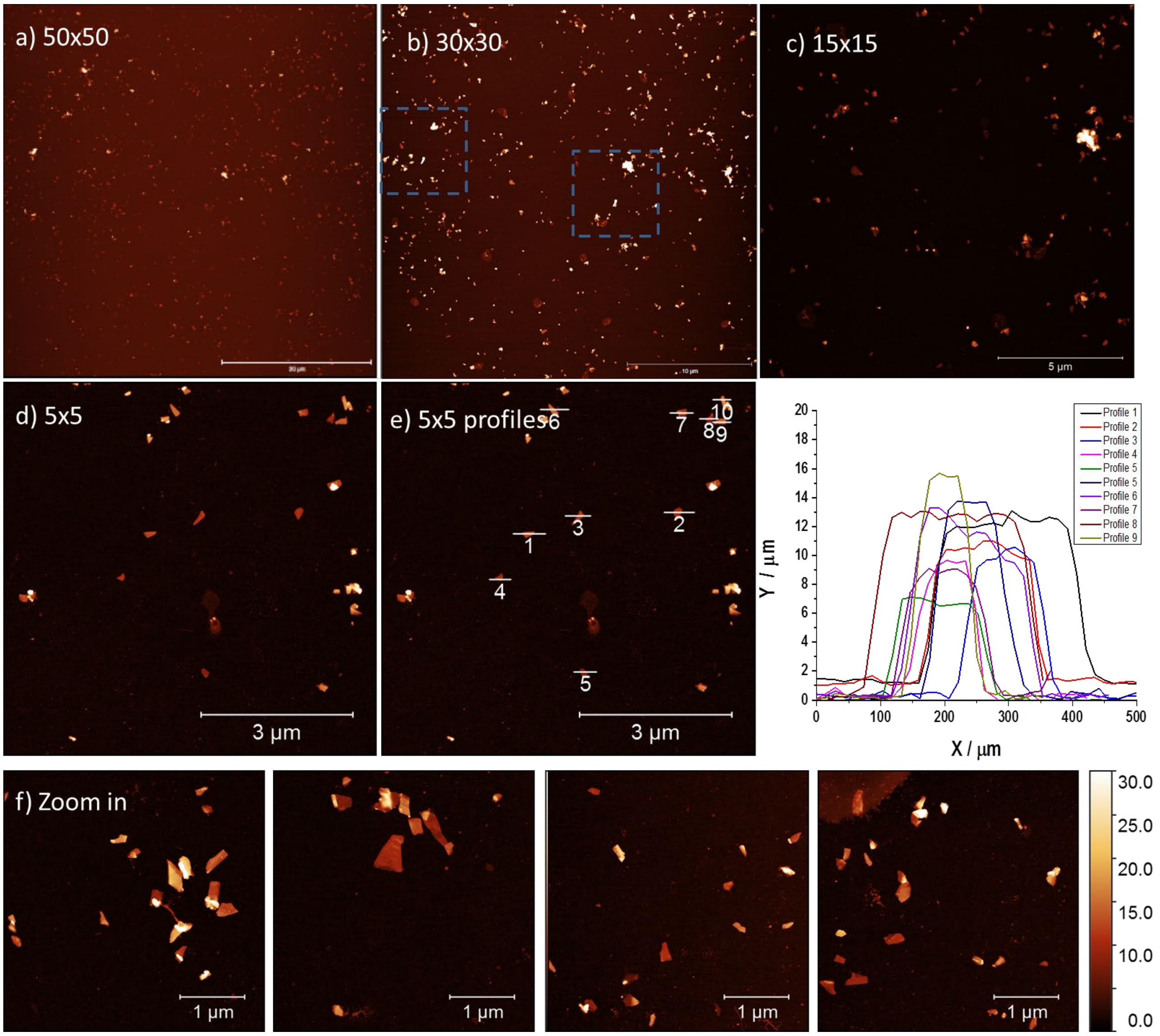

Figure S1. AFM images on Si/SiO$_2$ wafers of FL–BP flakes exfoliated in NMP and further transferred to THF. Several measurements of lower X/Y dimensions (b: 30x30, c: 15x15 and d: 5x5 µm) were performed in order to show the homogeneity of the sample and the appearance of the BP flakes. The last measurement e: 5x5 µm also shows profile lines of the height of the exfoliated material from 6 to 15 nm and lateral dimensions of 50 to 250 nm. f) 4x4 µm zoom in of several flakes in order to show the defined shape and structure of the exfoliated material.

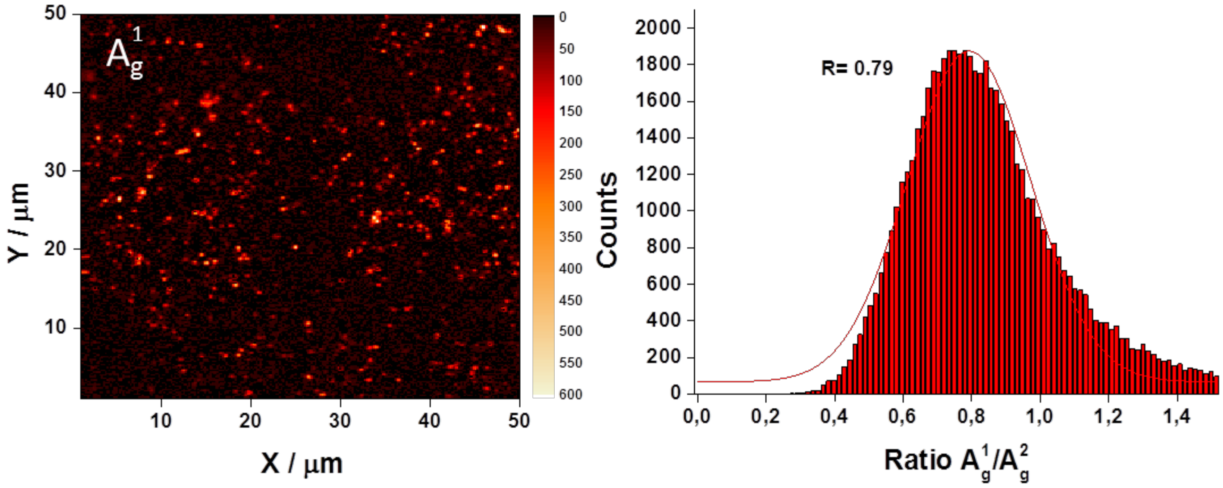

Figure S2. a) Scanning Raman microscopy map (50x50 µm) of the A^1_g Raman mode of BP. b) presents the ratio between the A^1_g/A^2_g BP modes to prove that the material is not oxidized after the sonication and solvent exchange processes. The small particles have a very low signal to noise ratio which leads to heavy tail distributions with values higher than R=1.

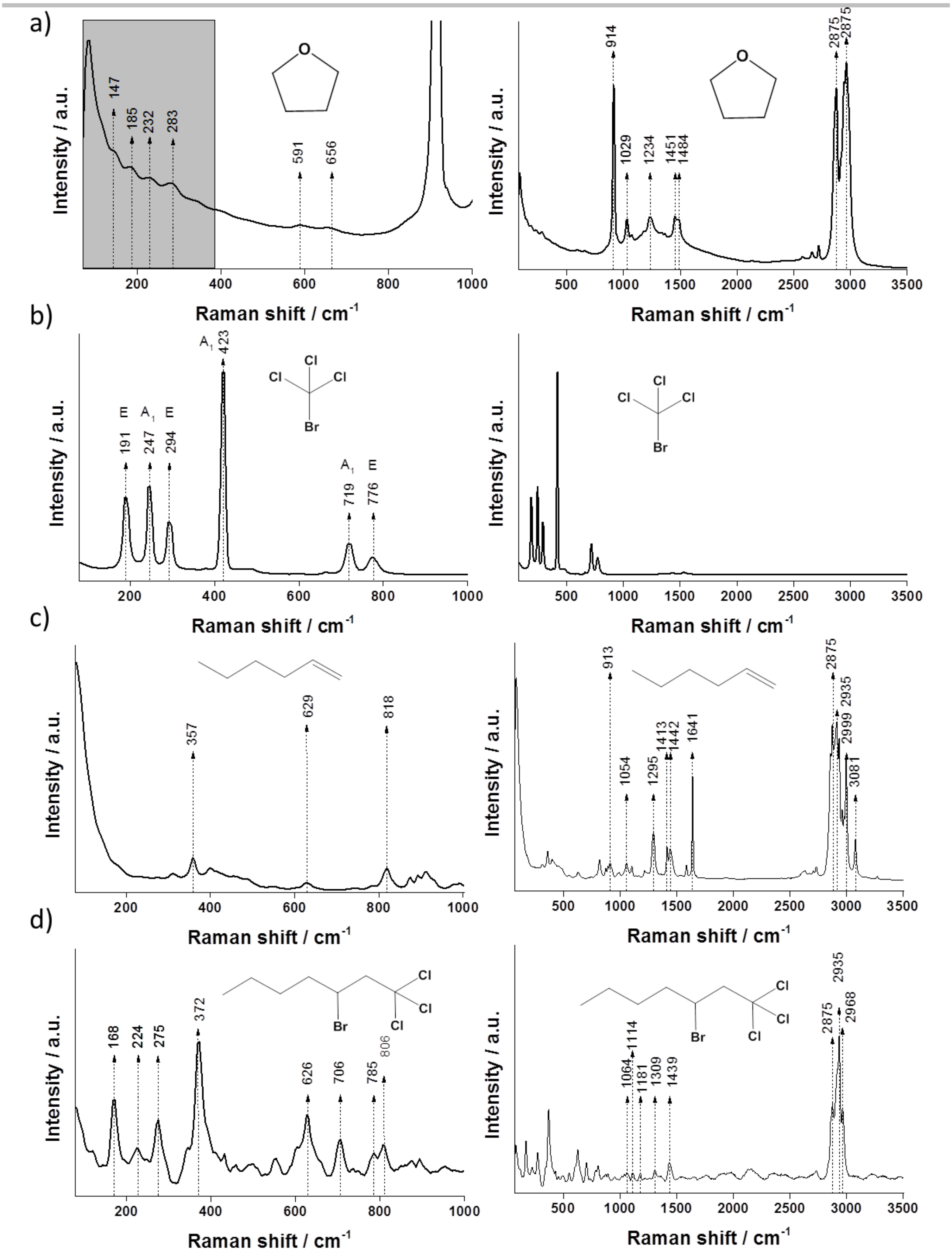

Figure S3. Liquid Raman spectroscopy study of the solvents and reactive used in the reactions. a) Shows the THF Raman spectra with a zoom in the range from 100 to 850 cm^{-1} where the most important Raman bands appear during the reaction, the small intensity peaks from 147 to

283 cm^{-1} appear in the rest of spectra which contain THF. On the right the whole spectra from 100 to 3500 cm^{-1} is shown with the respective C–C and C–O vibrations. b) Raman spectra of $CBrCl_3$ with a zoom in in the range from 100 to 850 cm^{-1} where the most important Raman bands appear during the reaction. The vibrations at 191, 247, 423 and 776 cm^{-1} correspond to degeneracy and symmetric vibrational modes of the Cl–C–Cl and C–Cl bonds. The bands at 294 and 719 cm^{-1} correspond to degeneracy vibrational modes of the Br–C–Cl and C–Br bonds respectively. On the right the whole spectra from 100 to 3500 cm^{-1} is shown without any C–C vibrational Raman modes as expected. c) Raman spectra of 1–hexene with a zoom in in the range from 100 to 850 cm^{-1} where the most important Raman bands appear during the reaction. The vibrations at 357, 629, 818 cm^{-1} have very small intensities and cannot be seen during the reaction. On the right, the whole spectra from 100 to 3500 cm^{-1} is shown, presenting a strong and polarized alkene C=C stretching at 1641 cm^{-1} and several alkyl vibrations at higher reciprocal cm (2999 and 3081 cm^{-1}). d) Raman spectra of the product 3–bromo–1,1,1–trichloroheptane with a zoom in in the range from 100 to 850 cm^{-1} where the most important Raman bands appear during the reaction. New vibrational modes appear at 168, 224, 275, 379, 626, 706, 785 cm^{-1} which correspond to degeneracy and symmetric vibrational modes of the Cl–C–Cl, C–Cl, Br–C–Cl and C–Br bonds. Due to the scarce literature on this product, the Raman modes cannot be assigned. On the right the whole spectra from 100 to 3500 cm^{-1} is shown which contains several alkyl vibrations at higher reciprocal cm^{-1}).

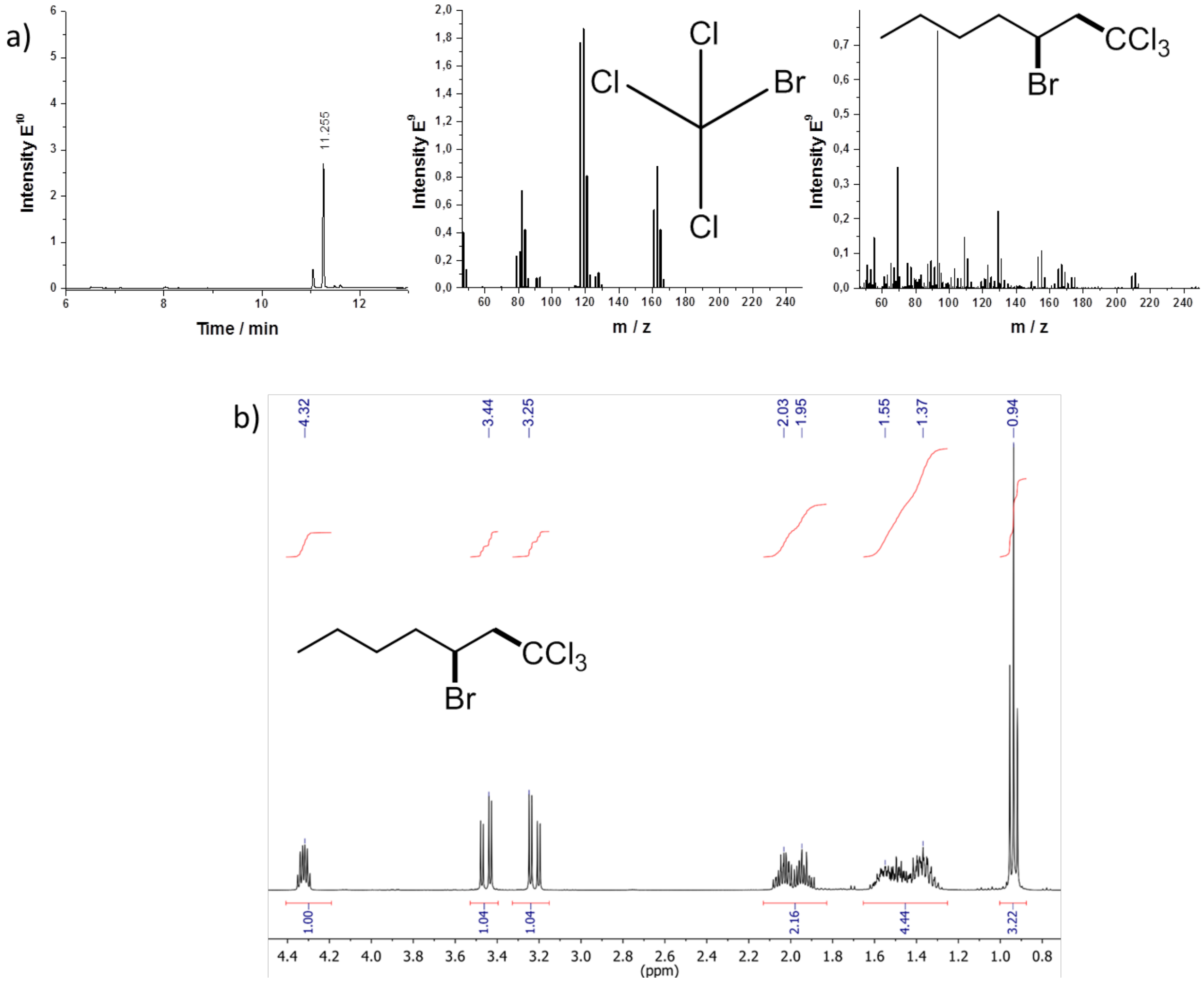

Figure S4. a) GC/MS spectra and b) NMR of the reaction product from the *in–situ* Raman experiment to prove that the observed Raman modes correspond to the expected 3–bromo–1,1,1–trichloroheptane, **5**.

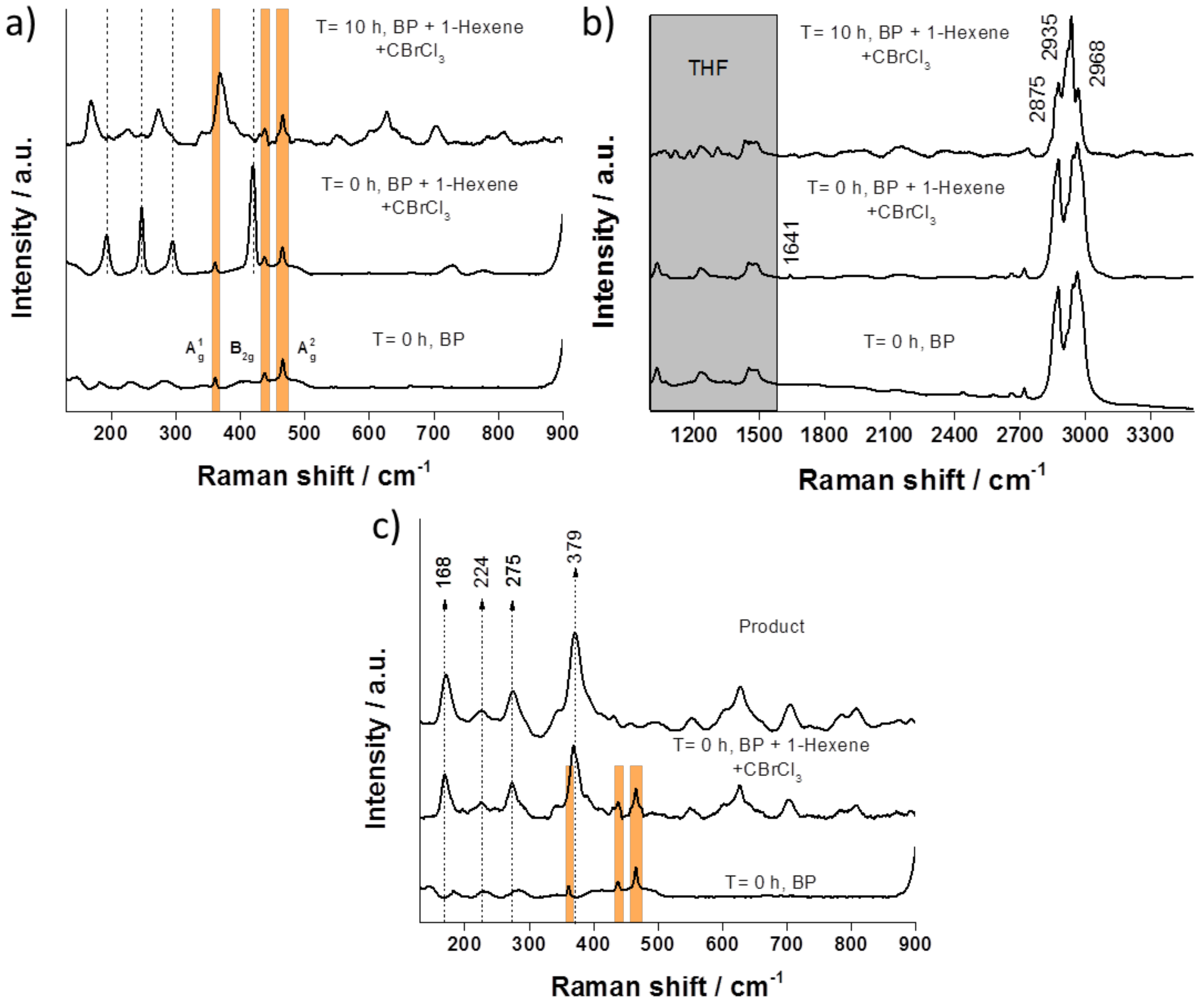

Figure S5. Liquid Raman spectroscopy study of the reaction between 1–hexene and $CBrCl_3$ catalysed by FL–BP. a) Raman spectra of BP in THF at T=0 h, Raman of the reaction mixture at T=0h and Raman of the reaction after T= 10h in which both, the BP and product Raman modes are observed. b) Raman modes at T= 0 and 10 h. The THF modes are stressed in grey and in this regime of the spectra is possible to visualize the corresponding peak of 1–hexene at 1641 cm^{-1}. After the reaction, the Raman modes corresponding to the product at around 2900 cm^{-1} can be distinguished from the THF ones. c) Difference between BP in THF, the reaction mixture and the pure product in which the BP modes are no longer observed.

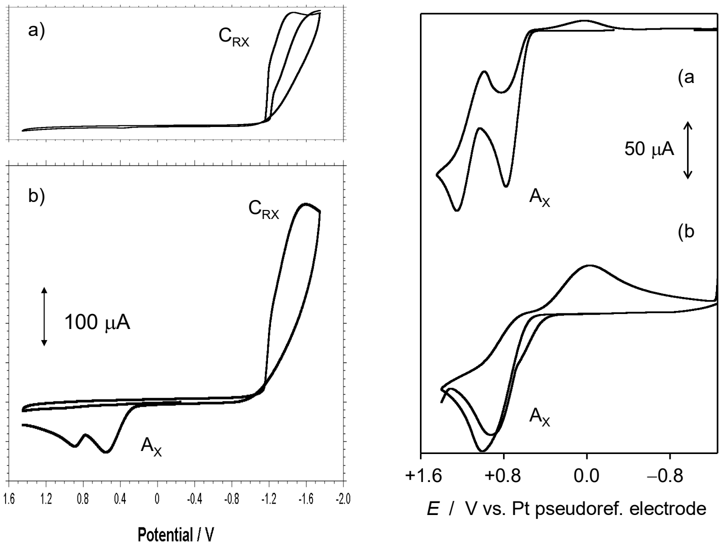

Figure S6. *Left:* a,b) Cyclic voltammograms at glassy carbon electrode for a 20 mM solution of $CBrCl_3$ **2** in 0.10 M Bu_4NPF_6/MeCN. Potential scan initiated at 0.0 V vs. Pt pseudo–reference electrode in the negative direction; potential scan rate: a) 50; b) 500 mV s^{-1}. *Right:* Cyclic voltammograms at glassy carbon electrode of 5 mM solutions of a) Et_4NBr and b) $Et_4NCl{\cdot}H_2O$ in 0.10 M Bu_4NPF_6/MeCN. Potentials vs. Pt pseudo–reference electrode. Potential scan rate 50 mV s^{-1}.[12],[13] The voltammogram of neat **2** shows the reductive cleavage of the C–Br bond and the 1e^- oxidation of Br^- to give Br_2 in the subsequent cathodic scan which, by comparison with Et_4NBr and Et_4NCl, confirms that only Br and not Cl anions are released.

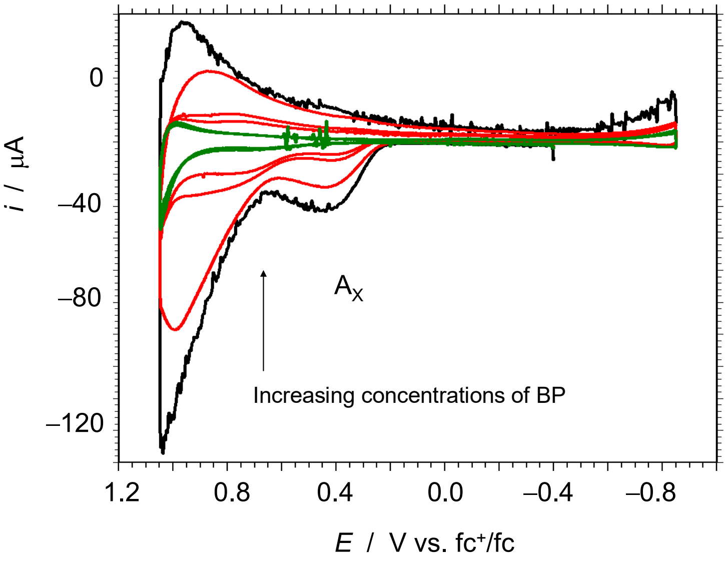

Figure S7 Region of the A_X signals of cyclic voltammograms at glassy carbon electrode of a 10 mM decene **1** plus 10 mM $CBrCl_3$ **2** solutions in 0.10 M Bu_4NPF_6/DMSO after adding increasing concentrations of FL–BP (denoted as BP in the Figure) from lower (black), middle (red) and higher (green) concentration. Potential scan rate 50 mV s^{-1}. Semi–derivative convolution of data is performed in order to enhance peak resolution.

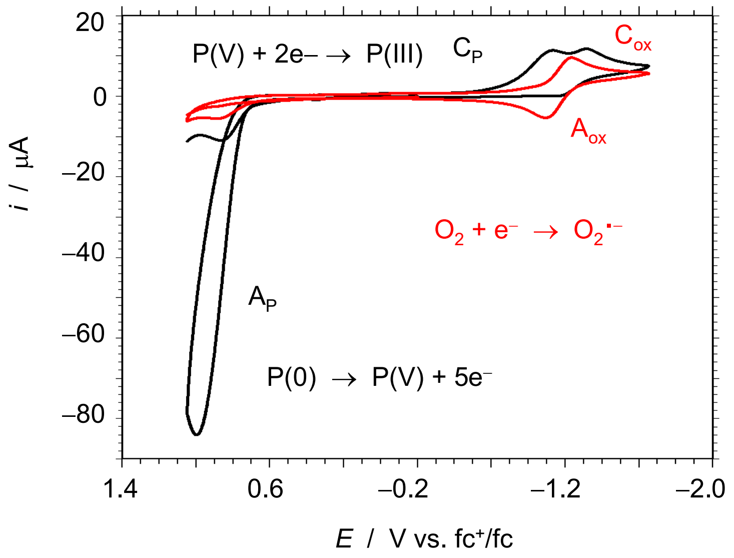

Figure S8 Cyclic voltammograms at glassy carbon electrode of an air–saturated 0.10 M Bu_4NPF_6/DMSO solution before (red) and after (black) addition of catalytic FL–BP. Potential scan rate 50 mV s^{-1}.[14],[15] The response of the original solution is dominated by the characteristic essentially reversible one-electron reduction of dissolved oxygen to radical anion superoxide at ca. −1.2 V vs. fc^+/fc, characterized by the couple cathodic (C_{ox}) and anodic (A_{ox}) peaks. In the voltammogram of the FL-BP containing solution, a prominent apparently irreversible oxidation signal appears at 0.8 V (A_P) which is followed by cathodic signals at −1.0 (C_P) and −1.2 V (C_{ox}), which corresponds to the oxidation of P(0) to P(V) through the process A_P, and the subsequent reduction of this form to possibly any P(III) species via the cathode step C_P.

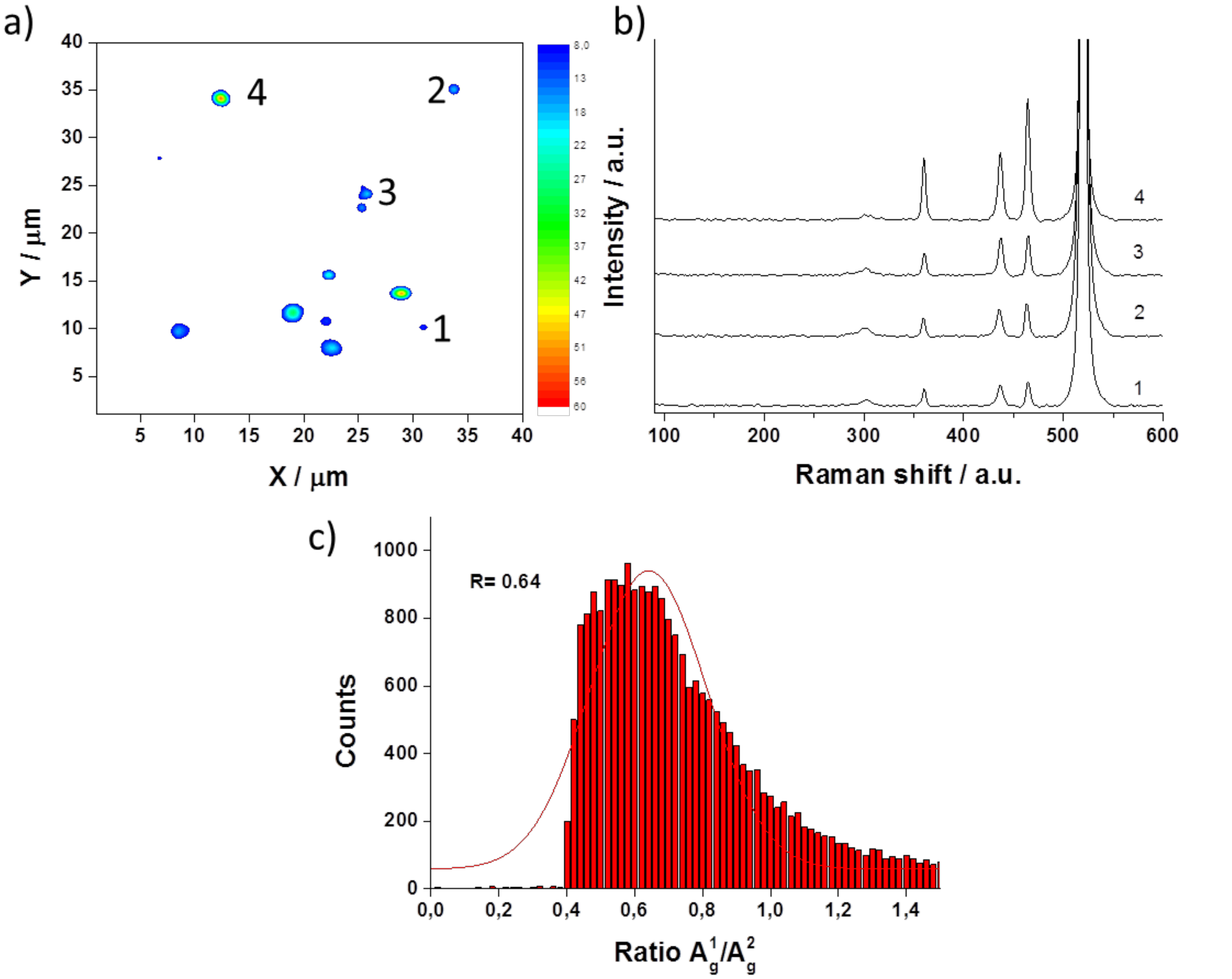

Figure S9. a) SRM and statistical Raman spectroscopy of the FL–BP sample after the reaction with $CBrCl_3$ and 1–Hexene with b) some mean spectra from different points proving that the BP structure is maintained after the reaction. c) Histogram of the A^1_g/A^2_g ratio showing that the material is not oxidized after the reaction. The small particles have a very low signal to noise ratio which leads to heavy tail distributions with values higher than R=1.

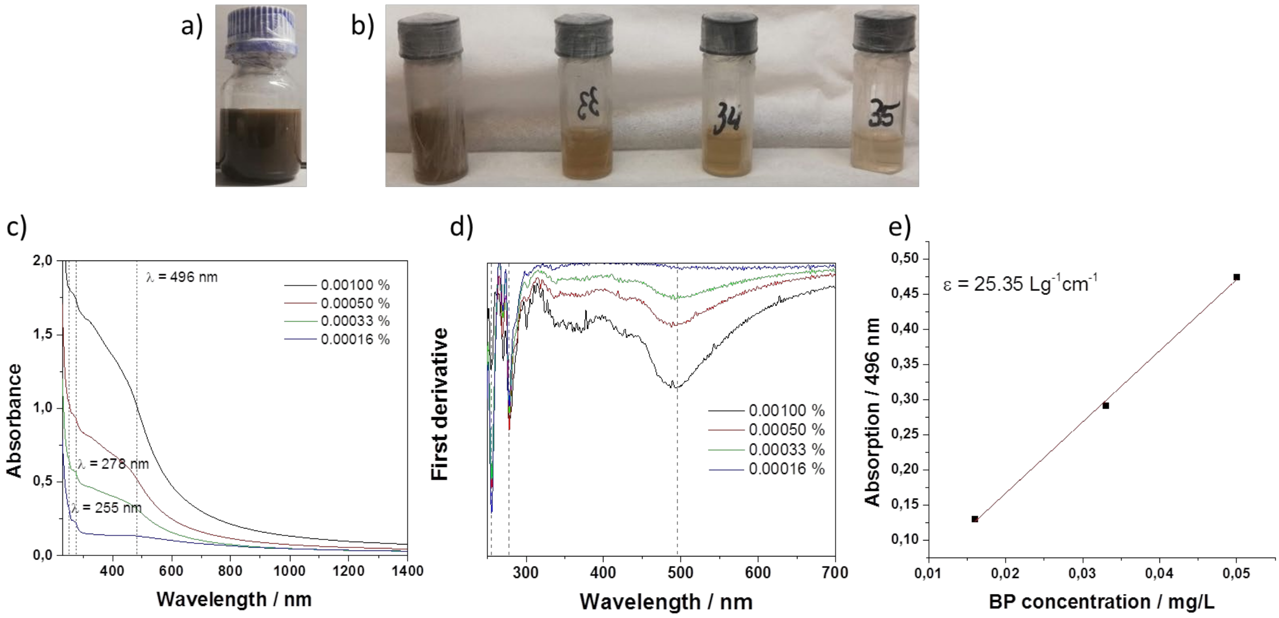

Figure S10: a) Photographs of the BP dispersion obtained in THF after sonication and solvent exchange, and b) the dilution series from more concentrated (left) to less concentrate (right). Figure c) depicts the absorption spectra of the obtained dispersions in THF, showing how the corresponding absorption bands of BP increase with the concentration. In order to better identify the BP bands, the first derivate of the absorption spectra are depicted in Figure d), showing three main bands at 255, 278 and 496 nm. The exact concentration of BP in THF was determined by ICP in order to obtain the extinction coefficient of BP in THF, which is 25.35 $Lg^{-1}cm^{-1}$.

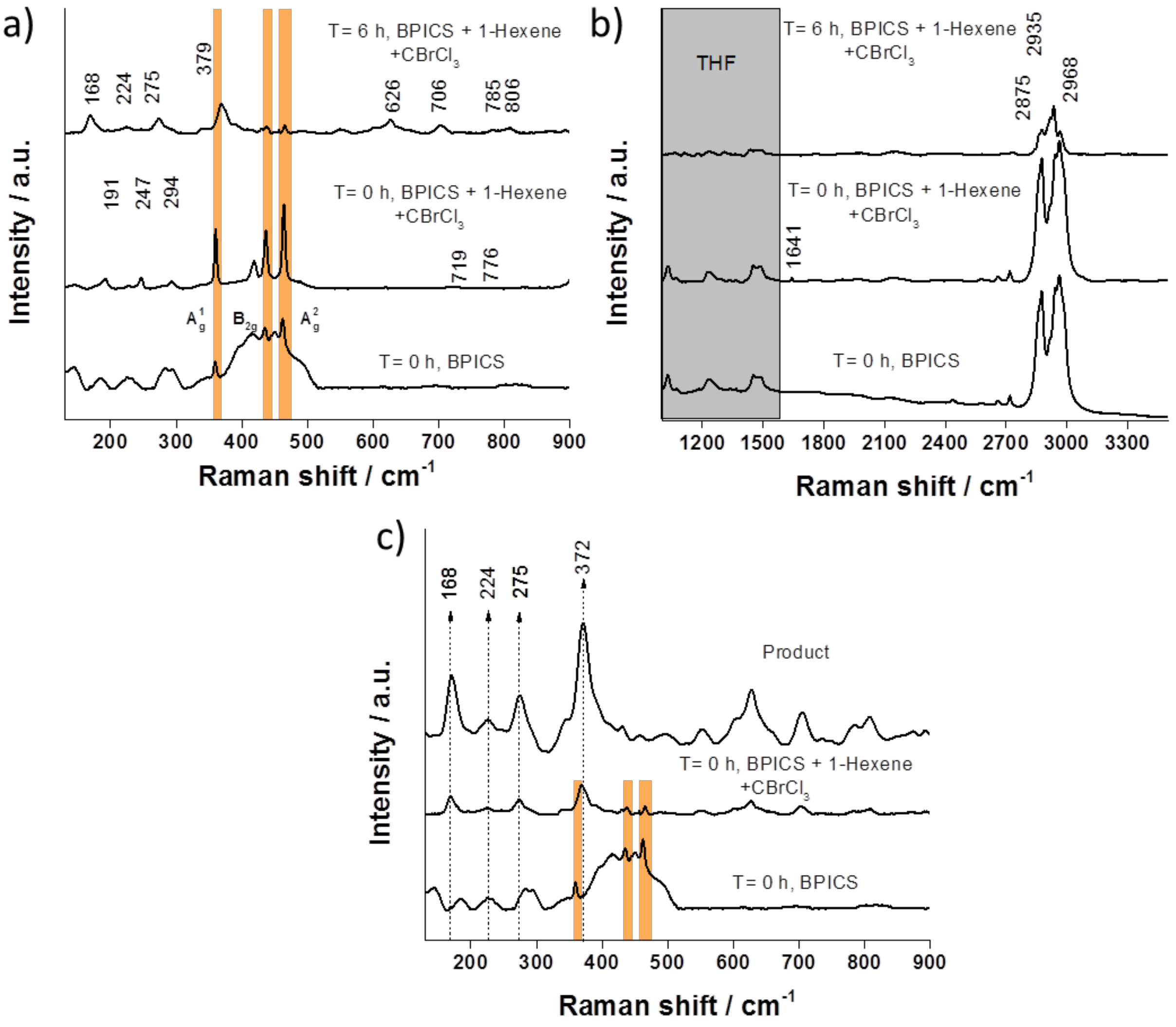

Figure S11. The Raman spectra of a KP_6 dispersion in THF can be measured showing the A^1_g, B_{2g} and A^2_g BP spectroscopic bands at 362, 440 and 466 cm^{-1}, respectively. Furthermore, the spectroscopic bands of the potassium intercalated BP can be observed in the region of 290 and 400 cm^{-1}. Immediately after the addition of $CBrCl_3$ to a BP dispersion containing 1–hexene, the spectroscopic bands corresponding to $CBrCl_3$ are observed at T= 0h. After 6 h, the concentration of the product product 3–bromo–1,1,1–trichloroheptane increases and the new characteristic bands appear at 168, 224, 275 and 379 cm^{-1}. b) The Raman modes at T= 0 and 10 h. The THF modes are stressed in grey and in this regime of the spectra is possible to visualize the corresponding peak of 1–Hexene at 1641 cm^{-1}. After the reaction, the Raman modes corresponding to the product at around 2900 cm^{-1} can be distinguished from the THF ones. c) The difference between BPICS in THF, the reaction mixture and the pure product in which the BP modes are no longer observed.[1a, 16]

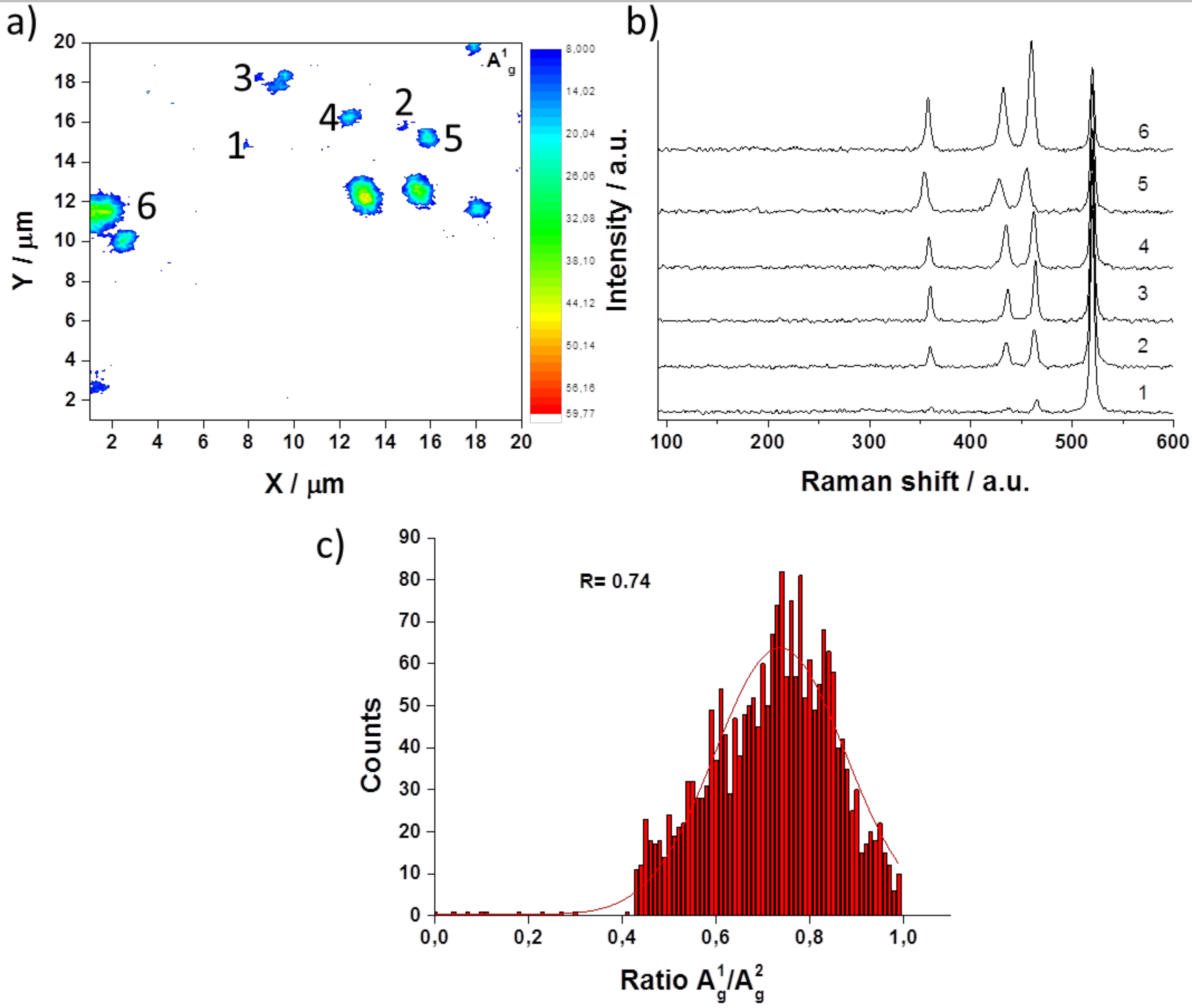

Figure S12. a) SRM and statistical Raman spectroscopy of the FL–BP KP_6 sample after the reaction with $CBrCl_3$ and 1–Hexene with b) single spectra from different points proving that the BP structure is maintained after the reaction. c) Histogram showing the A^1_g/A^2_g ratio, proving that the material is not oxidized after the reaction.

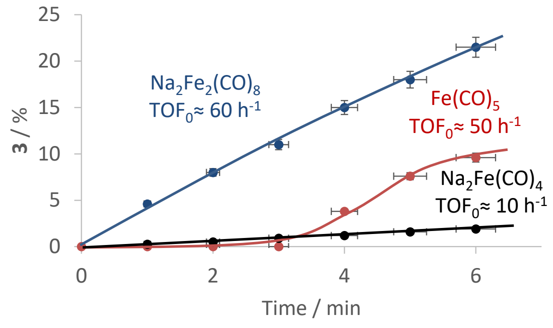

Figure S13. Initial kinetic points of the radical coupling between equimolecular amounts of **1** and **2** in 0.5 M THF solution at 25 ºC with 5 mol% Fe^0 (red curve), Fe^{1-} (blue line) and Fe^{2-} (black line) carbonyl catalysts.[17],[18]

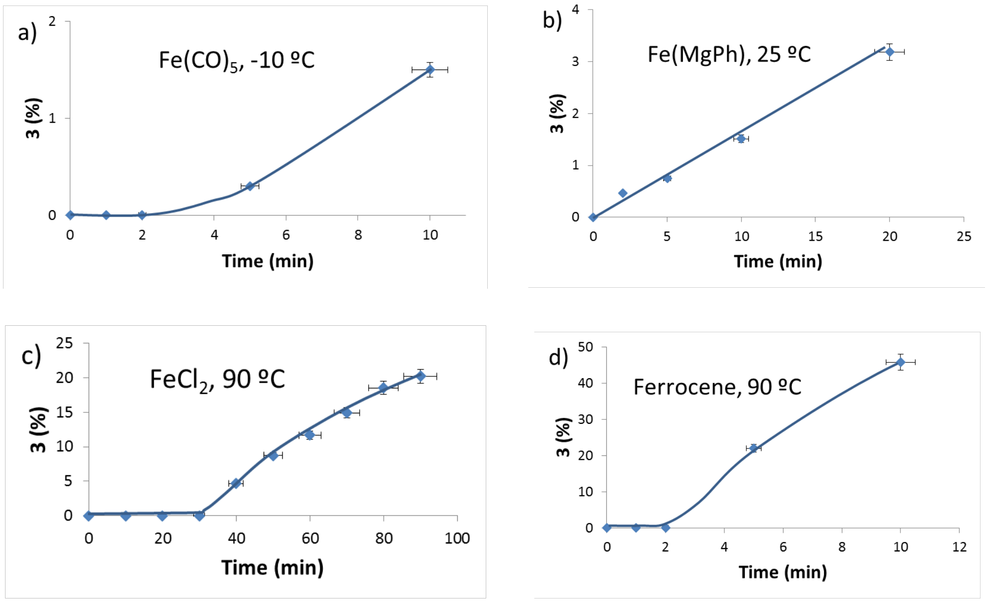

Figure S14 Kinetics of the radical coupling between equimolecular amounts of **1** and **2** in 0.5 M THF solution (low valence Fe) or **2** as a solvent (0.15 M of **1**, Fe cations) with 5 mol% Fe catalyst. Different Fe^{2+} and Fe^{0} compounds present a clear induction time after which the reaction smoothly starts, and $Fe^{2-}(MgPh)_2$ shows high catalytic.

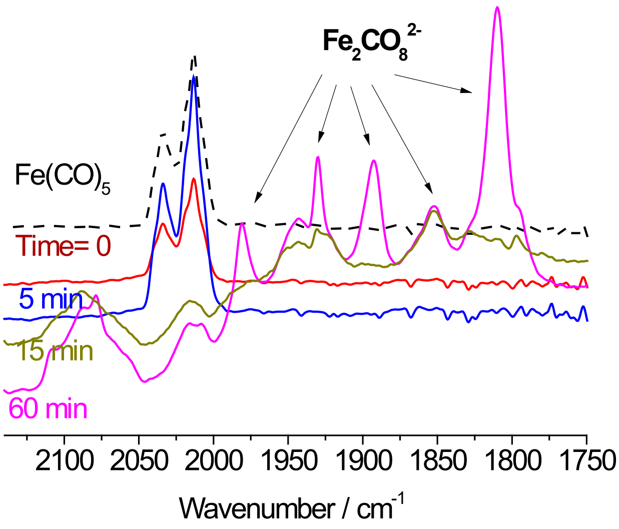

Figure S15 Fourier–transformed infrared spectroscopy (FT–IR) of the radical coupling between **1** and **2** (1:1 mol) in solution with 20 mol% $Fe(CO)_5$ as a catalyst at RT. The CO region of the in–situ Fourier–transformed infrared spectroscopy (FT–IR) of the reaction between equimolecular amounts of **1** and **2** in CH_2Cl_2 solution catalysed by $Fe(CO)_5$ shows the original $Fe(CO)_5$ peaks at 2034 and 2014 cm^{-1} disappearing at expenses of five new electronically richer CO signals at 1980, 1930, 1892, 1852 and 1810 cm^{-1}, which nicely fit the reported signals for $Fe_2CO_8^{2-}$ salts.[18-19]

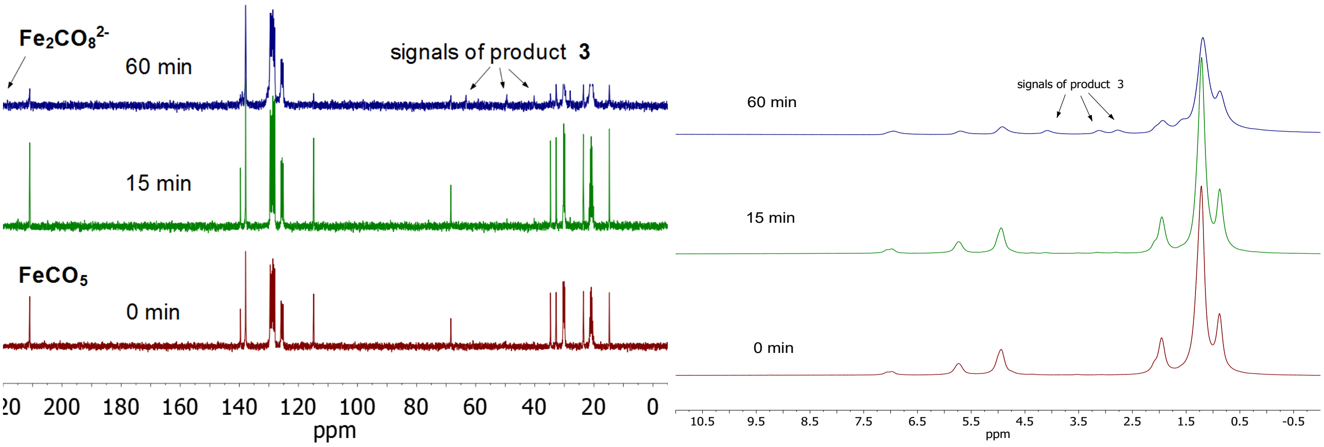

Figure S16 ^{13}C (left) and ^{1}H (right) NMR spectra for the radical coupling between **1** and **2** in toluene–*d*^{8} solution with 20 mol% $Fe(CO)_5$ as a catalyst at room temperature.^{13}C nuclear magnetic resonance (NMR) study in toluene–*d*^{8} solvent (left spectrum) also confirms the progressive disappearance of the $Fe(CO)_5$ signal (at 211.0 ppm) with the concomitant formation of a new signal at 218.7 ppm, assignable to $Fe_2CO_8^{2-}$ species.[S15b,c] Notice that both FT–IR and NMR techniques detect evolution of the CO signals after 5–15 min reaction time, just when product **3** starts to be formed according to the kinetic data obtained by gas–chromatography and the complementary ^{1}H NMR spectra (right spectrum).

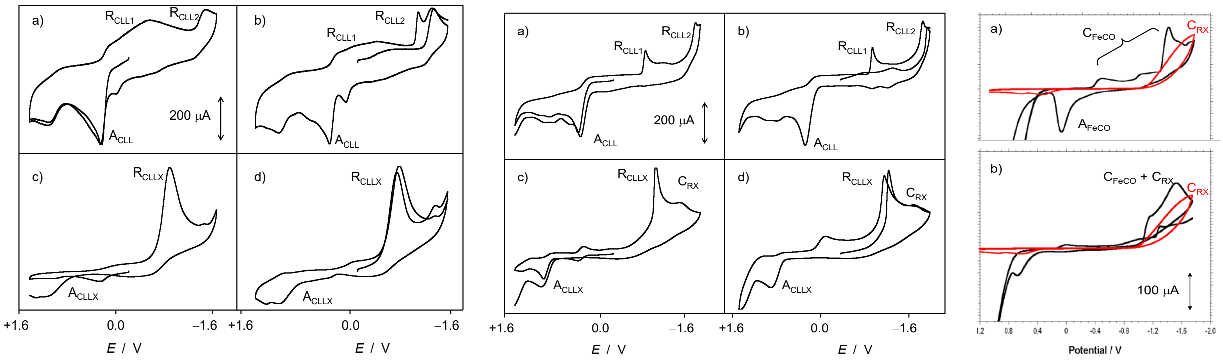

Figure S17 *Left:* Cyclic voltammograms (a,c) positive–going, b,d) negative–going potential scans, at glassy carbon electrode of: a,b) 5 mM $Na_2Fe_2(CO)_8$; c,d) 5 mM $Na_2Fe_2(CO)_8$ plus 20 mM $CBrCl_3$ 2 solutions in 0.10 M Bu_4NPF_6/MeCN. *Middle:* Cyclic voltammograms at glassy carbon electrode of a,b) 5 mM $Na_2Fe(CO)_4$; c,d) 5 mM $Na_2Fe(CO)_4$ plus 20 mM $CBrCl_3$ 2 solutions in 0.10 M Bu_4NPF_6/MeCN. *Right:* Cyclic voltammograms at glassy carbon electrode of a) 5 mM $Fe(CO)_5$ plus 20 mM 2; b) 5 mM $Fe(CO)_5$ plus 20 mM **2** and 1–decene **1** solutions in 0.10 M Bu_4NPF_6/MeCN (black lines), red lines correspond to blank voltammograms at 0.10 M **2** solutions in the same electrolyte. Potentials vs. Pt pseudo–reference electrode and potential scan rate 500 mV s^{-1} in all cases. The voltammetry of $Na_2Fe_2(CO)_8$ in deoxygenated MeCN solutions, before and after the addition of $CBrCl_3$ **2**, shows that, without the halide, an apparently irreversible oxidation peak at +0.35 V (A_{CLL}) appears in the initial anodic scan voltammogram, followed by two reduction peaks (R_{CLL1} and R_{CLL2}) in the subsequent cathodic scan. A_{CLL} is attributed to a rapid oxidation to Fe^0, and R_{CLL1} and R_{CLL2} to the subsequent reductions to Fe^{1-} and Fe^{2-}, respectively. Upon addition of **2**, the signals A_{CLL}, R_{CLL1} and R_{CLL2} are replaced by an intense cathodic wave at –0.95 V (R_{CLLX}) and a main anodic signal at ca. +0.95 V (A_{CLLX}). Blank voltammograms with neat **2** show the absence of these peaks when Fe is not present. R_{CLLX} is catalytically enhanced whereas A_{CLLX} is positively shifted and decreased in height relative to the parent oxidation peak A_{CLL}, which suggests that an electrochemical catalytic cycle $Fe^{2-\text{ or }1-/0}$ might be operating in the presence of **2**. The cyclic voltammograms of Collman´s reagent under exactly the same conditions than $Na_2Fe_2(CO)_8$ show the same prominent catalytic A_{CLLX} and R_{CLLX} signals in the presence of **2**. The addition of 1–decene **1** shows that the redox cycle does continue in the presence of an alkene, since the C_{FECO} and C_{RX} signals persist.

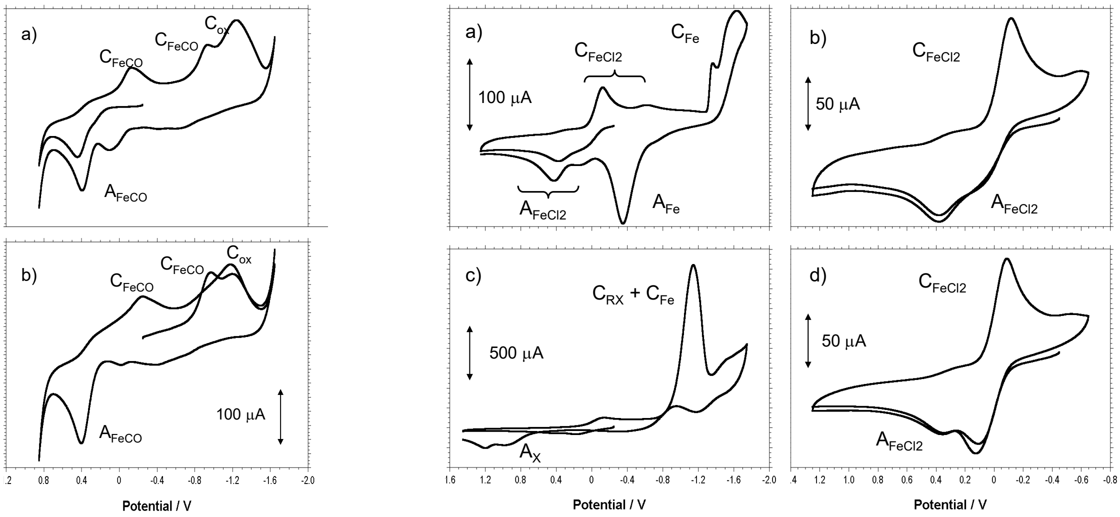

Figure S18 *Left:* Cyclic voltammograms at glassy carbon electrode of 2 mM $Fe(CO)_5$ solutions in air–saturated 0.10 M Bu_4NPF_6/MeCN. a) Positive–going, b) negative–going potential scans. *Right:* Cyclic voltammograms at glassy carbon electrode of a 2 mM $FeCl_2$ solution in 0.10 M Bu_4NPF_6/MeCN a,b) before and c,d) after addition of 20 mM $CBrCl_3$. Potentials vs. Pt pseudo–reference electrode and potential scan rate 500 mV s^{-1} in all cases. Notice the differences in the current scale. Similarly to Collman´s reagent, Fe^{1-} seems also accessible from $Fe(CO)_5$,[20] and the addition of **2** to $FeCl_2$ also provokes the disappearance of the typical reduction (C_{Fe}) and stripping (A_{Fe}) voltammogram peaks, corresponding to the deposit of Fe^0 and its oxidative dissolution, respectively, and the appearance of a prominent cathodic signal at –1.2 V, accompanied by a crossover at ca. –0.9 V in the anodic scan. Moreover, the addition of **1** enhances the cathodic signal at –1.2 V, as it occurs with Fe carbonyls. These features parallel those found for Fe carbonyls and suggest that, again, a $Fe^{2-\text{ or }1-/0}$ redox pair is in operation when starting from $FeCl_2$.

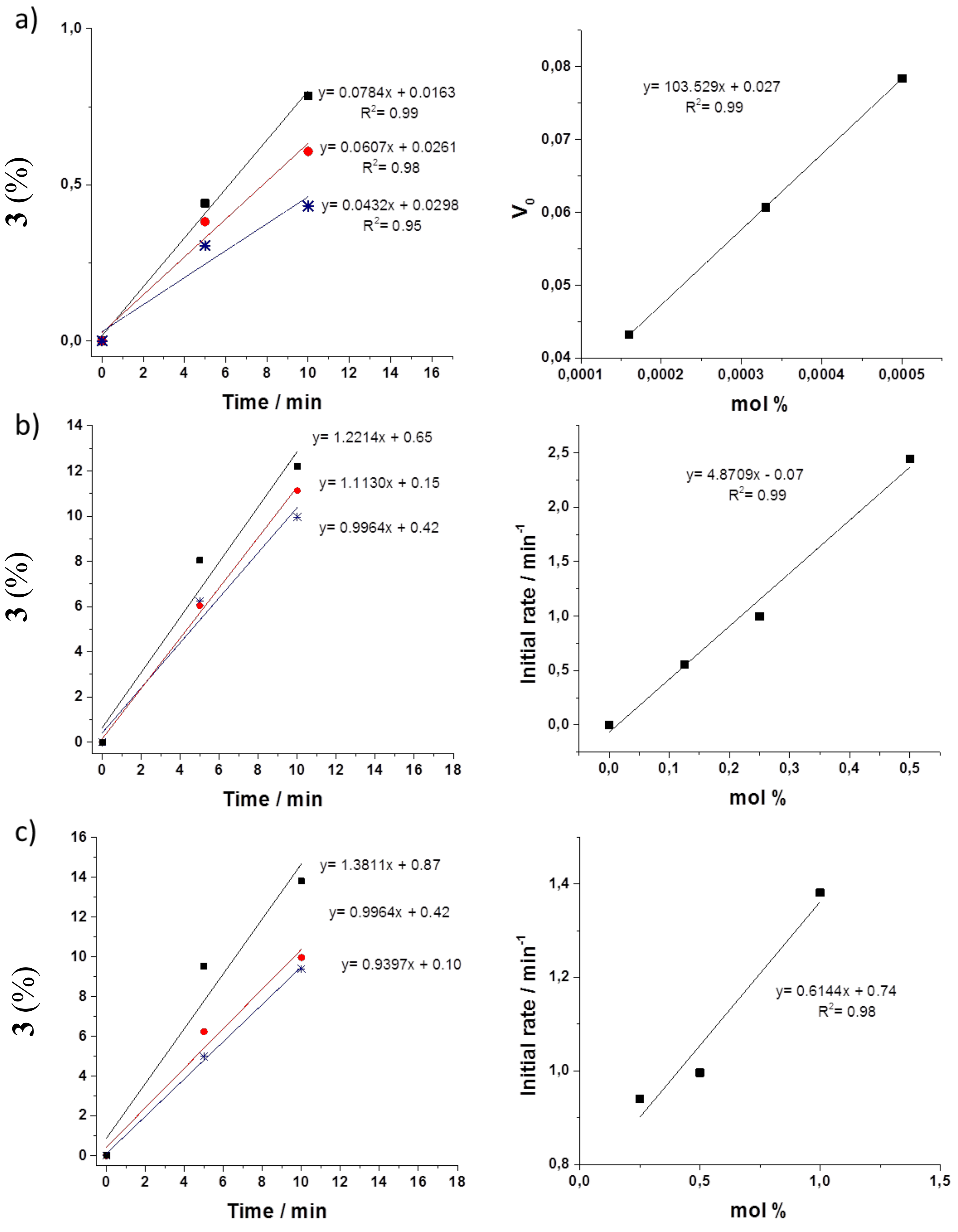

Figure S19. Kinetic studies at different concentrations of FL–BP catalyst (a), 1–decene **1** (b) and $CBrCl_3$ **2** (c) for the radical reaction between equimolecular amounts of **1** and **2** in 0.5 M THF solution at 25 ºC.

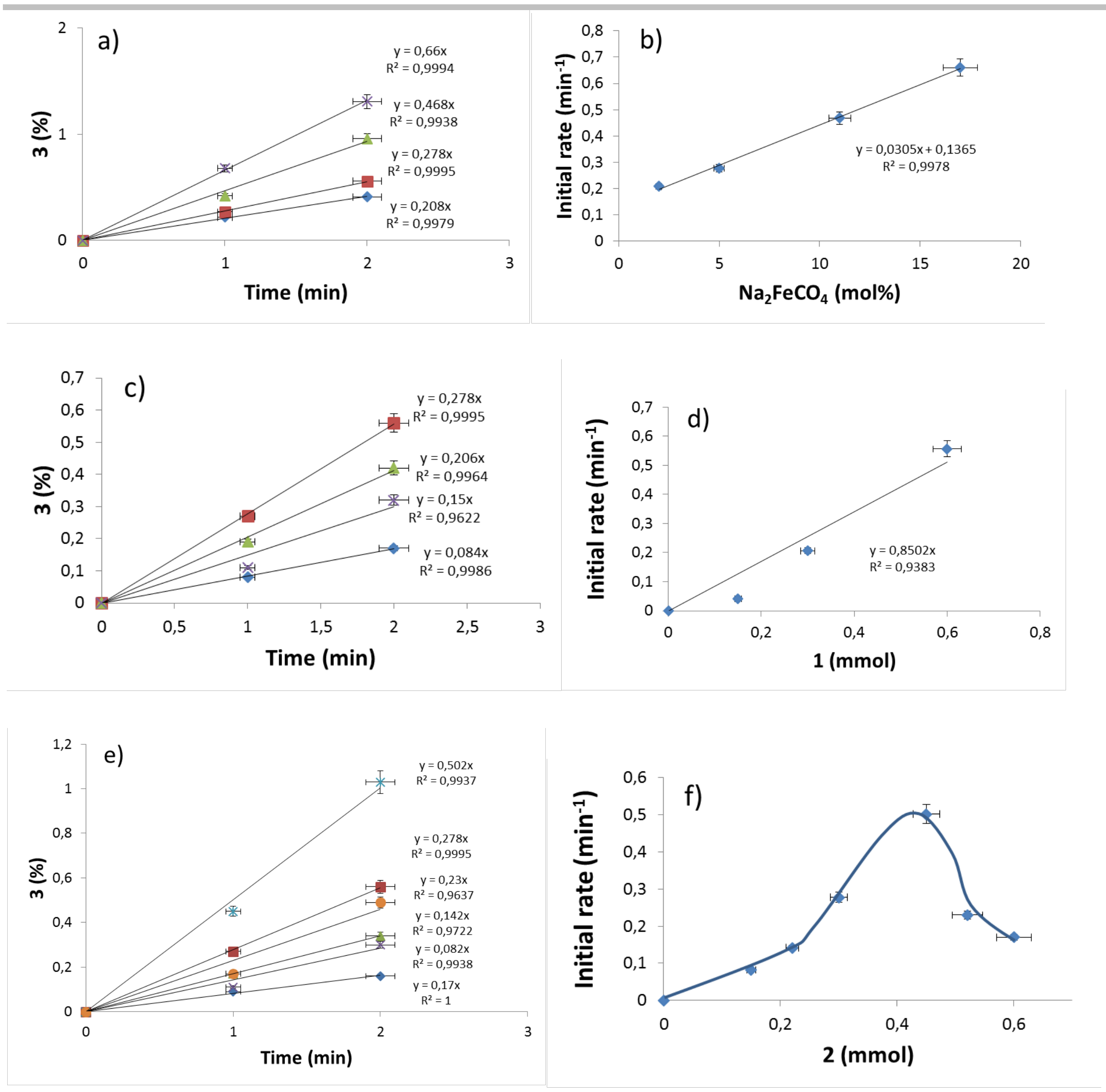

Figure S20. Kinetic studies at different concentrations of $Na_2Fe(CO)_4$ (a–b), 1–decene **1** (c–d) and $CBrCl_3$ **2** (e–f) for the radical reaction between equimolecular amounts of **1** and **2** in 0.5 M THF solution at 25 ºC. The individual kinetics are also shown for the sake of data reliability.

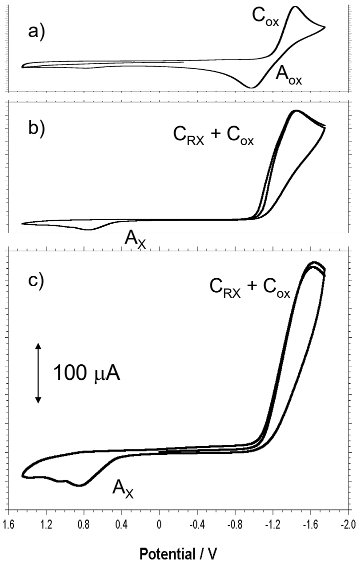

Figure S21. Cyclic voltammograms at glassy carbon electrode for a–c) an air–saturated 0.10 M Bu_4NPF_6/MeCN solution plus b,c) 10 mM **2**. Potentials vs. Pt pseudo–reference electrode. Potential scan rate 500 mV s^{-1}.

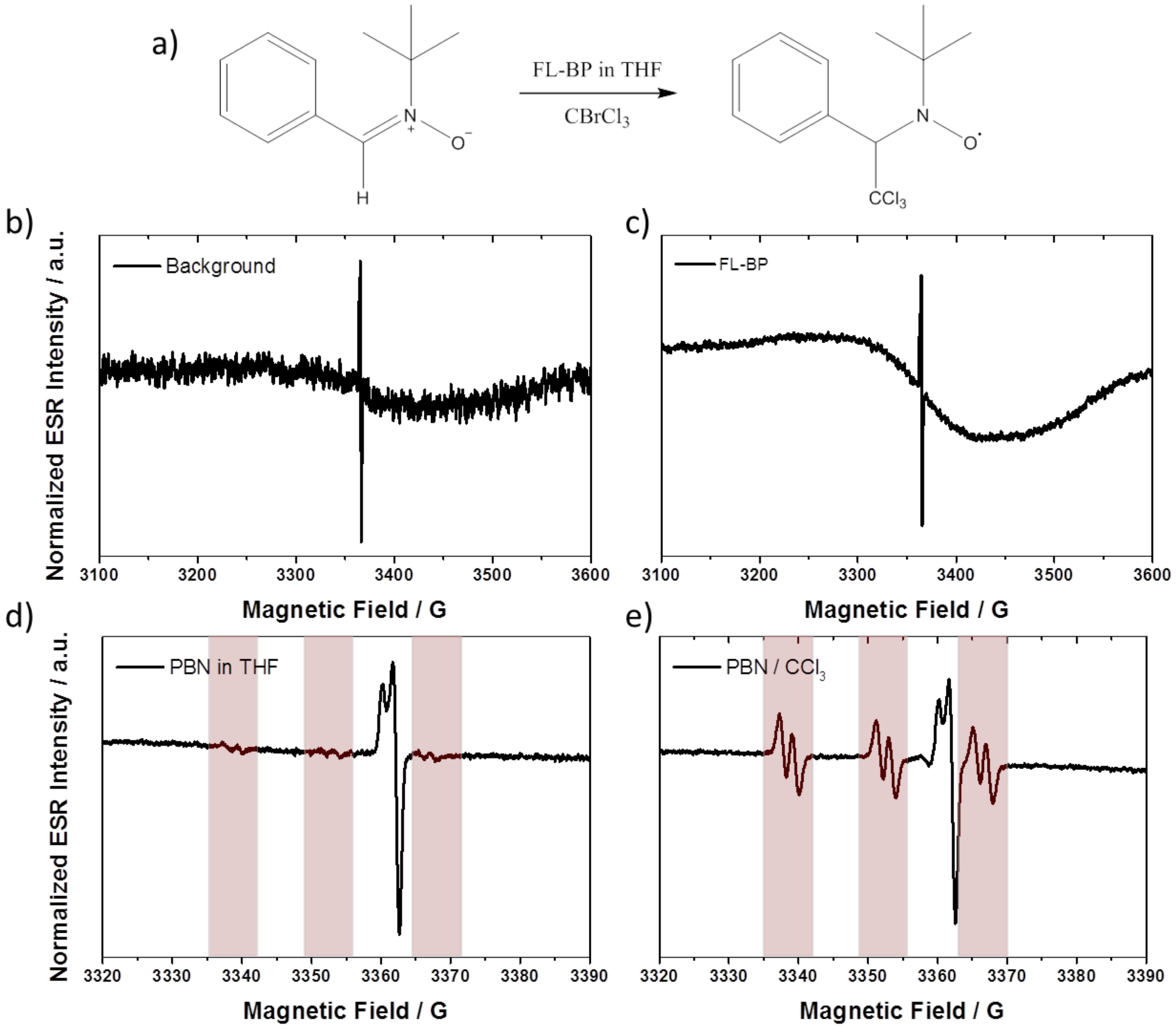

Figure S22. a) Scheme of the reaction between N–*tert*–butyl–α–phenylnitrone (PBN) spin trap and $CBrCl_3$ showing the radical formation. Electron Paramagnetic Resonance of b) an empty quartz vial with a signal at 3362 G, close to g=2, corresponding to impurities (possibly Na or Ca), c) FL–BP in THF in the quartz vial with the fine signal corresponding to the impurities and a wide line coming from BP (as observed in BP bulk solid measurements). This proves that after the exfoliation, FL–BP are obtained without radicals or dandling bonds in the phosphorene sheets. The graphs d) and e) depict the signal of the PBN radical formed after addition of $CBrCl_3$. In the case of PBN please note the characteristic N triplet signal appearing due to a hyperfine coupling of the electron to the nitrogen nuclei. The split of the triplet is caused by H nuclei, which cannot be completely resolved due to the high PBN concentration of the solution. The intensity gain of the PBN signal is a clear signal of the successful reduction of the molecule as it is a well–known spin trap for CCl_3 systems.

Substrate and product characterisation

3–Bromo–1,1,1–trichloroundecane (3):

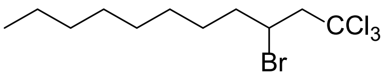

Colourless oil (118.5 mg, 70%); ^{1}H NMR ($CDCl_3$, 300 MHz): δ 4.33 (m, 1H), 3.45 (dd, J = 15.8, 5.1 Hz, 1H), 3.22 (dd, J = 15.8, 5.2 Hz, 1H), 2.13 – 1.84 (m, 2H), 1.66 – 1.45 (m, 2H), 1.34 (dd, J = 31.2, 9.0 Hz, 10H), 0.88 (t, J = 6.7 Hz, 3H). ^{13}C NMR ($CDCl_3$, 300 MHz) δ 97.26 (CCl_3), 62.73 (CH_2), 49.22 (CHBr), 39.59 (CH_2), 31.85 (CH_2), 29.39 (CH_2), 29.22 (CH_2), 28.77 (CH_2), 27.28 (CH_2), 22.67 (CH_2), 14.12 (CH_3). GC/MS (m/z, M^+ 338), major peaks found: 149 (100 %), 185 (32%), 221 (3%), 267 (1%), 338 (<1%). IR (υ, cm^{-1}) 2930 (C–H, vi),, 790 (C–Br, m), 700 (C–Cl_3, m).

(E)–9–Bromo–11,11,11–trichloroundeca–1,5–diene (6):

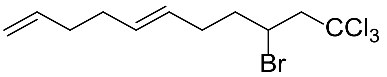

The compound was prepared according to the representative procedure but using 0.4 mmol of alkene. Colourless oil, mixture of isomers (50/50) (107.0 mg, 80%); ^{1}H NMR ($CDCl_3$, 300 MHz) δ 5.98 – 5.64 (m, 1H), 5.63 – 5.27 (m, 2H), 5.18 – 4.86 (m, 2H), 4.49 – 4.21 (m, 1H), 3.61 – 3.36 (m, 1H), 3.34 – 3.11 (m, 1H), 2.56 – 1.75 (m, 8H). ^{13}C NMR ($CDCl_3$, 300 MHz) δ 138.38 (CH), 131.56 (CH), 127.92 (CH), 114.94 (CH_2), 97.29 (CCl_3), 62.92 (CH_2), 48.70 (CHBr), 39.46 (CH_2), 33.84 (CH_2), 31.25 (CH_2), 26.18 (CH_2). GC/MS (m/z, M^+ 334), major peaks found: 67 (100 %), 109 (51%), 139 (50%), 175 (29%), 221 (6%), 293 (2%), 335 (<1%). IR (υ, cm^{-1}) 2925 (C–H, l), 1460 (C=C, l), 913 (C–H, i), 774 (C–Br, i), 704 (C–Cl_3, m).

(3–Bromo–5,5,5–trichloropentyl)benzene (7):

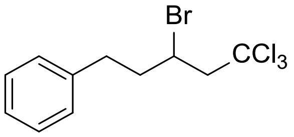

Colourless oil (123.9 mg, 75%); ^{1}H NMR ($CDCl_3$, 300 MHz) δ 7.43 – 7.12 (m, 5H), 4.32 (m, 1H), 3.53 (dd, J = 15.8, 4.9 Hz, 1H), 3.28 (dd, J = 15.8, 5.7 Hz, 1H), 2.99 (m, 1H), 2.83 (m, 1H), 2.42 (m, 1H), 2.27 (m, 1H). ^{13}C NMR ($CDCl_3$, 300 MHz) δ 140.31 (C), 128.73 (2xCH), 128.66 (2xCH), 126.48 (CH), 97.16 (CCl_3), 62.83 (CH_2), 48.57(CHBr), 41.10 (CH_2), 33.67 (CH_2). GC/MS (m/z, M^+ 330), major peaks found: 91 (100 %), 117 (10%), 141 (3%), 177 (5%), 213 (1%), 330 (10%). IR (υ, cm^{-1}) 2980 (vi, C–H ar), 2920 (vi, C–H ar), 1720 (I, C–H).

4–(2–Bromo–4,4,4–trichlorobutan–2–yl)–1–methylcyclohex–1–ene (8):

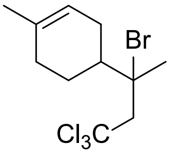

The compound was prepared according to the representative procedure but using 0.4 mmol of alkene. Colourless oil, mixture of isomers (50/50) (120.4 mg, 90%); ^{1}H NMR ($CDCl_3$, 300 MHz) δ 5.48 – 5.28 (m, 1H), 3.88 – 3.38 (m, 2H), 2.40 – 1.81 (m, 9H), 1.67 (s, 3H), 1.62 – 1.39 (m, 1H). ^{13}C NMR ($CDCl_3$, 300 MHz) δ 133.78 (C), 119.27 (CH), 96.01 (CCl_3), 73.29 (CBr), 63.00 (CH_2), 45.20 (CH), 30.16 (CH_2), 29.51 (CH_3), 28.00 (CH_2), 25.82 (CH_2), 22.90 (CH_3). GC/MS (m/z, M^+ 334), major peaks found: 93 (100 %), 121 (91%), 145 (14%), 219 (12%), 253 (18%), 334 (4%). IR (υ, cm^{-1}) 2920 (l, C–H), 913 (i, C–H), 740 (i, C–Cl).

5–(2–Bromo–4,4,4–trichlorobutan–2–yl)–2–methylcyclohex–2–en–1–one (9):

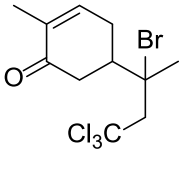

The compound was prepared according to the representative procedure but using 0.4 mmol of alkene. Colourless oil, mixture of isomers (80/20), (76.9 mg, 55%); ^{1}H NMR ($CDCl_3$, 300 MHz) δ 5.60 (s, 1H), 3.66 – 3.58 (m, 1H), 3.14 – 2.86 (m, 2H), 2.66 – 2.48 (m, 2H), 2.22 (s, 3H), 1.82 (s, 2H), 1.42 (d, 2H). ^{13}C NMR ($CDCl_3$, 300 MHz) δ 199.03 (CO), 144.09 (CH), 135.55 (C), 95.72 (CCl_3), 71.89 (CBr), 62.37 (CH_2), 45.76 (CH), 41.31 (CH_2), 29.41 (CH_2), 23.65 (CH_3), 15.58 (CH_3). GC/MS (m/z, M^+ 348), major peaks found: 109 (100 %), 135 (22%), 171 (7%), 207 (40%), 267 (31%), 348 (<1%). IR (υ, cm^{-1}) 2960 (m, C–H), 2920 (m, C–H), 1660 (vi, C=O), 780 (i, C–Br), 715 (i, C–Cl).

1–Bromo–1–(2,2,2–trichloroethyl)cyclohexane (10):

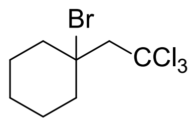

Colourless oil (95.7 mg, 65%); ^{1}H NMR (CDCl$_3$, 300 MHz) δ 3.63 (s, 2H), 2.23 (d, *J* = 12.9 Hz, 2H), 2.05 – 1.64 (m, 8H). ^{13}C NMR (CDCl$_3$, 300 MHz) δ 96.14 (CCl$_3$), 72.52 (CBr), 68.07 (CH$_2$), 40.56 (2xCH$_2$), 25.05 (2xCH$_2$), 23.29 (CH$_2$). GC/MS (m/z, M$^+$ 294), major peaks found: 81 (100 %), 117 (32%), 141 (14%), 177 (27%), 213 (1%), 294 (<1%). IR (υ, cm^{-1}) 2936 (vi, C–H), 2854 (m, C–H), 1445 (m, C–H), 943 (m, C–Br), 701 (C–Cl).

((4–Bromo–6,6,6–trichlorohexyl)oxy)(tert–butyl)diphenylsilane (11):

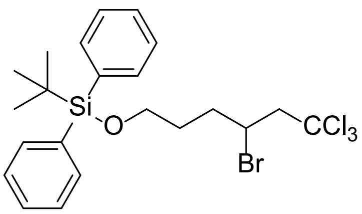

The compound was prepared according to the representative procedure but using 4 mmol of **2**. Colourless oil (313.7 mg, 60%); ^{1}H NMR (CDCl$_3$, 300 MHz) δ 7.79 – 7.52 (m, 4H), 7.55 – 7.32 (m, 6H), 4.39 (td, *J* = 9.1, 5.1 Hz, 1H), 3.88 – 3.57 (m, 2H), 3.48 (dd, *J* = 15.8, 4.9 Hz, 1H), 3.23 (dd, *J* = 15.8, 5.5 Hz, 1H), 2.36 – 2.16 (m, 1H), 2.12 – 1.93 (m, 1H), 1.90 – 1.64 (m, 2H), 1.06 (s, 9H). ^{13}C NMR (CDCl$_3$, 300 MHz) δ 135.72 (4xCH), 133.92 (2xC–Si), 129.82 (2xCH), 127.83 (4xCH), 97.30 (CCl$_3$), 62.99 (CH$_2$), 62.93 (CH$_2$), 49.31 (CHBr), 36.29 (CH$_2$), 30.51 (CH$_2$), 27.03 (CH$_3$), 19.36 (C). GC/MS (m/z, M$^+$ 522), major peaks found: 217 (70%), 263 (100%), 293 (22%), 429 (3%), 465 (11%), 522 (<1%). IR (υ, cm^{-1}) 3070 (m, C–O), 2929 (vi, C–H), 2856 (i, C–H), 1111 (vi, Si–O), 700 (i, C–Cl), 504 (i, Si–O).

1–(2–Bromo–4,4,4–trichlorobutoxy)–2–iodobenzene (12):

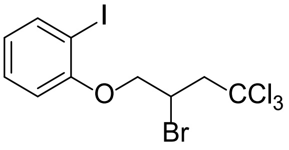

Colourless oil (109.4 mg, 60%); ^{1}H NMR (CDCl$_3$, 300 MHz) δ 7.80 (dd, *J* = 7.8, 1.6 Hz, 1H), 7.35 – 7.27 (m, 1H), 6.78 (ddd, *J* = 15.0, 7.8, 1.2 Hz, 2H), 4.71 – 4.55 (m, 1H), 4.47 (dd, *J* = 10.2, 4.6 Hz, 1H), 4.28 (dd, *J* = 10.2, 6.6 Hz, 1H), 3.82 (dt, *J* = 16.0, 5.5 Hz, 1H), 3.41 (dd, *J* = 15.9, 6.3 Hz, 1H). ^{13}C NMR (CDCl$_3$, 300 MHz) δ 156.54 (C–O), 139.98 (CH), 129.69 (CH), 123.77 (CH), 112.72 (CH), 97.08 (CCl$_3$), 86.79 (CI), 72.17 (CH$_2$), 58.94 (CH$_2$), 43.19 (CHBr). GC/MS (m/z, M$^+$ 458) major peaks found: 203 (10%), 220 (100%), 423 (2%), 458 (10%). IR (υ, cm^{-1}) 2929 (l, C–H), 1111 (l, aromatic), 913 (i, C–Br), 743 (i, C–Cl$_3$).

(3R,8R,9R,10S,13S,14R,17S)–10,13–Dimethyl–17–((S)–6–methylheptan–2–yl)–2,3,4,7, 8,9,10,11,12,13,14,15,16,17–tetradecahydro–1H–cyclopenta[a]phenanthren–3–yl 5–bromo–7,7,7–trichloroheptanoate (13):

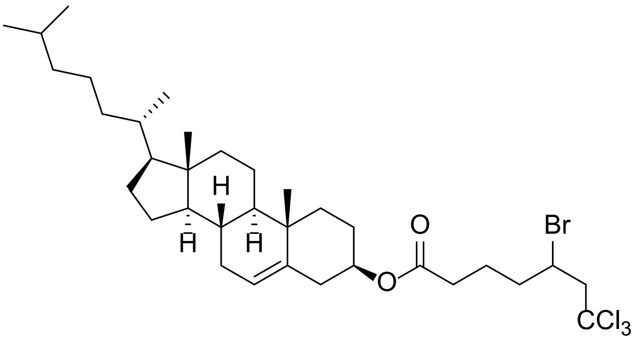

Colourless oil (85%); ^{1}H NMR (CDCl$_3$, 300 MHz) δ 5.38 (d, *J* = 4.3 Hz, 1H), 4.62 (ddd, *J* = 11.6, 10.4, 4.2 Hz, 1H), 4.33 (td, *J* = 9.2, 5.0 Hz, 1H), 3.47 (dd, *J* = 15.8, 4.9 Hz, 1H), 3.23 (dd, *J* = 15.8, 5.4 Hz, 1H), 2.43 – 2.25 (m, 3H), 2.13 (ddd, *J* = 13.3, 10.2, 5.3 Hz, 1H), 2.05 – 1.93 (m, 3H), 1.93 – 1.70 (m, 4H), 1.65 – 1.45 (m, 9H), 1.38 – 1.29 (m, 3H), 1.25 (t, *J* = 10.9 Hz, 4H), 1.19 – 1.06 (m, 6H), 1.02 (s, 4H), 0.91 (d, *J* = 6.5 Hz, 3H), 0.86 (dd, *J* = 6.6, 1.2 Hz, 6H), 0.68 (s, 3H). ^{13}C NMR (CDCl$_3$, 300 MHz) δ 172.48 (C=O) 139.74 (C), 122.90 (CH), 97.22 (CCl$_3$), 74.30 (CH–O),

62.74 (CH_2), 56.86 (CH), 56.32 (CH), 50.21 (CH), 48.42 (CH–Br), 43.58 (CH), 42.49 (C), 39.91 (CH_2), 39.69 (CH_2), 38.83 (CH_2), 38.33 (CH_2), 37.15 (CH_2), 36.77 (C), 36.36 (CH_2), 35.95 (CH), 33.72 (CH_2), 32.04 (CH_2), 29.85 (CH), 28.38 (CH_2), 28.17 (CH_3), 27.99 (CH_2), 24.44 (CH_2), 23.99 (CH_2), 22.96 (CH_2), 22.71 (2xCH_3), 21.20 (CH_2), 19.47 (CH_3), 18.88 (CH_3), 12.02 (CH_3). IR (υ, cm^{-1}) 2968 (vi, C–H), 1725 (i, C=O), 700 (m, C–Cl).

(8S,9R,13R,14R)–3–((5–Bromo–7,7,7–trichloroheptyl)oxy)–13–methyl–6,7,8,9,11,12, 13,14,15,16–decahydro–17H–cyclopenta[a]phenanthren–17–one (14):

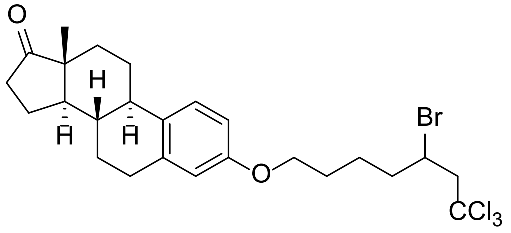

Colourless oil (70%); ^{1}H NMR ($CDCl_3$, 300 MHz) δ 7.20 (d, J = 8.6 Hz, 1H), 6.71 (dd, J = 8.5, 2.7 Hz, 1H), 6.64 (d, J = 2.4 Hz, 1H), 4.35 (td, J = 9.4, 5.1 Hz, 1H), 3.98 (dd, J = 16.8, 11.1 Hz, 2H), 3.47 (dd, J = 15.8, 5.0 Hz, 1H), 3.24 (dd, J = 15.8, 5.3 Hz, 1H), 3.00 – 2.80 (m, 2H), 2.50 (dd, J = 18.2, 8.5 Hz, 1H), 2.38 (t, J = 8.6 Hz, 1H), 2.32 – 2.20 (m, 1H), 2.13 (dd, J = 17.8, 7.7 Hz, 2H), 2.06 – 1.90 (m, 3H), 1.90 – 1.71 (m, 3H), 1.67 – 1.45 (m, 13H). ^{13}C NMR ($CDCl_3$, 300 MHz) δ 221.04 (C=O), 157.13 (C–O), 137.93 (C), 132.27 (C), 126.49 (CH), 114.77 (CH), 112.28 (CH), 97.29 (CCl_3), 67.61 (CH_2), 62.79 (CH_2), 50.61 (CH), 48.95 (CH), 48.17 (C), 44.16 (CH), 39.35 (CH_2), 38.56 (CH), 36.02 (CH_2), 31.76 (CH_2), 29.81 (CH_2), 28.64 (CH_2), 26.72 (CH_2), 26.09 (CH_2), 24.24 (CH_2), 21.75 (CH_2), 14.02 (CH_3). IR (υ, cm^{-1}) 2926 (vi, C–H), 1256 (l, aromatic), 1738 (i, C=O), 703 (m, C–Cl).

3–Chloro–1,1,1–trifluoroundecane (15):

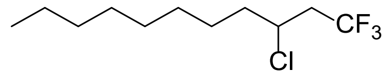

Colourless oil (127.5 mg, 56%); ^{1}H NMR ($CDCl_3$, 300 MHz) δ 4.22 – 4.02 (m, 1H), 2.73 – 2.41 (m, 2H), 1.92 – 1.67 (m, 2H), 1.61 – 1.16 (m, 12H), 0.89 (t, J = 6.5 Hz, 3H). ^{13}C NMR ($CDCl_3$, 300 MHz) δ 125.31 (q, J = 277.6 Hz, CF_3), 54.16 (q, J = 3.2 Hz, CHCl), 42.45 (q, J = 28.4 Hz, CH_2), 38.08 (CH_2), 31.79 (CH_2), 29.33 (CH_2), 29.14 (CH_2), 28.84 (CH_2), 25.89 (CH_2), 22.61 (CH_2), 14.03 (CH_3). ^{19}F NMR ($CDCl_3$, 300 MHz) δ –63.85 (t, J = 10.3 Hz). GC/MS (m/z, M^+ 244), major peaks found: 98 (11%), 137 (27%), 173 (100 %), 208 (1%), 244 (<1%). IR (υ, cm^{-1}) 2928 (vi, C–H), 2857 (vi, C–H), 1262 (I, C–F), 1150 (I, C–F).

7–Chloro–8–(trifluoromethyl)tetradecane (16):

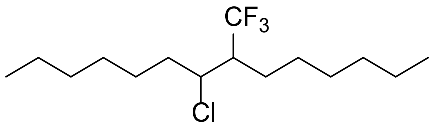

Colourless oil (187.5 mg, 67%); ^{1}H NMR ($CDCl_3$, 300 MHz) δ 4.26 – 4.09 (m, 1H), 2.44 – 2.22 (m, 1H), 1.90 – 1.16 (m, 20H), 0.97 – 0.82 (t, 6H). ^{13}C NMR ($CDCl_3$, 300 MHz) δ , 127.15 (q, J = 281.6 Hz, CF_3) 59.27 (q, CH), 48.54 (d, J = 24.6 Hz, CHCl), 36.45 (CH_2), 31.58 (CH_2), 31.48 (CH_2), 29.30 (CH_2), 28.54 (CH_2), 27.96 (CH_2), 26.86 (CH_2), 24.57 (CH_2), 22.54 (CH_2), 22.51 (CH_2), 13.98 (2xCH_3). ^{19}F NMR ($CDCl_3$, 300 MHz) δ –67.34 (d, J = 9.2 Hz). GC/MS (m/z, M^+ 300), major peaks found: 43 (100 %), 70 (71%), 97 (34%), 165 (21%), 193 (5%), 264 (9%), 298 (<1%). IR (υ, cm^{-1}) 2929 (vi, C–H), 2859 (i, C–H), 1250 (i, C–F), 1167 (i, C–F), 749 (m, C–Cl).

(3–Chloro–5,5,5–trifluoropentyl)benzene (17):

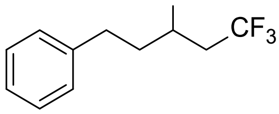

Colourless oil (167.2 mg, 76%); ^{1}H NMR ($CDCl_3$, 300 MHz) δ 7.43 – 7.17 (m, 5H), 4.24 – 4.02 (m, 1H), 3.08 – 2.78 (m, 2H), 2.75 – 2.49 (m, 2H), 2.32 – 1.93 (m, 2H). ^{13}C NMR ($CDCl_3$, 75 MHz) δ 140.31 (C), 128.78 (2xCH), 128.62 (2xCH), 126.51 (CH), 125.74 (q, CF_3), 53.51 (q, CHCl), 42.64 (q, J = 28.5 Hz, CH_2), 39.78 (CH_2), 29.87 (CH_2). ^{19}F NMR ($CDCl_3$, 282 MHz) δ –63.66 (t, J = 17.7, 11.0 Hz). GC/MS (m/z, M^+ 236), major peaks found: 91 (100 %), 106 (9%), 117 (9%), 200 (2%), 236 (24%). IR (υ, cm^{-1}) 2926 (i, C–H), 2855 (m, C–H), 1266 (1, C–F), 1145 (i, C–F), 700 (m, C–Cl).

Methyl 10–chloro–9–(trifluoromethyl)octadecanoate (18):

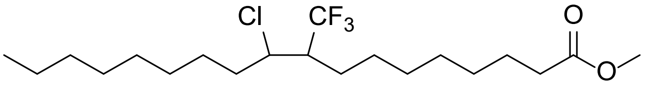

Colourless oil, mixture of isomers; ^{1}H NMR ($CDCl_3$, 300 MHz) δ 4.25 – 4.01 (m, 2H), 3.66 (s, 3H), 2.29 (q, *J* = 7.5 Hz, 2H), 2.11 – 1.89 (m, 2H), 1.84 – 1.53 (m, 4H), 1.49 – 1.04 (m, 20H), 0.88 (t, *J* = 6.5 Hz, 3H). ^{13}C NMR ($CDCl_3$, 300 MHz) δ 174.38 (C=O), 127.15 (q, CF_3), 60.50 (CH_2–C=O), 51.55 (CHBr), 49.08 (q, CH–CF_3), 36.60 (CH_2), 34.22 (CH_2), 32.01 (CH_2), 29.81 (CH_2), 29.64 (CH_2), 29.47(CH_2), 29.38 (CH_2), 29.09(CH_2), 28.82 (CH_2), 27.57 (CH_2, ddd, *J* = 27.7, 21.8, 4.3 Hz), 25.06 (CH_2), 24, 60 (CH_2), 22.78 (CH_2), 21.14 (CH_3–O), 14.20 (CH_3). ^{19}F NMR ($CDCl_3$, 300 MHz) δ –65.57 (dd, *J* = 9.3, 2.9 Hz), –67.33 (d, *J* = 9.1 Hz). GC/MS (m/z, M^+ 400), major peaks found: 74 (100 %), 88 (40%), 97 (6%), 143 (4%), 333 (10%), 400 (1%). IR (υ, cm^{-1}) 2927 (vi, C–H), 2856 (i, C–H), 1742 (vi, C=O), 1256 (m, C–F), 1167 (m, C–F). HRMS (ESI) [M+H^+; calculated for $C_{20}H_{37}ClF_3O_2$: 401.2429] found m/z 401.2434.

Tert–butyl(pent–4–en–1–yloxy)diphenylsilane (S1):

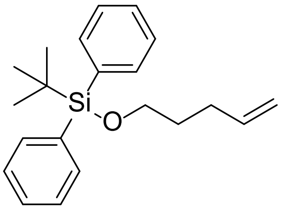

White solid (1.43 mg, 88%); ^{1}H NMR ($CDCl_3$, 300 MHz) δ 7.71 (ddd, *J* = 12.0, 6.6, 4.8 Hz, 4H), 7.50 – 7.36 (m, 6H), 5.84 (ddt, *J* = 16.9, 10.1, 6.6 Hz, 1H), 5.25 – 4.73 (m, 2H), 3.71 (t, *J* = 6.4 Hz, 2H), 2.18 (dt, *J* = 6.6, 4.2 Hz, 2H), 1.84 – 1.60 (m, 2H), 1.09 (s, 9H). ^{13}C NMR ($CDCl_3$, 300 MHz) δ 138.69 (CH), 135.73 (4xCH), 134.26 (2xC–Si), 129.67 (2xCH), 127.74 (4xCH), 114.67 (CH_2–O), 63.45 (CH_2), 32.00 (CH_2), 30.21 (CH_2), 27.03 (3xCH_3), 19.39 (C). GC/MS (m/z, M^+ 324) major peaks found: 199 (81%), 225 (28%), 267 (100%), 324 (<1%). IR (υ, cm^{-1}) 3071 (m, C–O), 2931 (vi, C–H), 2857 (i, C–H), 1428 (i, C–O), 1111 (vi, Si–O), 701 (i, aromatic).

1–(Allyloxy)–2–iodobenzene (S2):

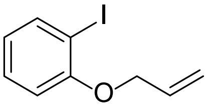

Yellow oil (1.24 g, 95%); ^{1}H NMR ($CDCl_3$, 300 MHz) δ 7.79 – 7.50 (m, 1H), 7.36 – 6.96 (m, 1H), 6.69 (d, *J* = 8.2 Hz, 1H), 6.65 – 6.55 (m, 1H), 6.16 – 5.78 (m, 1H), 5.49 – 5.36 (m, 1H), 5.20 (ddd, *J* = 10.6, 1.4, 0.8 Hz, 1H), 4.77 – 4.31 (m, 2H). ^{13}C NMR ($CDCl_3$, 300 MHz) δ 157.15 (C–O), 139.56 (CH), 132.64 (CH), 129.44 (CH), 122.73 (CH), 117.64 (CH_2), 112.58 (CH), 86.77 (C–I), 69.70 (CH_2–O). GC/MS (m/z, M^+ 260) major peaks found: 165 (4%), 191 (17%), 220 (22%), 260 (100%). IR (υ, cm^{-1}) 1471 (i, C–H), 1275 (i, C=C), 1017 (m, aromatic).

(3S,8S,9S,10R,13R,14S,17R)–10,13–Dimethyl–17–((R)–6–methylheptan–2–yl)–2,3,4,7, 8,9,10,11,12,13,14,15,16,17–tetradecahydro–1H–cyclopenta[a]phenanthren–3–yl hex–5–enoate (S3):

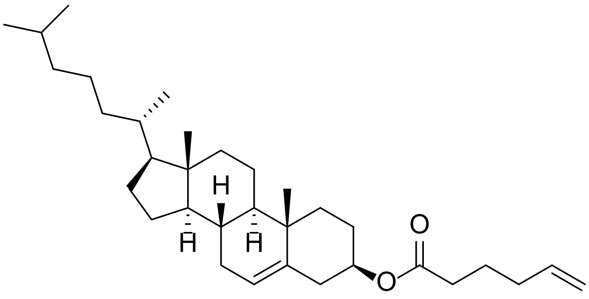

Yellowish solid (1.84 g, 76%); ^{1}H NMR ($CDCl_3$, 300 MHz) δ 5.78 (ddt, *J* = 16.9, 10.1, 6.7 Hz, 1H), 5.37 (d, *J* = 4.3 Hz, 1H), 5.02 (dd, *J* = 21.3, 5.6 Hz, 2H), 4.79 – 4.45 (m, 1H), 2.29 (dd, *J* = 12.7, 5.2 Hz, 4H), 2.08 (dt, *J* = 11.1, 5.4 Hz, 2H), 2.04 – 1.91 (m, 2H), 1.91 – 1.79 (m, 3H), 1.79 – 1.67 (m, 2H), 1.58 (dd, *J* = 15.2, 10.1 Hz, 3H), 1.48 (dd, *J* = 15.0, 8.3 Hz, 3H), 1.40 – 1.23 (m, 4H), 1.22 – 1.05 (m, 7H), 1.05 – 0.96 (m, 6H), 0.91 (d, *J* = 6.4 Hz, 4H), 0.86 (d, *J* = 6.6 Hz, 6H), 0.68 (s, 3H). ^{13}C NMR ($CDCl_3$, 300 MHz) δ 173.15 (C=O), 139.87 (C), 137.93 (CH), 122.76 (CH), 115.44 (CH_2), 73.94 (CH–O), 56.87 (CH), 56.33 (CH), 50.23 (CH), 42.49 (C), 39.92 (CH_2), 39.69 (CH_2), 38.33 (CH_2), 37.17 (CH_2), 36.77 (C), 36.36 (CH_2),

35.95 (CH), 34.11 (CH_2), 33.22 (CH_2), 32.05 (CH_2), 28.38 (CH_2), 28.17 (CH), 27.99 (CH_2), 24.44 (CH_2), 24.36 (CH_2), 24.00 (CH_2), 22.96 (CH), 22.71 (CH_3), 21.20 (CH_2), 19.47 (CH_3), 18.88 (CH_3), 12.01 (CH_3). IR (υ, cm^{-1}) 2935 (l, C–H), 1735 (l, C=O), 743 (i, C=H).

(8R,9S,13S,14S)–3–(Hex–5–en–1–yloxy)–13–methyl–6,7,8,9,11,12,13,14,15,16–decahy–dro–17H–cyclo–penta[a]phenanthren–17–one (S4):

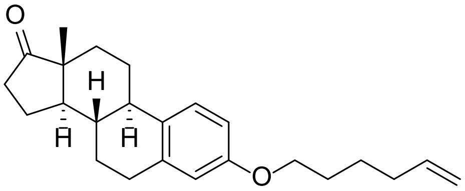

White solid (125mg, 20%);^{1}H NMR ($CDCl_3$, 300 MHz) δ 7.19 (d, J = 8.5 Hz, 1H), 6.71 (dd, J = 8.6, 2.7 Hz, 1H), 6.64 (d, J = 2.6 Hz, 1H), 5.83 (ddt, J = 16.9, 10.2, 6.7 Hz, 1H), 5.14 – 4.87 (m, 2H), 3.94 (t, J = 6.4 Hz, 2H), 2.91 (dd, J = 18.2, 13.5 Hz, 2H), 2.50 (dd, J = 18.3, 8.3 Hz, 1H), 2.44 – 2.34 (m, 1H), 2.23 (d, J = 16.4 Hz, 1H), 2.19 – 2.07 (m, 3H), 2.07 – 1.89 (m, 3H), 1.85 – 1.71 (m, 2H), 1.64 (dd, J = 11.7, 4.0 Hz, 1H), 1.60 – 1.53 (m, 6H), 1.52 – 1.38 (m, 4H).

^{13}C NMR ($CDCl_3$, 300 MHz) δ 221.03 (C=O), 157.30 (C–O), 138.73 (CH), 137.85 (C), 132.05(C), 126.43 (CH), 114.83 (CH_2), 114.74 (CH), 112.29 (CH), 67.86 (CH_2–O), 50.62 (CH), 48.18 (C), 44.17 (CH), 38.58 (CH), 36.02 (CH_2), 33.58 (CH_2), 31.77 (CH_2), 29.81 (CH_2), 28.94 (CH_2), 26.74 (CH_2), 26.09 (CH_2), 25.51 (CH_2), 21.75 (CH_2), 14.02 (CH_3). IR (υ, cm^{-1}) 2929 (i, C–H), 2861 (m, C–H), 1740 (vi, C=O), 1499 (m, C–O).

NMR Spectra

^{1}H–NMR **3**

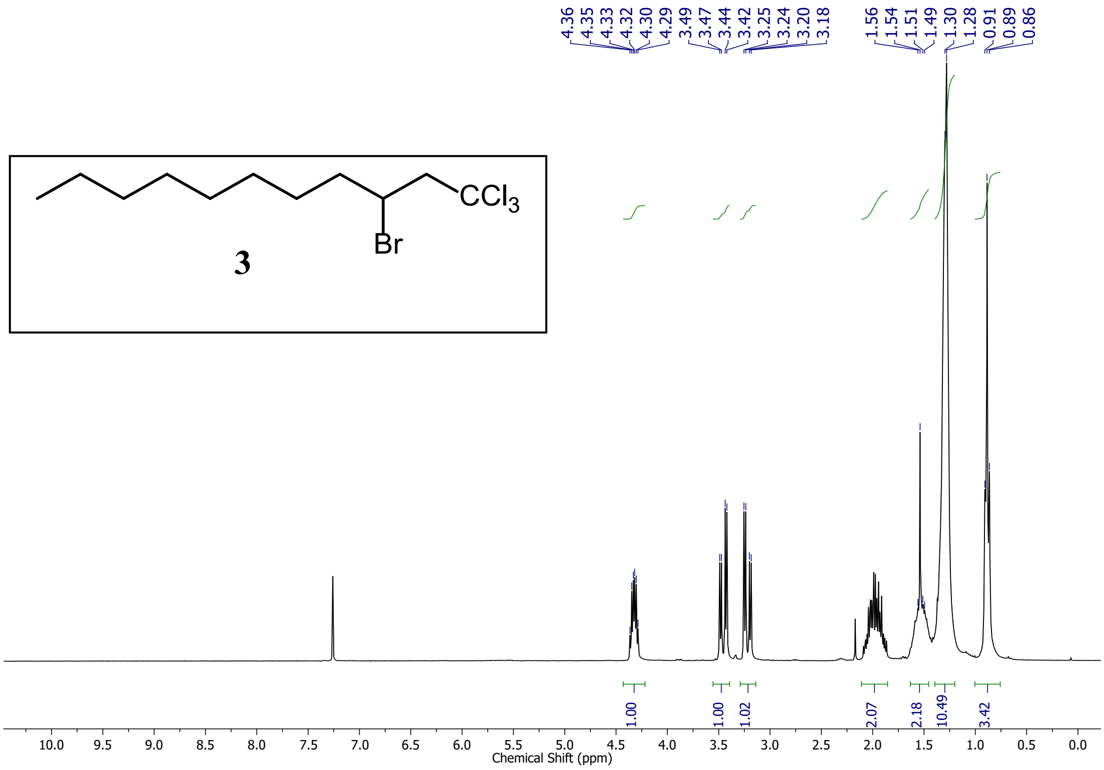

^{13}C–NMR **3**

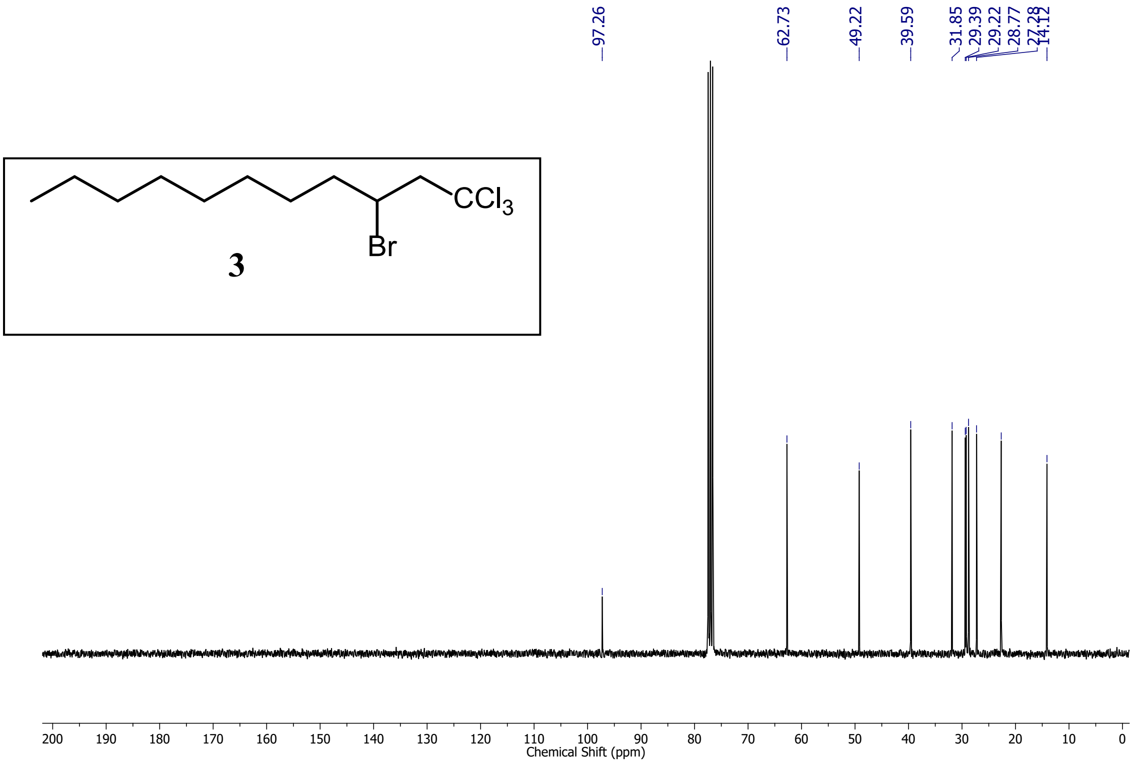

DEPT **3** (CH_2s upside)

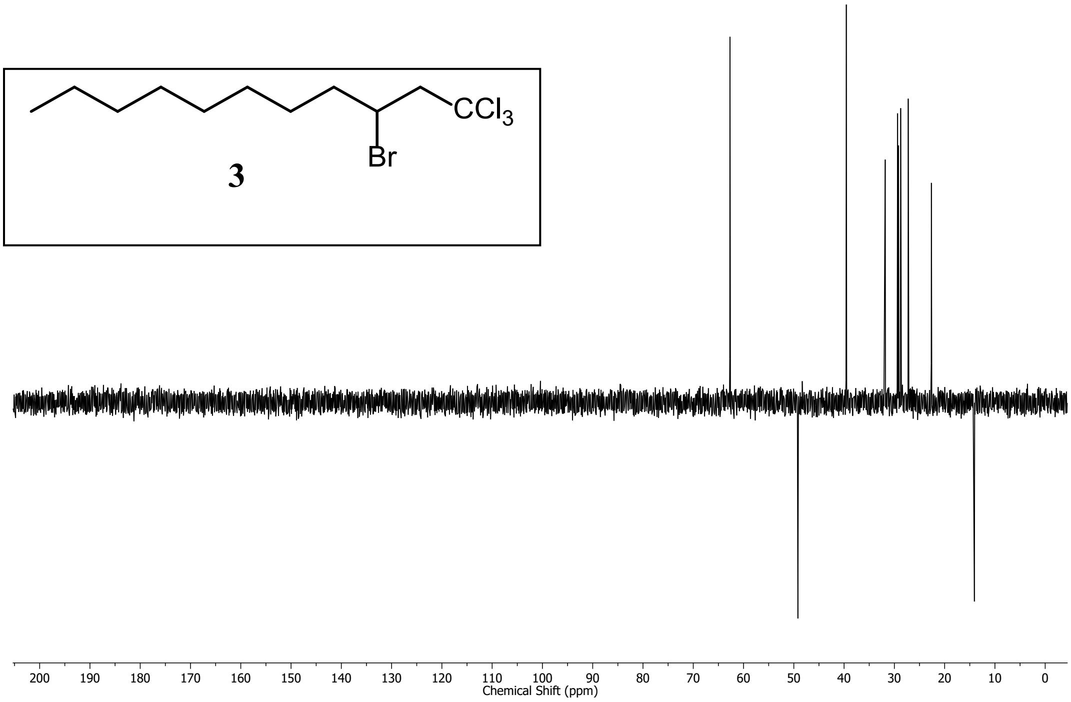

^{1}H–NMR **6** (impurities correspond to starting alkene, isomer or hydrogenated products)

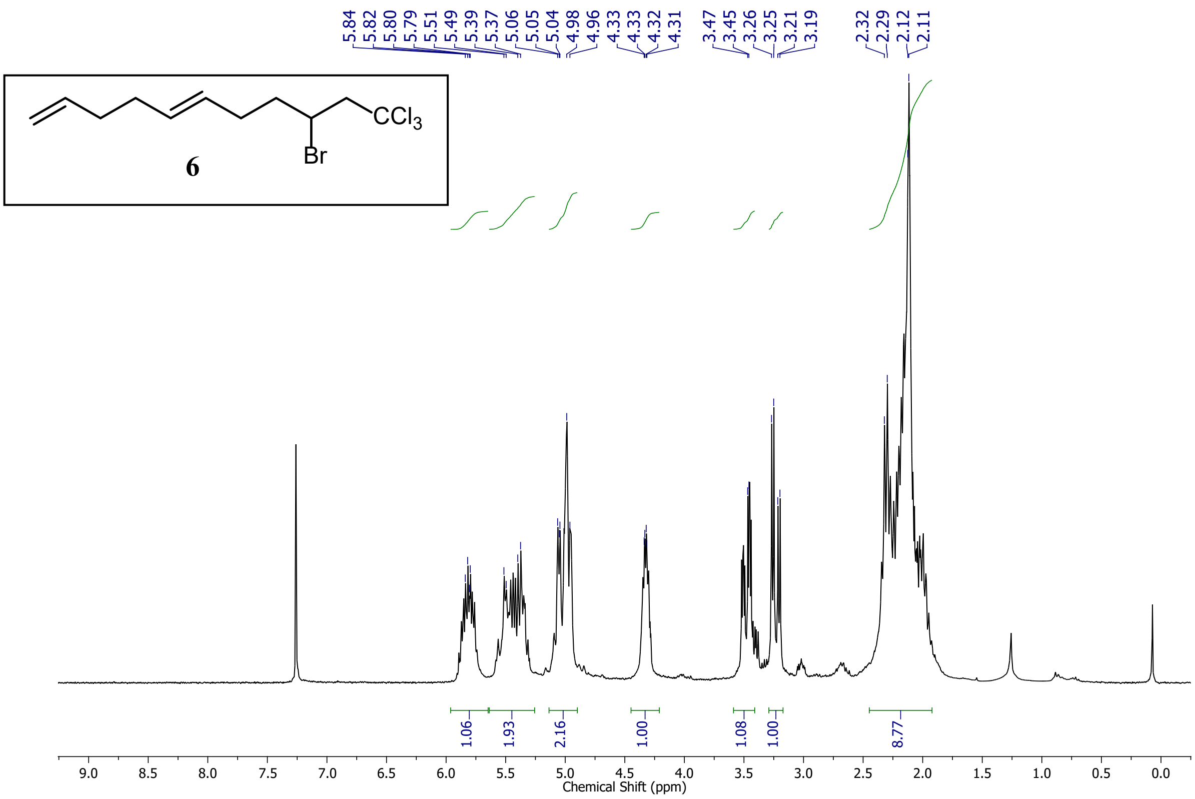

^{13}C–NMR **6**

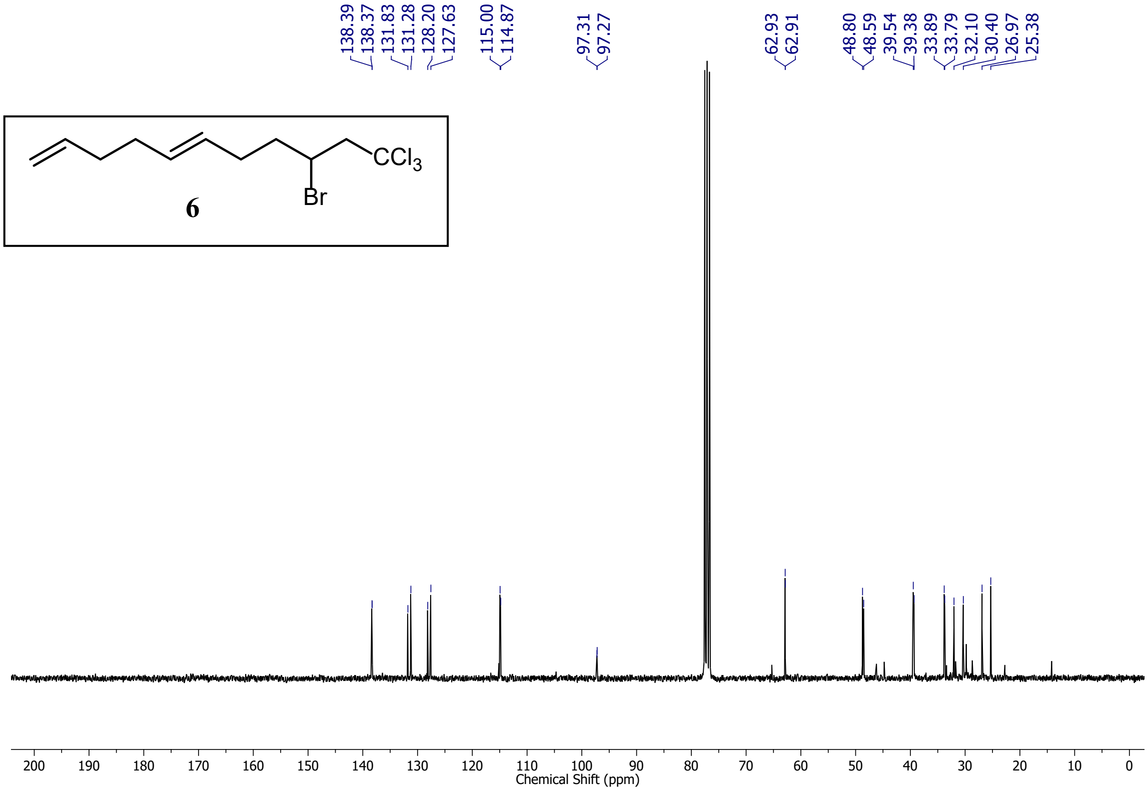

DEPT **6** (CH_2s upside)

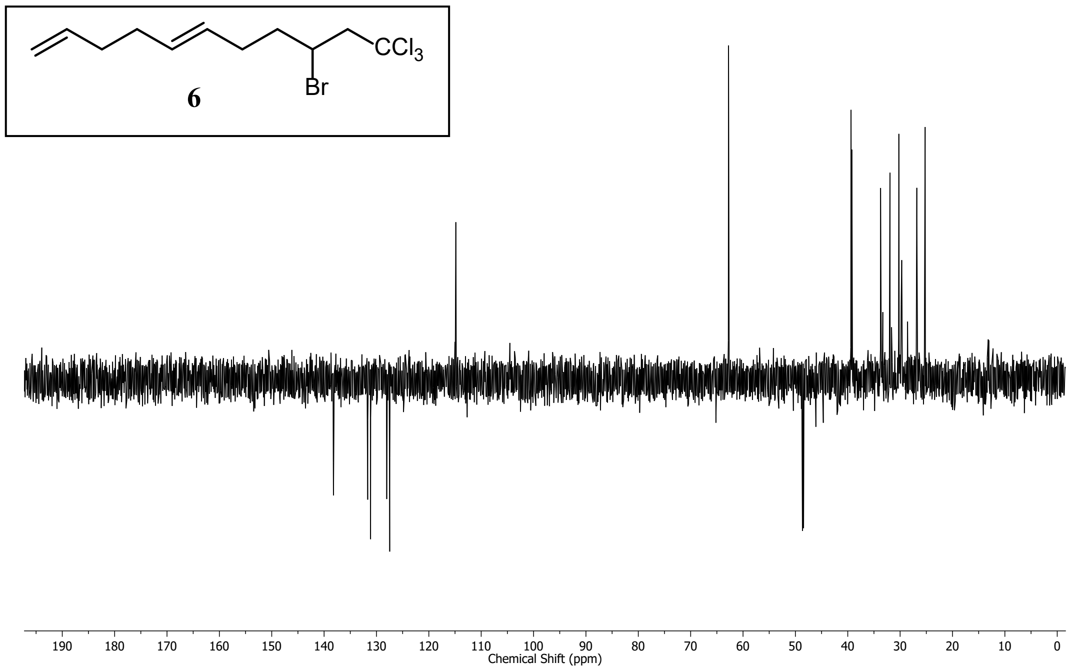

^{1}H–NMR **7** (impurities correspond to starting alkene, isomer or hydrogenated products)

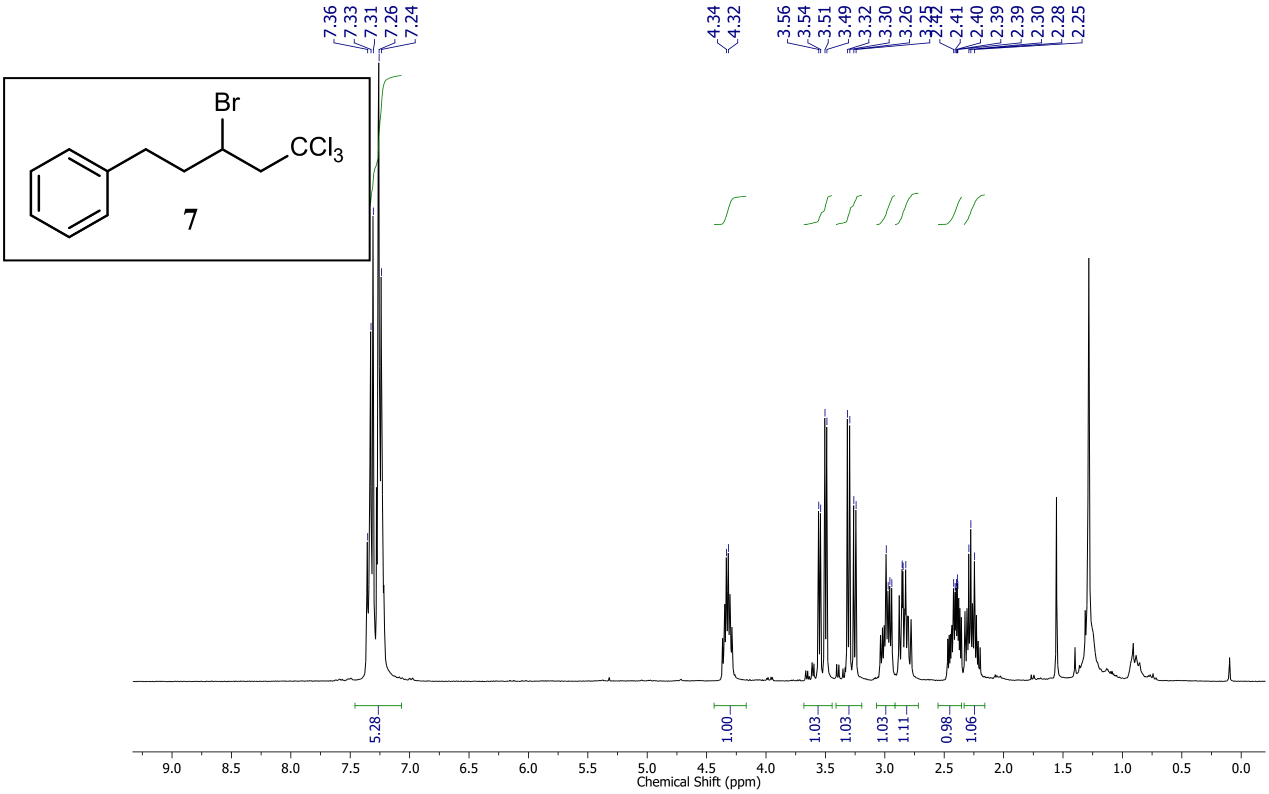

^{13}C–NMR **7**

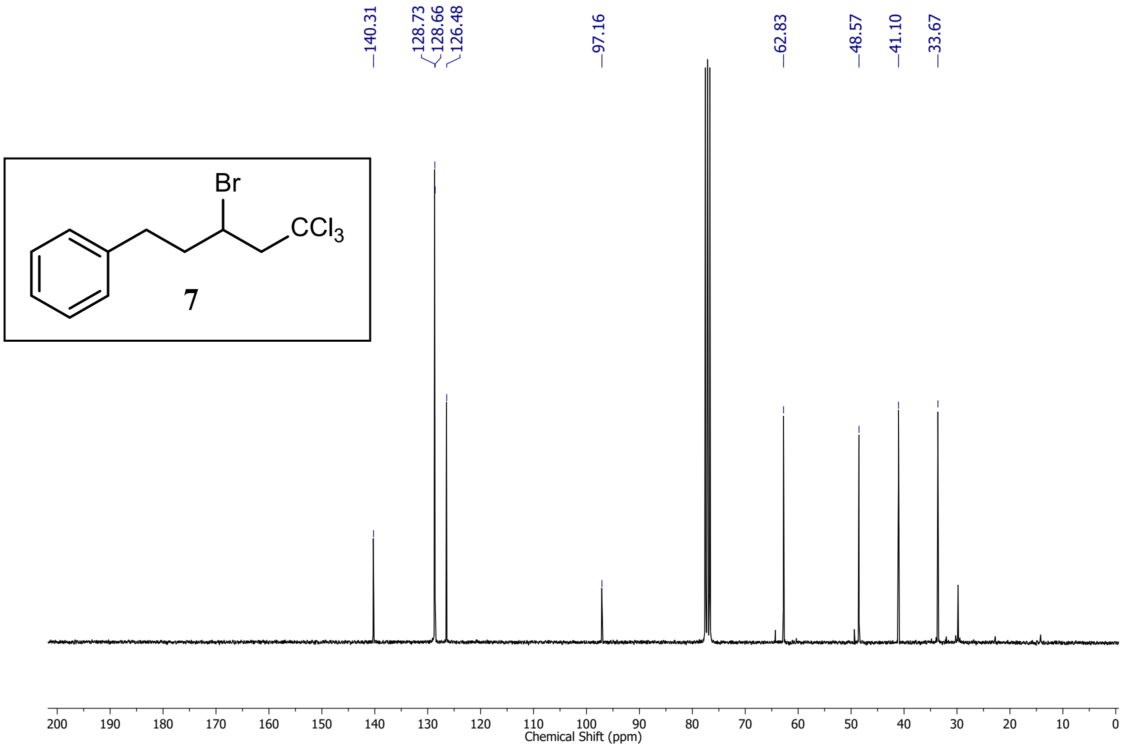

DEPT **7** (CH_2s downside)

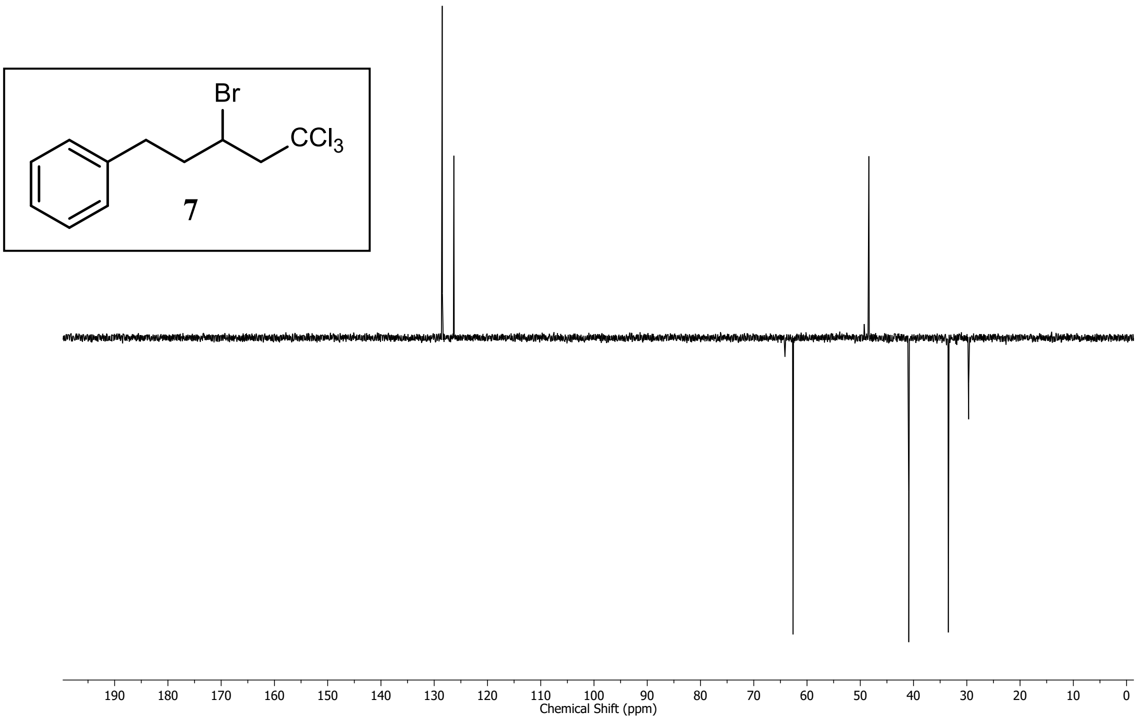

^{1}H–NMR **8** (impurities correspond to starting alkene, isomer or hydrogenated products)

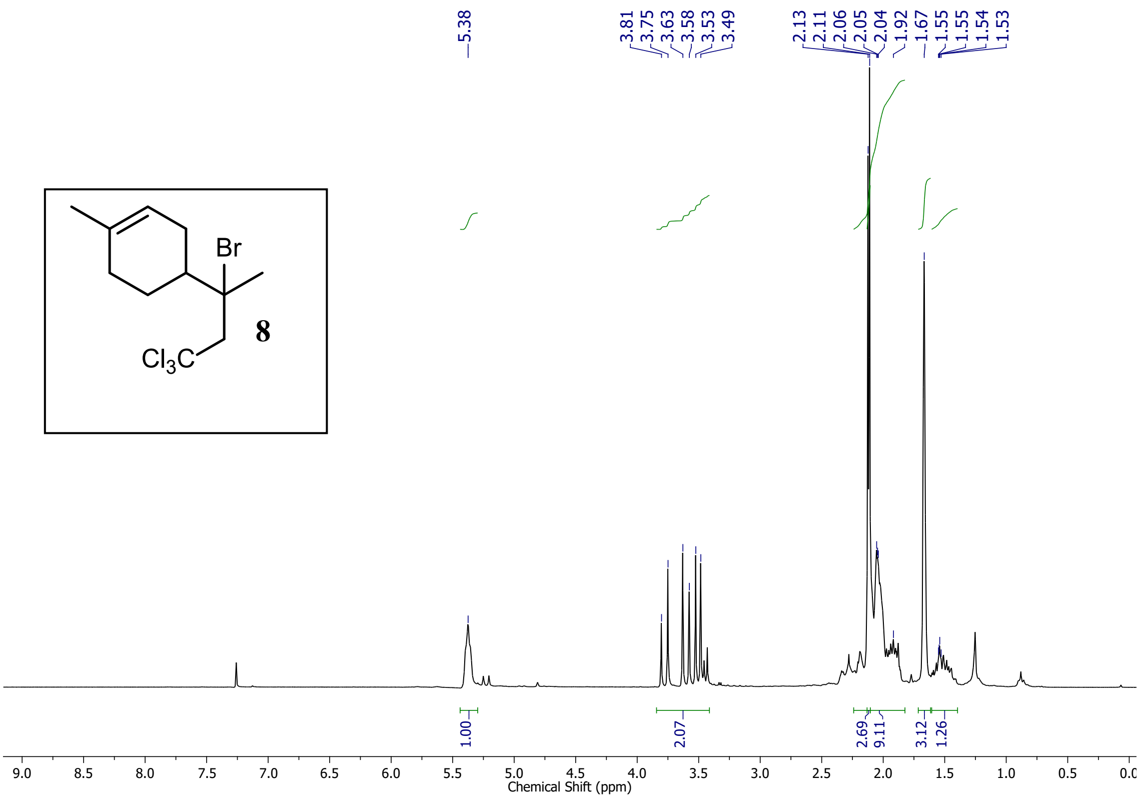

^{13}C–NMR **8**

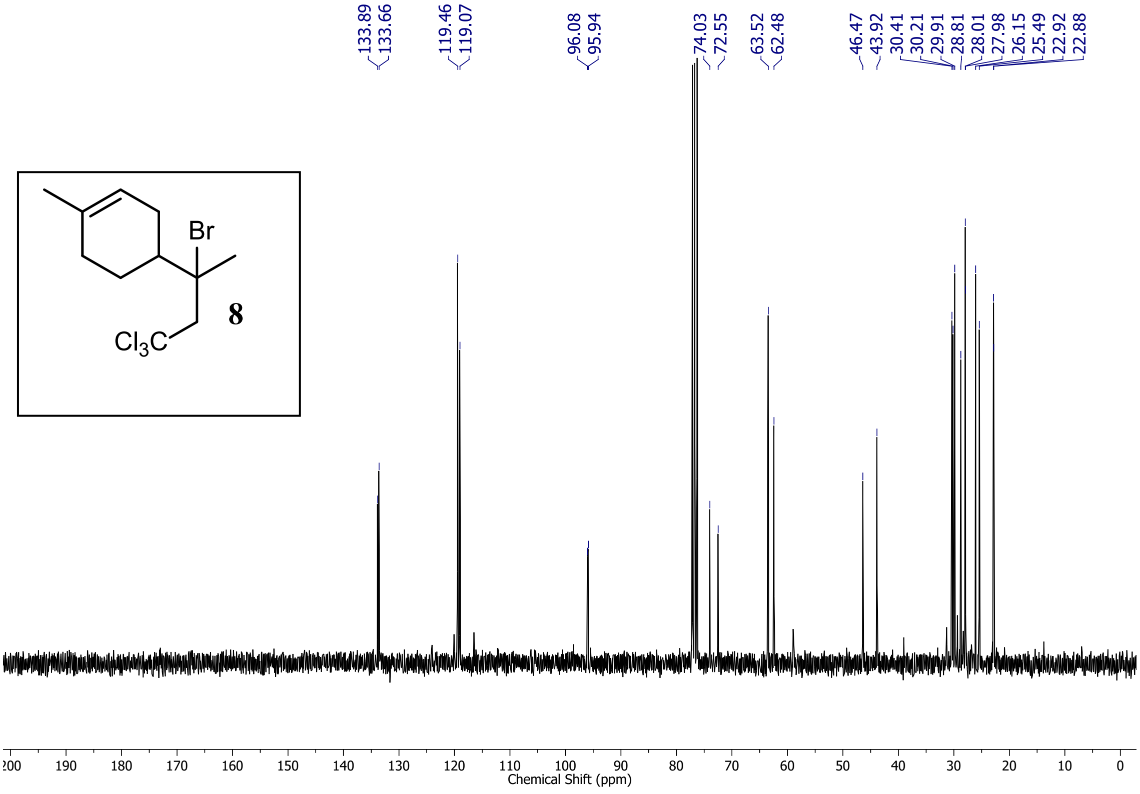

DEPT **8** (CH_2s downside)

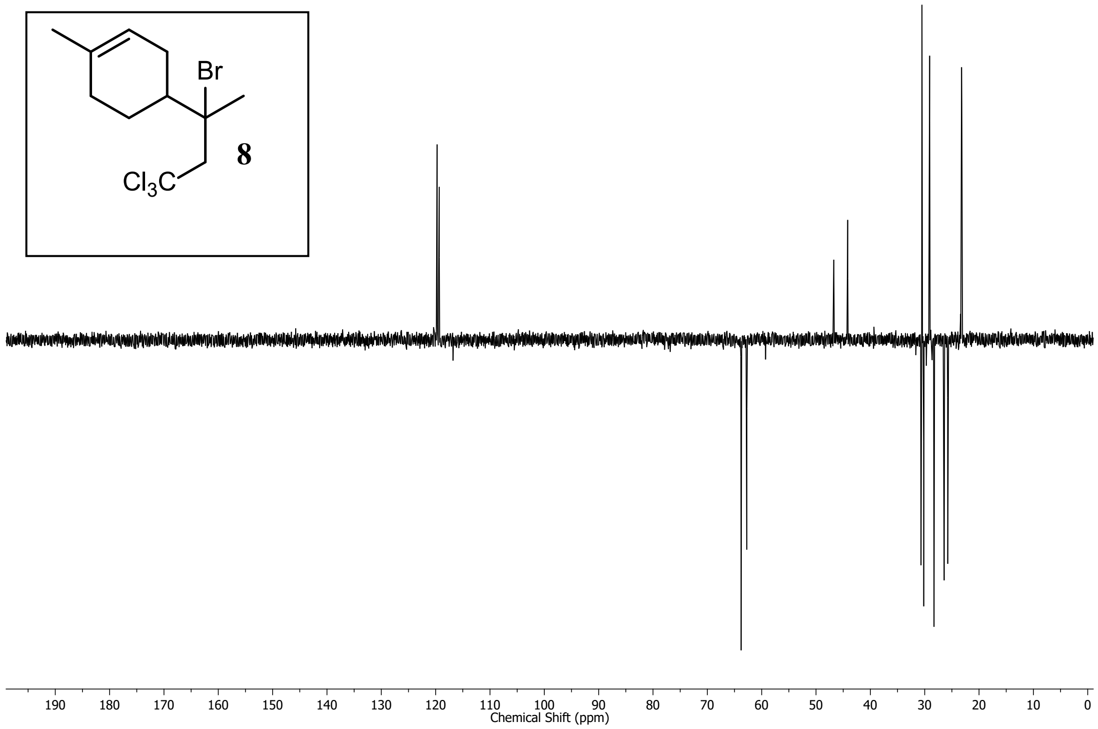

^{1}H–NMR **9** (impurities correspond to starting alkene, isomer or hydrogenated products)

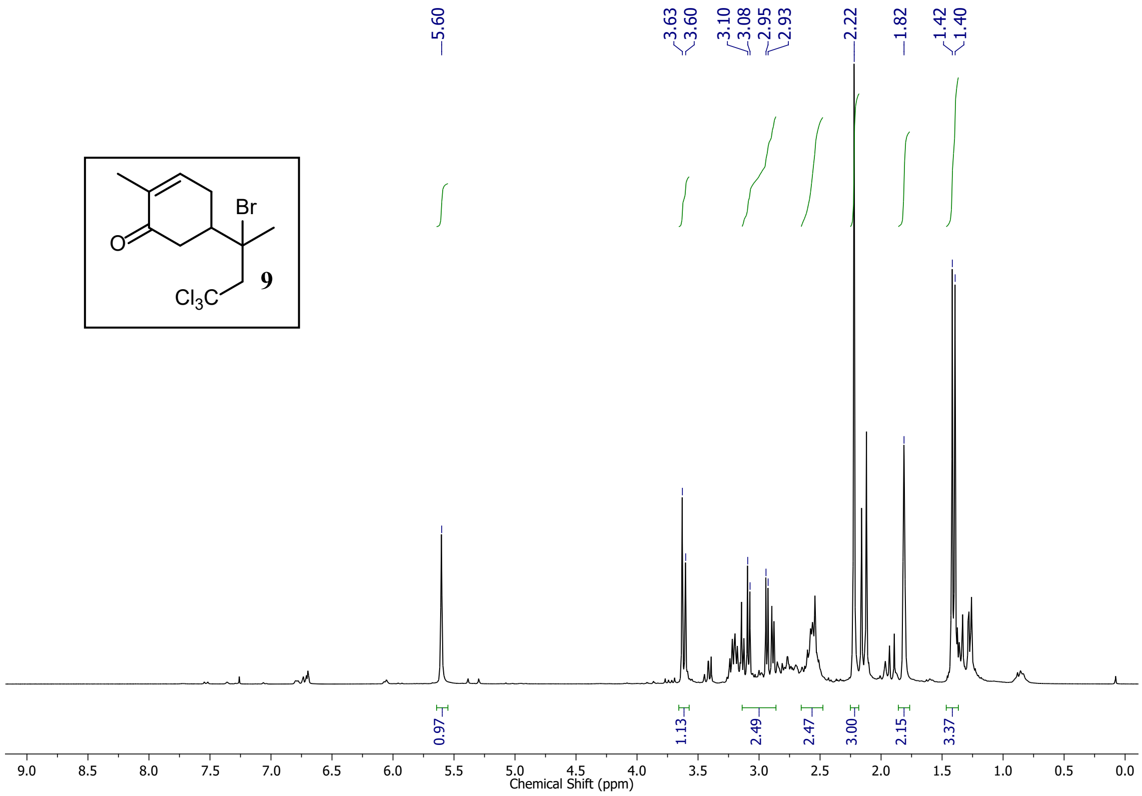

^{13}C–NMR **9**

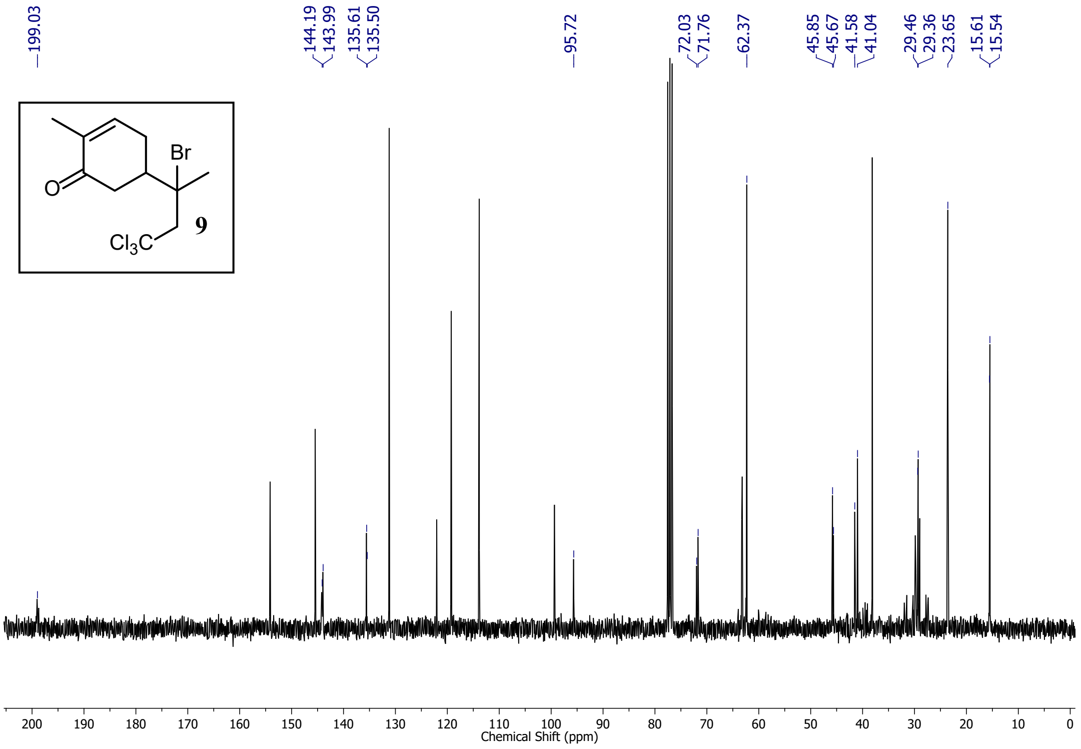

DEPT **9** (CH_2s downside)

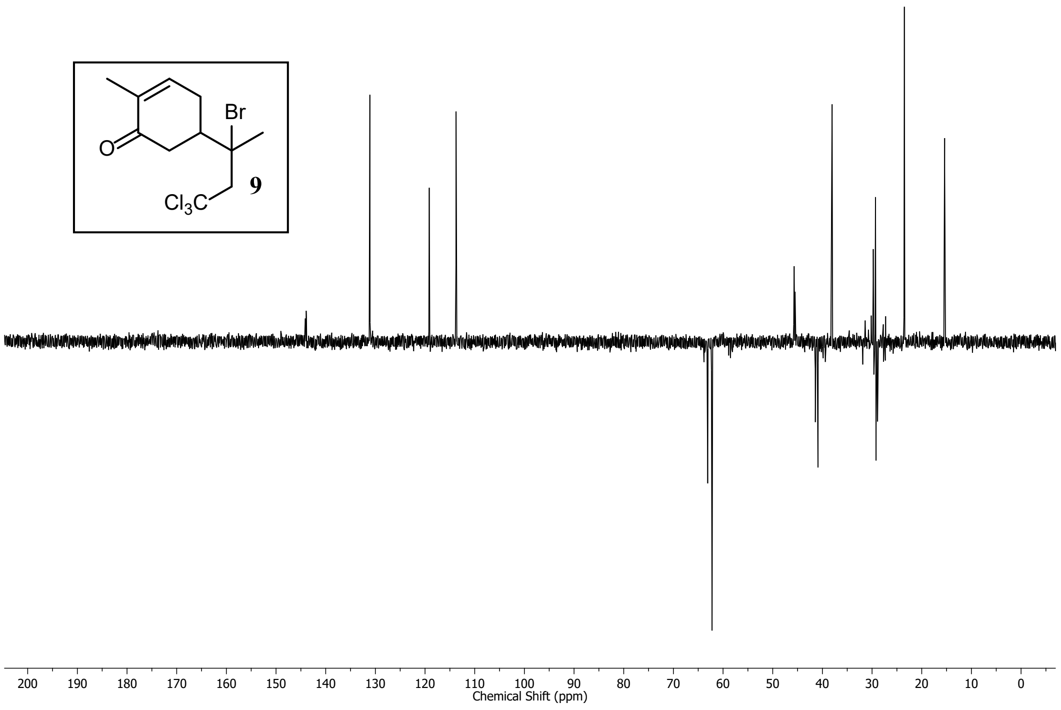

^{1}H–NMR **10**

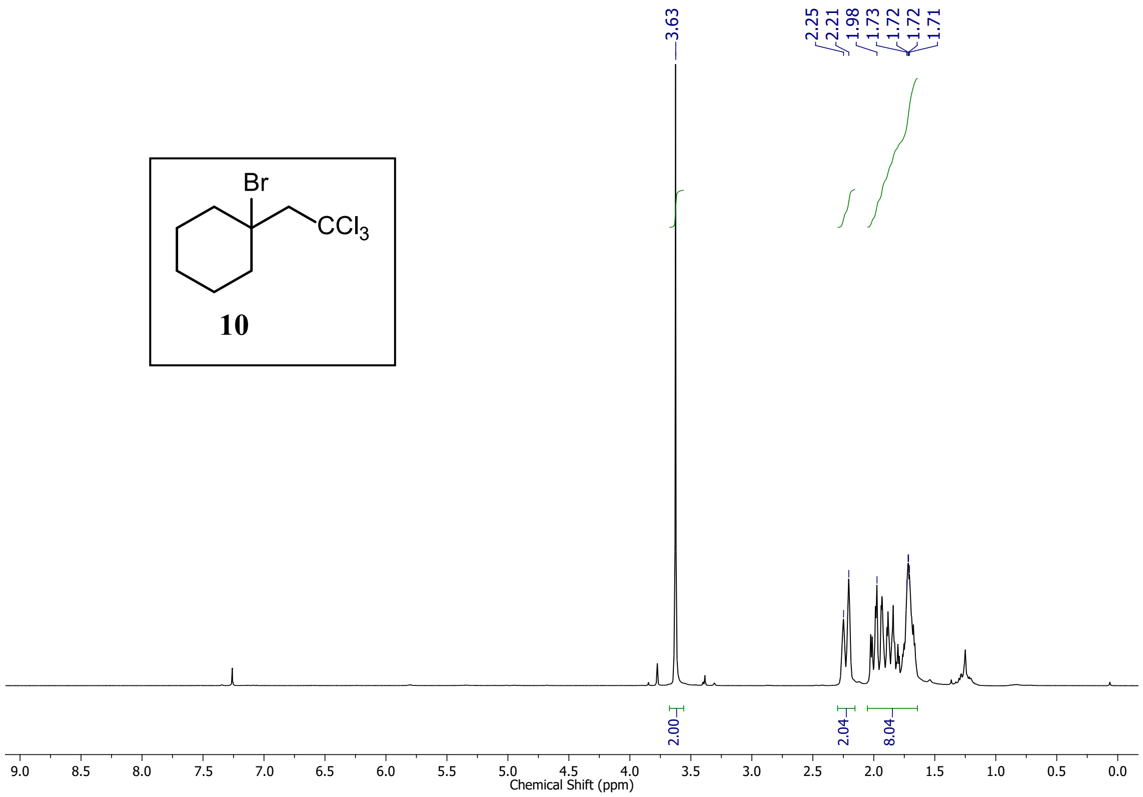

^{13}C–NMR **10**

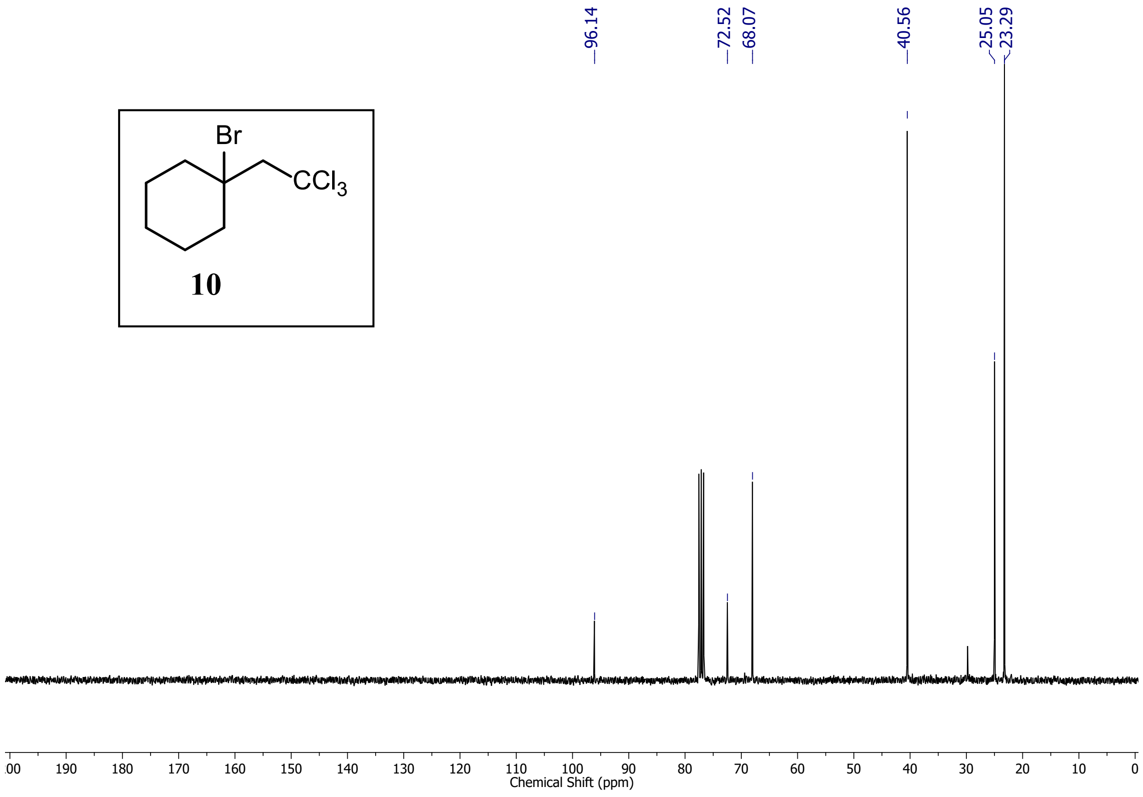

DEPT **10**

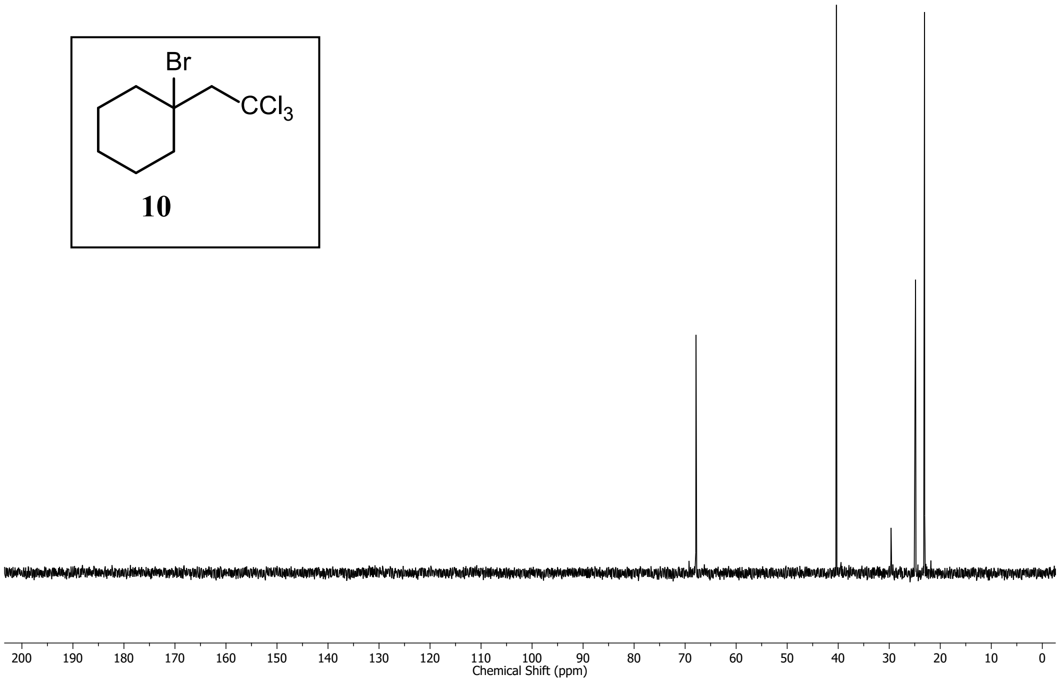

^{1}H–NMR **11**

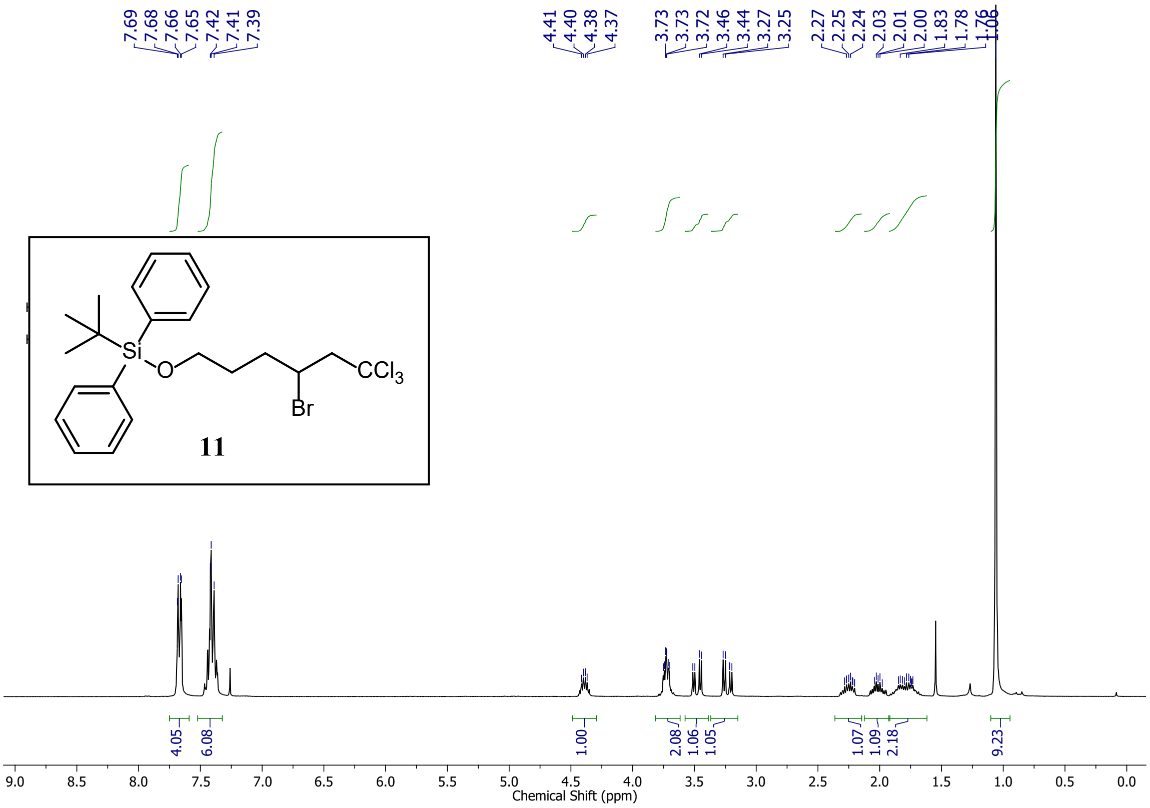

^{13}C–NMR **11**

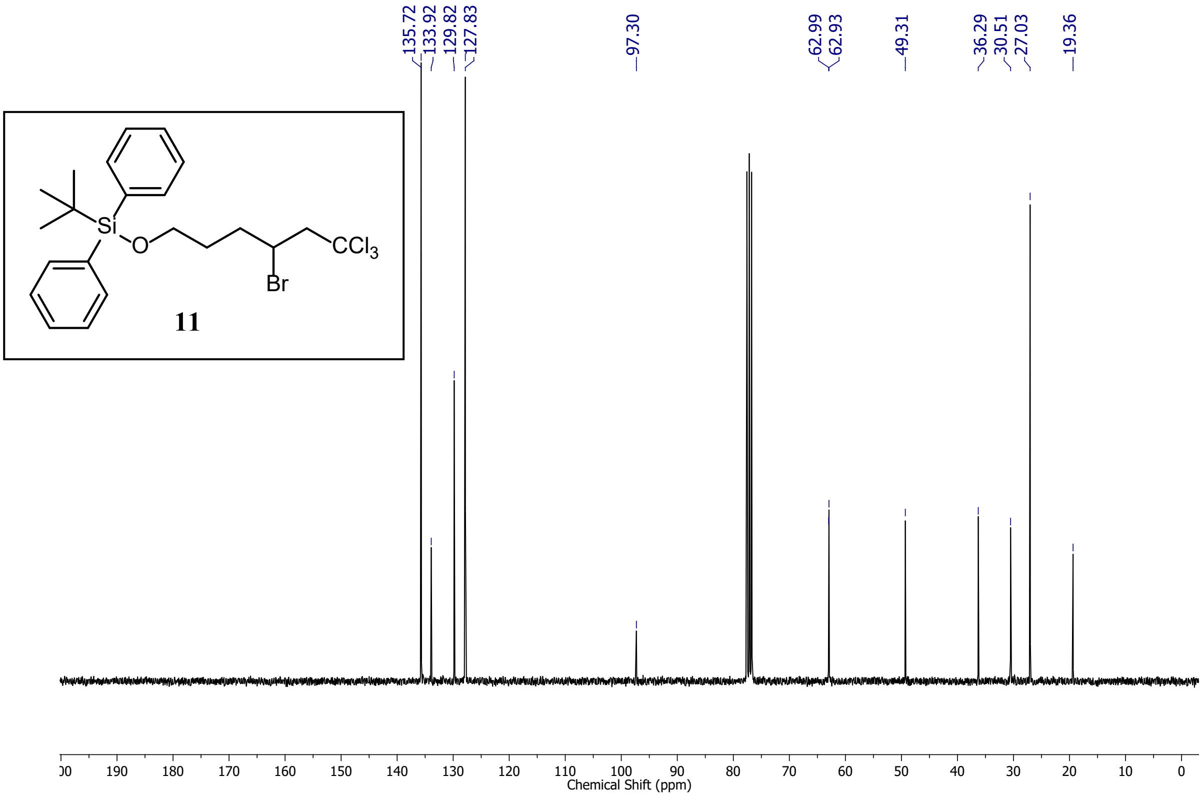

DEPT **11** **(**CH_2s downside)

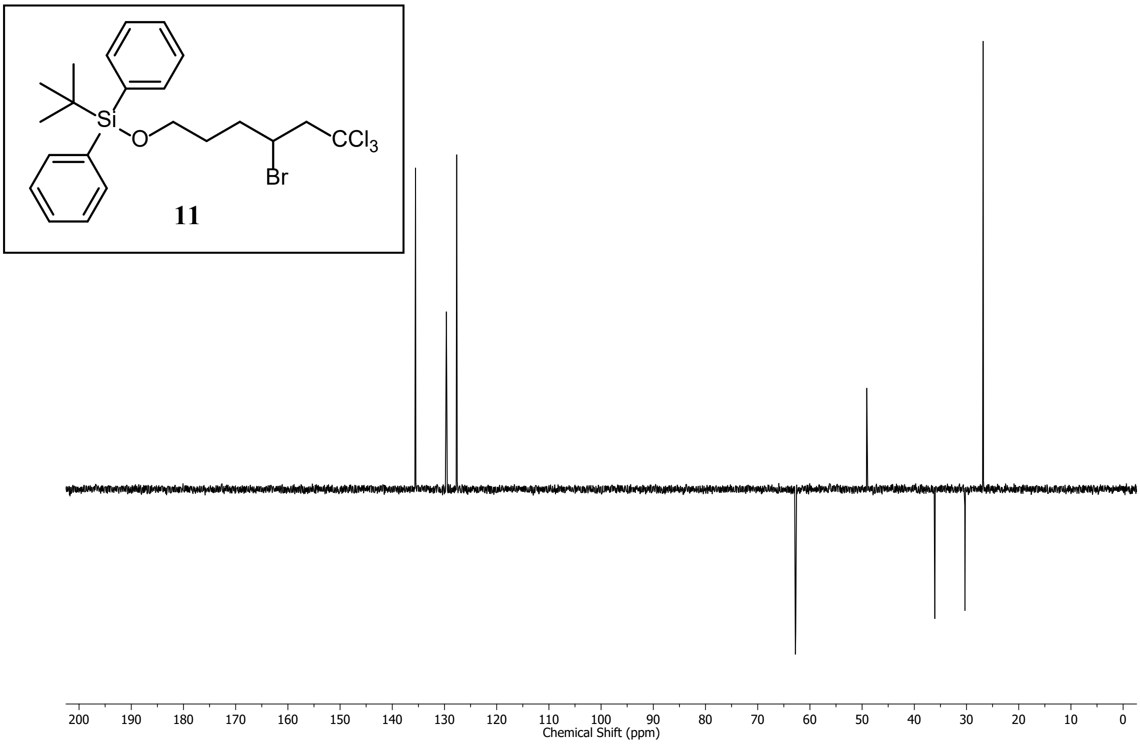

^{1}H–NMR **12** (impurities correspond to starting alkene, isomer or hydrogenated products)

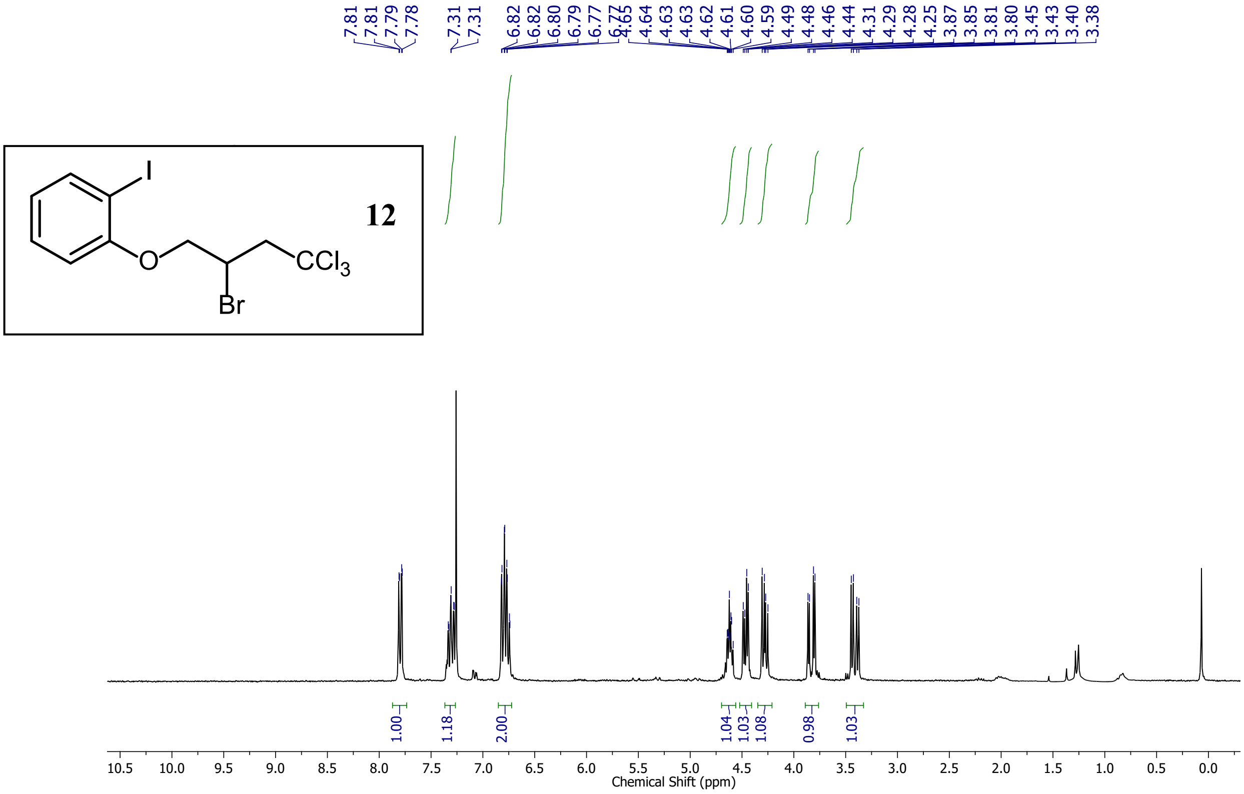

^{13}C–NMR **12**

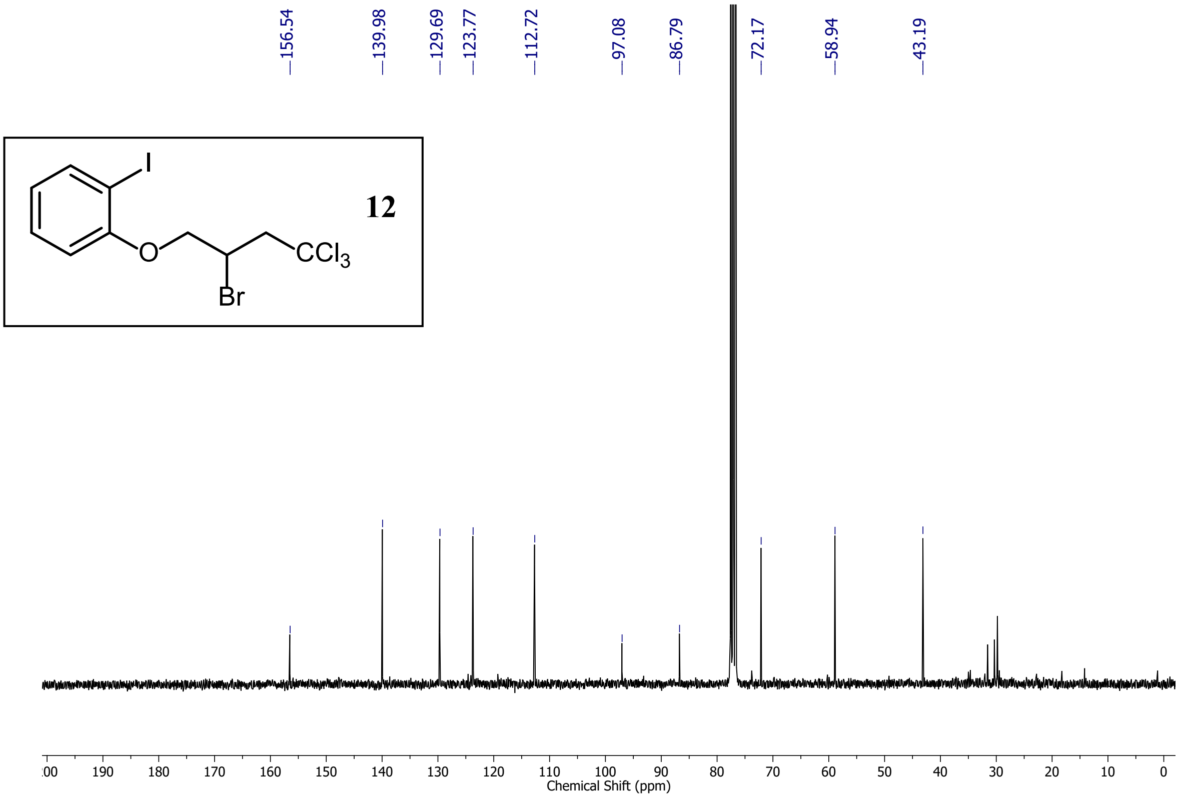

DEPT **12** (CH_2s upside)

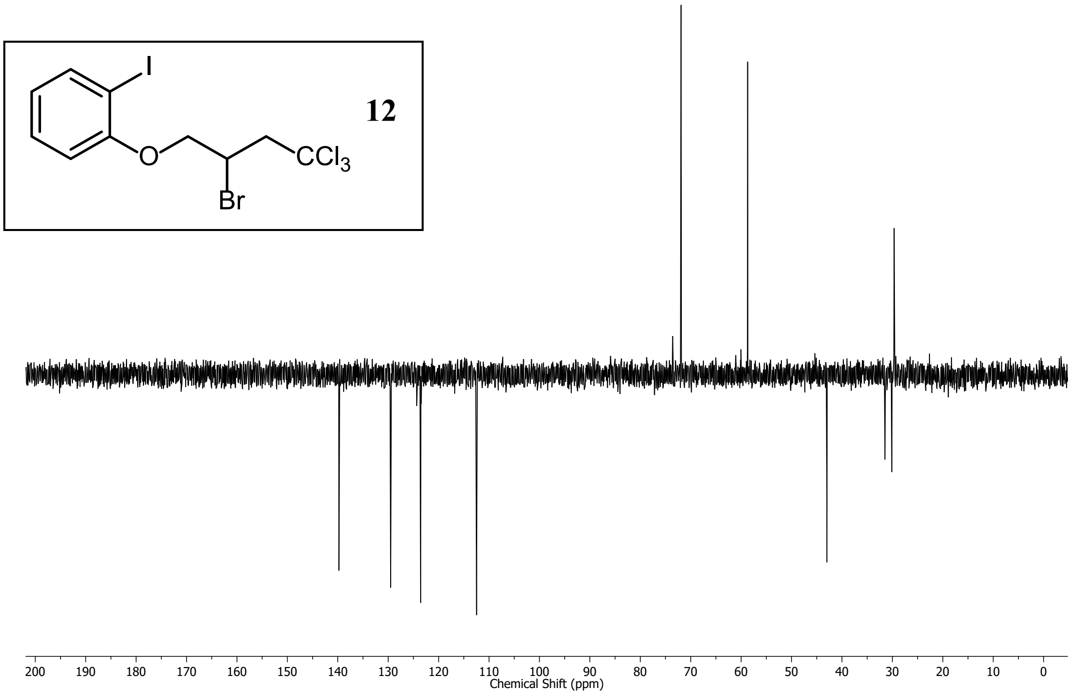

^{1}H–NMR **13**

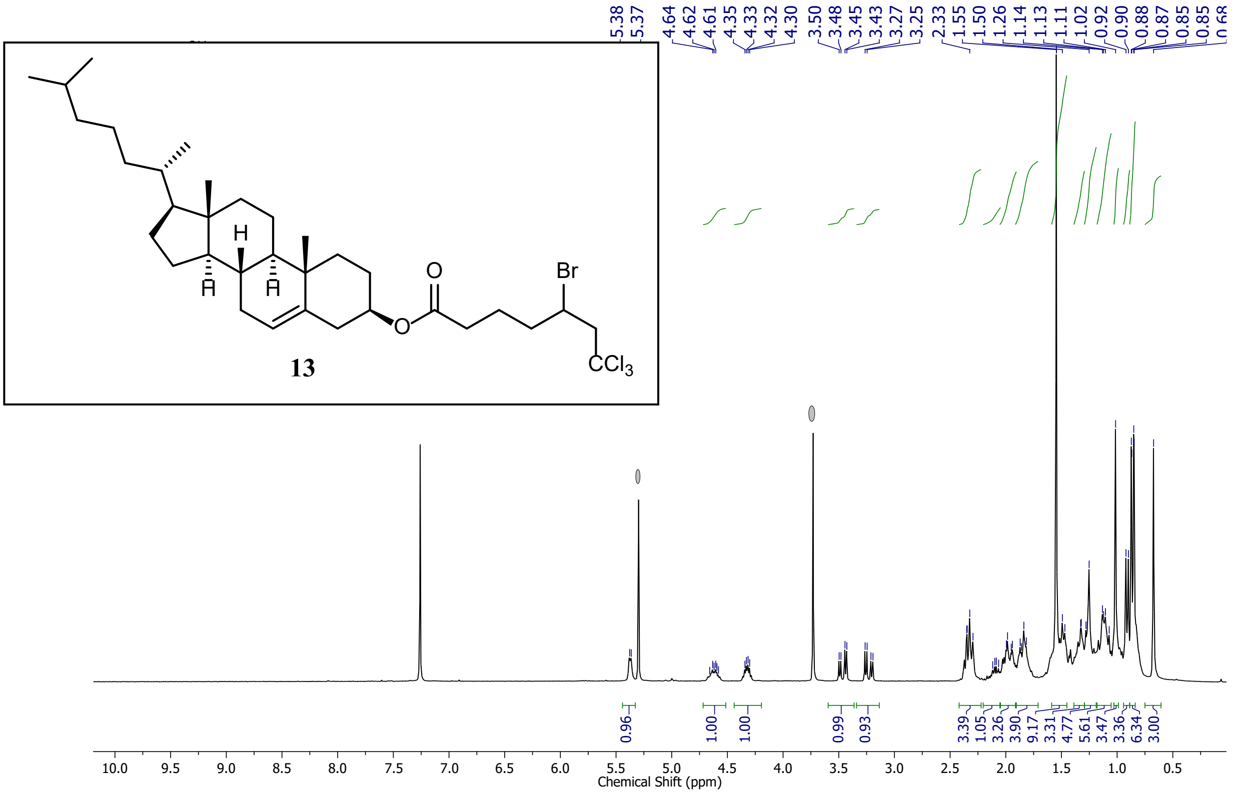

^{13}C–NMR **13**

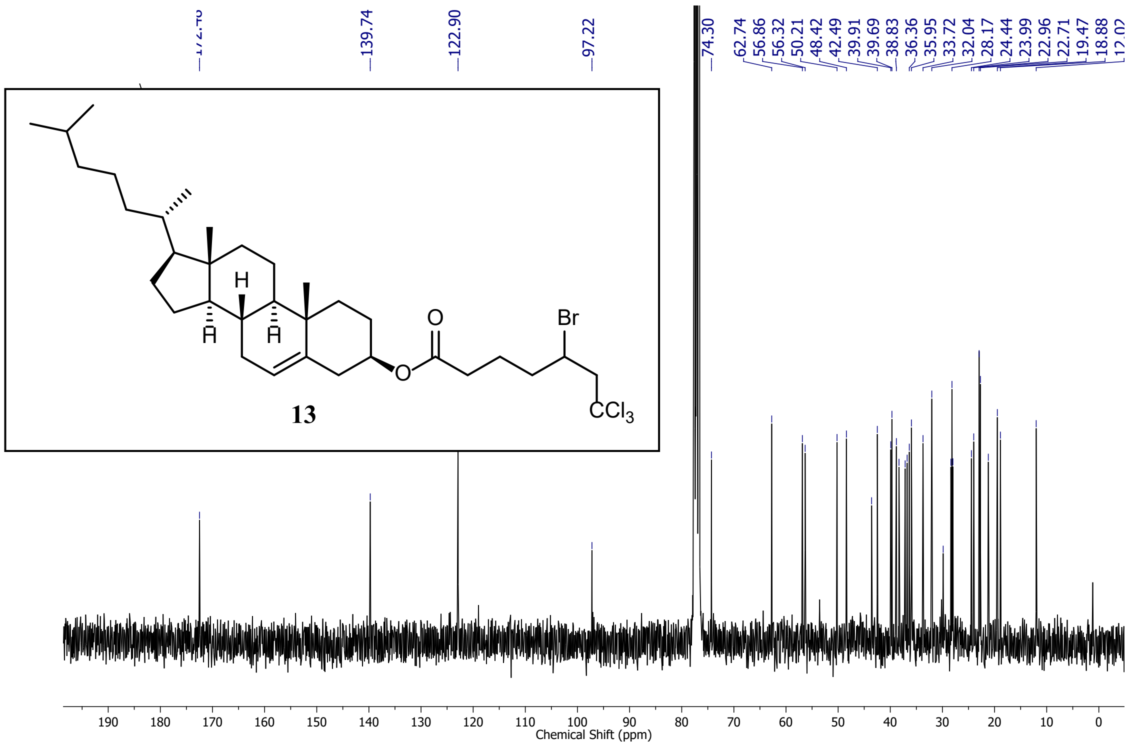

DEPT **13** (CH_2s downside)

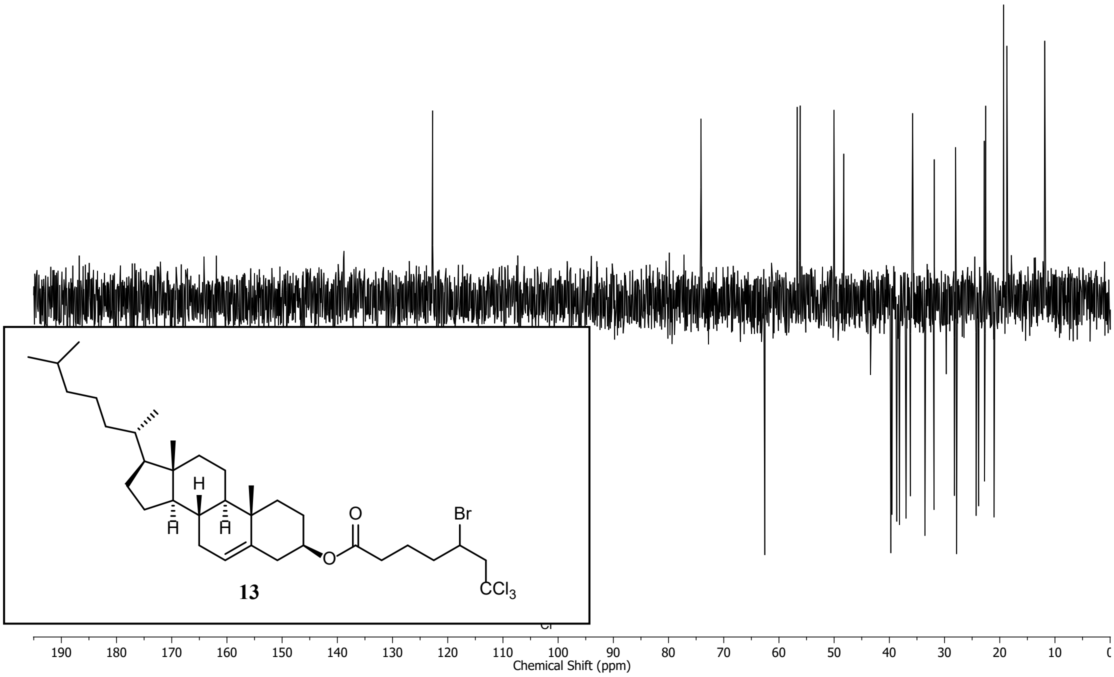

^{1}H–NMR **14** (impurities correspond to starting alkene, isomer or hydrogenated products)

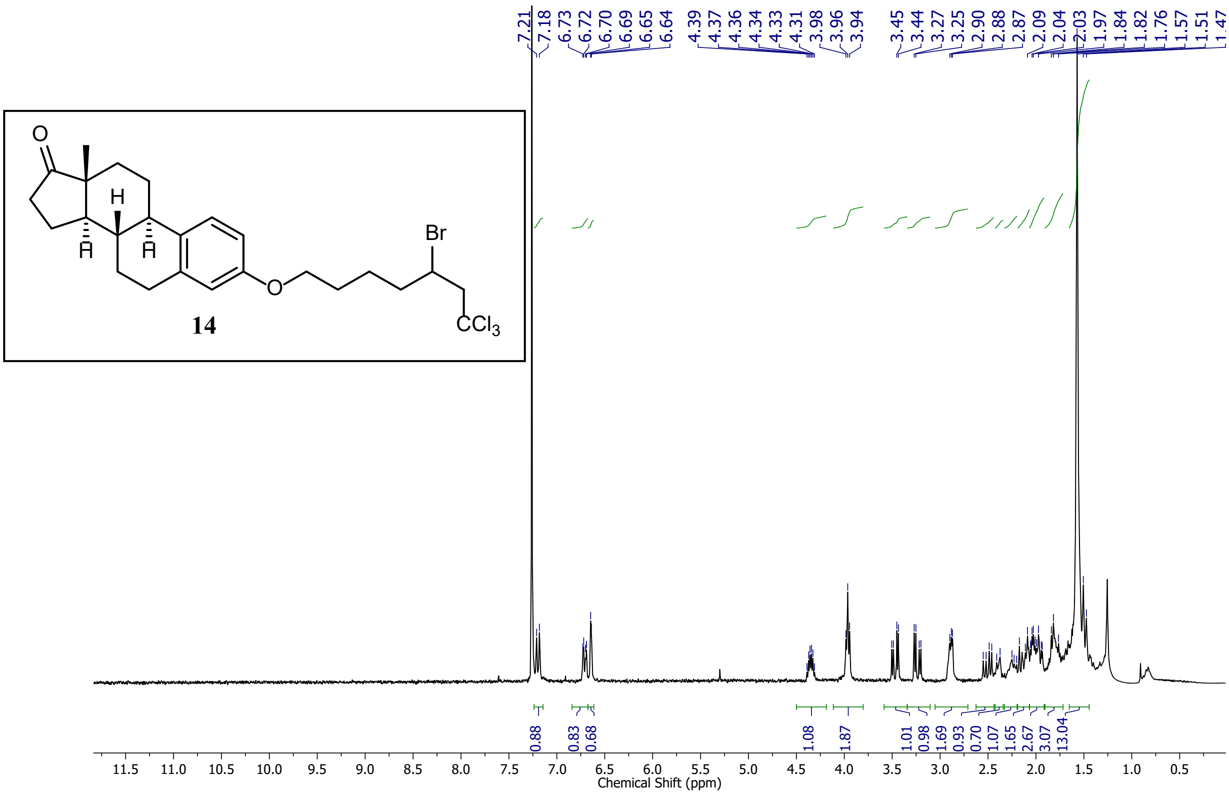

^{13}C–NMR **14**

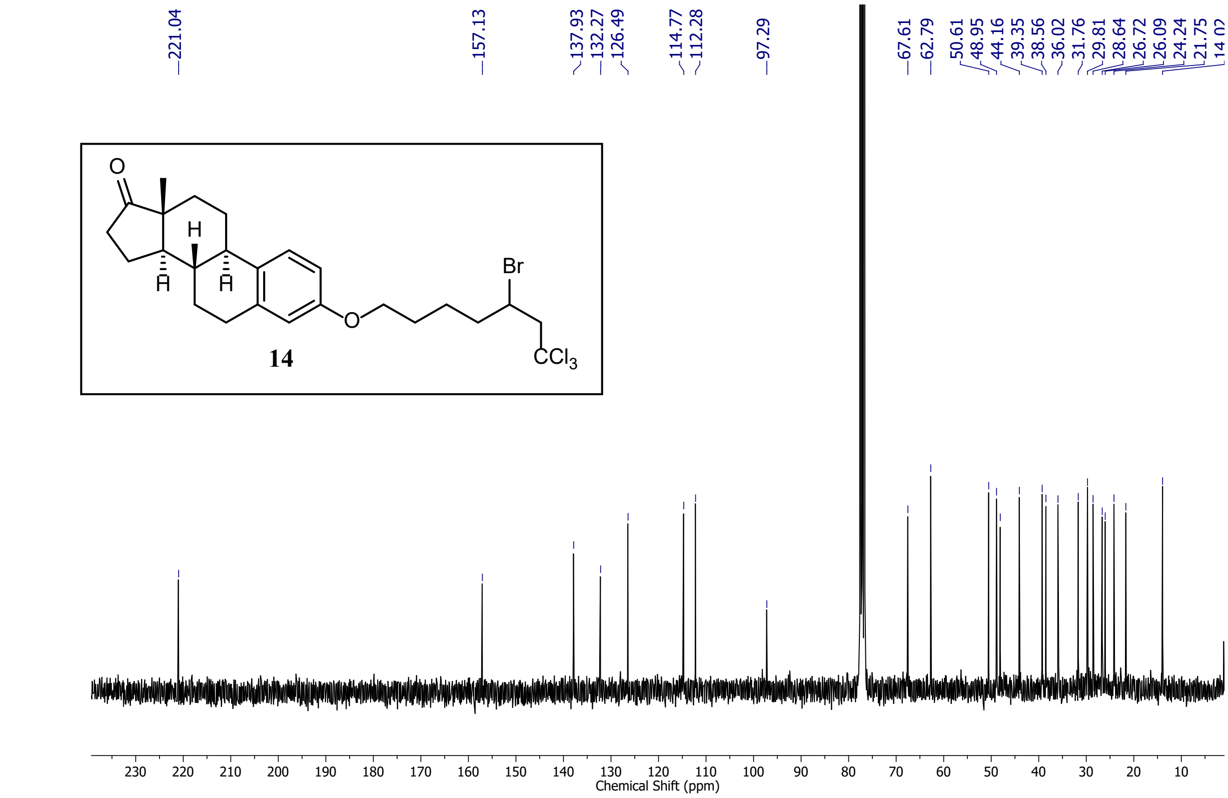

DEPT **14** (CH_2s upside)

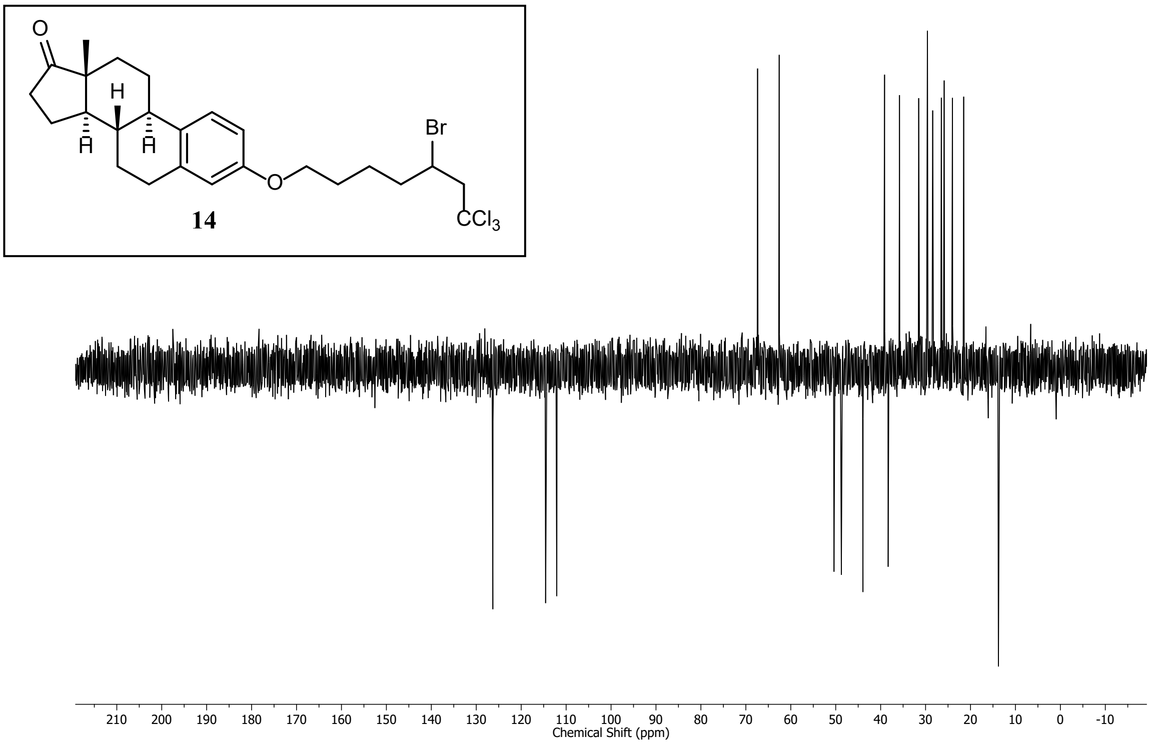

^{1}H–NMR **15**

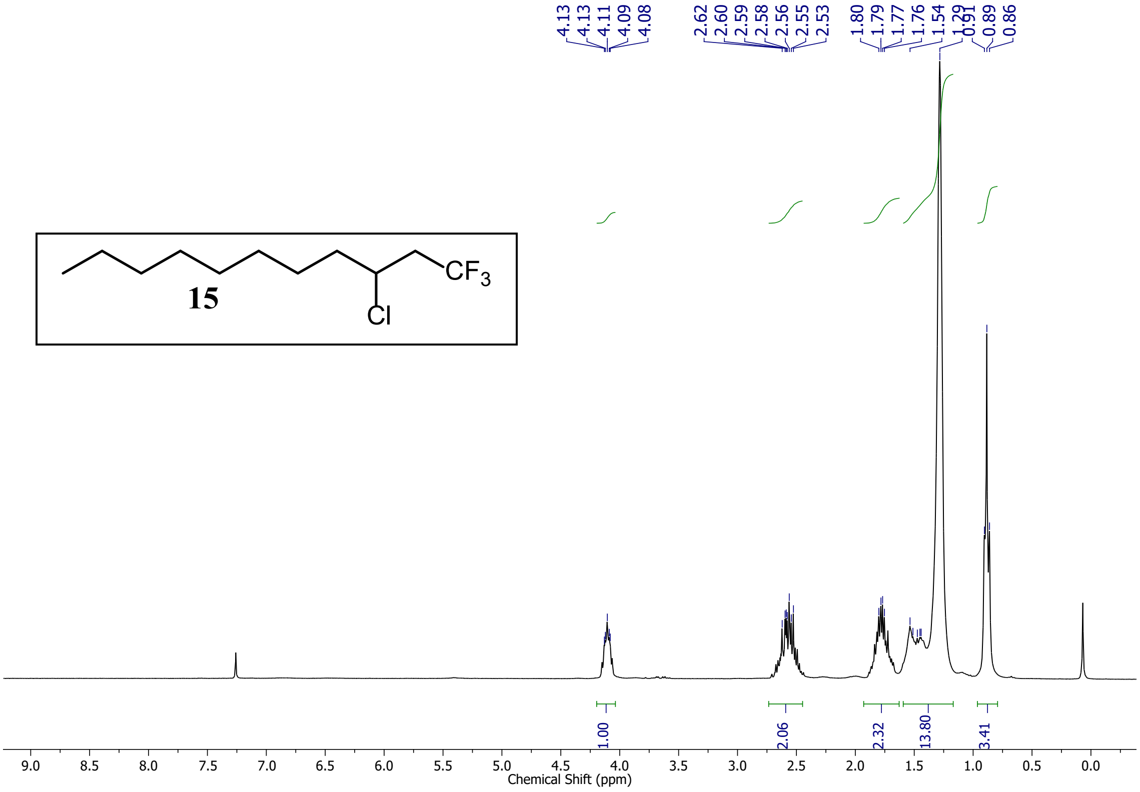

^{13}C–NMR **15**

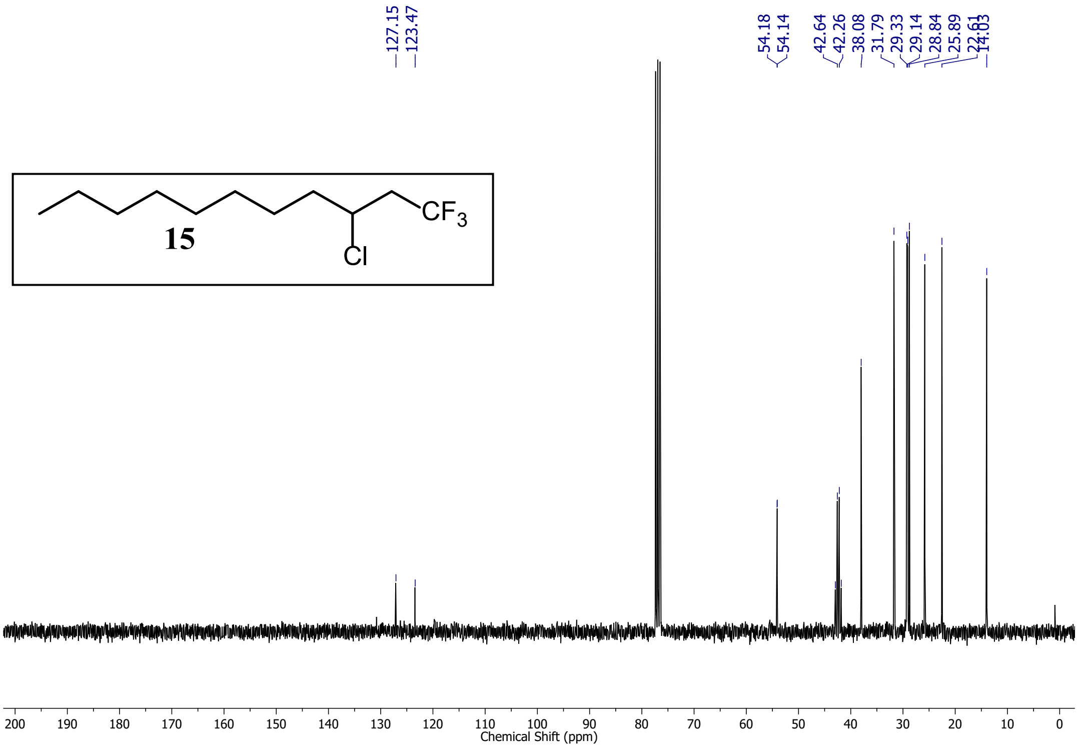

^{19}F–NMR **15**

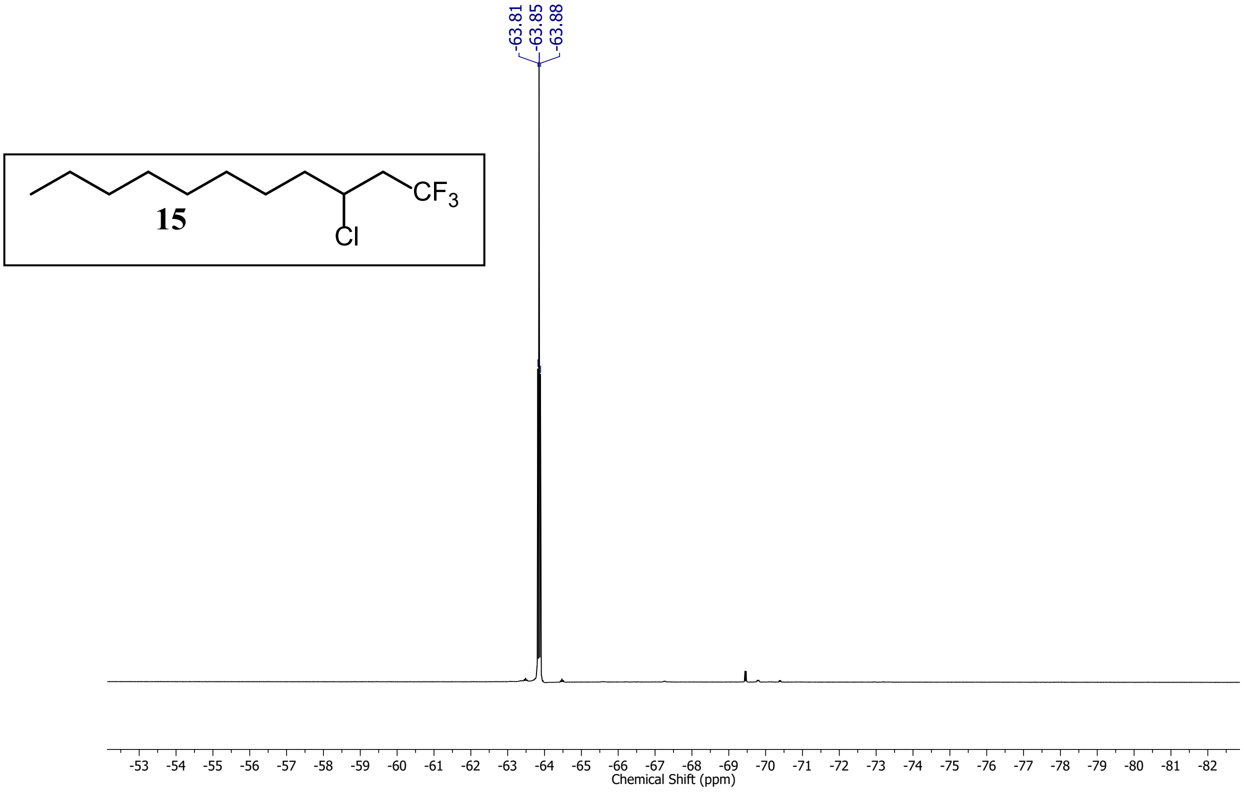

DEPT **15** (CH_2s upside)

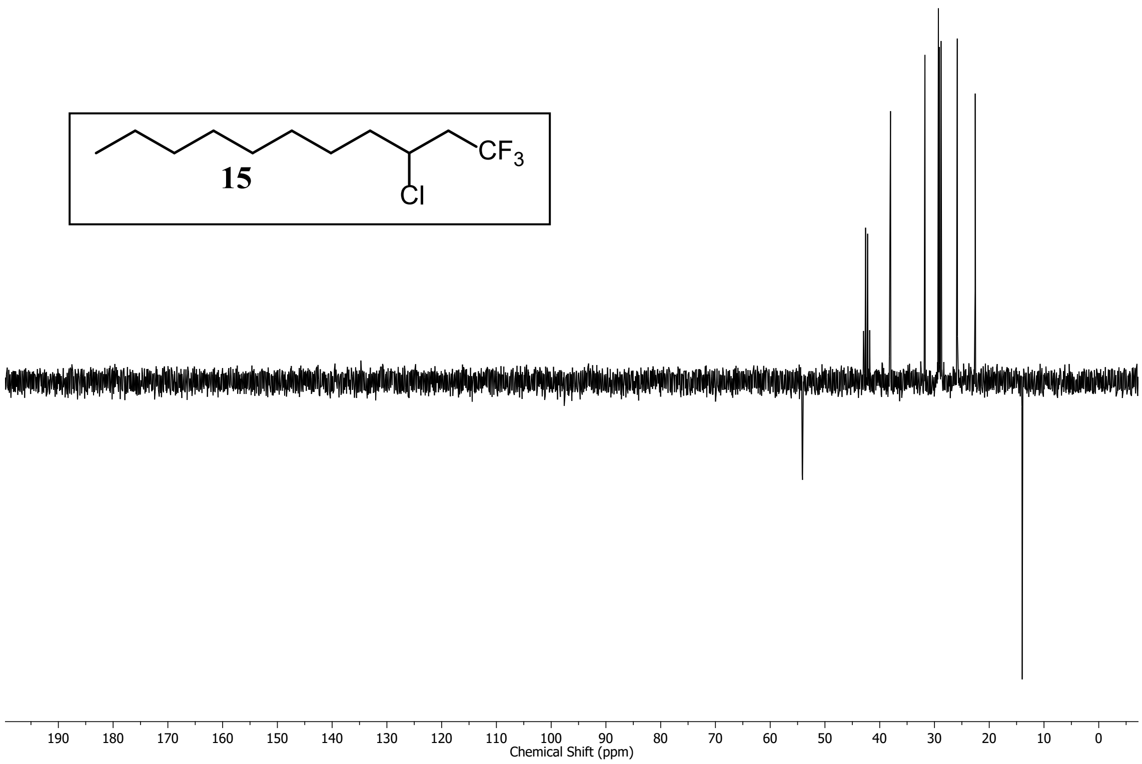

^{1}H–NMR **16**

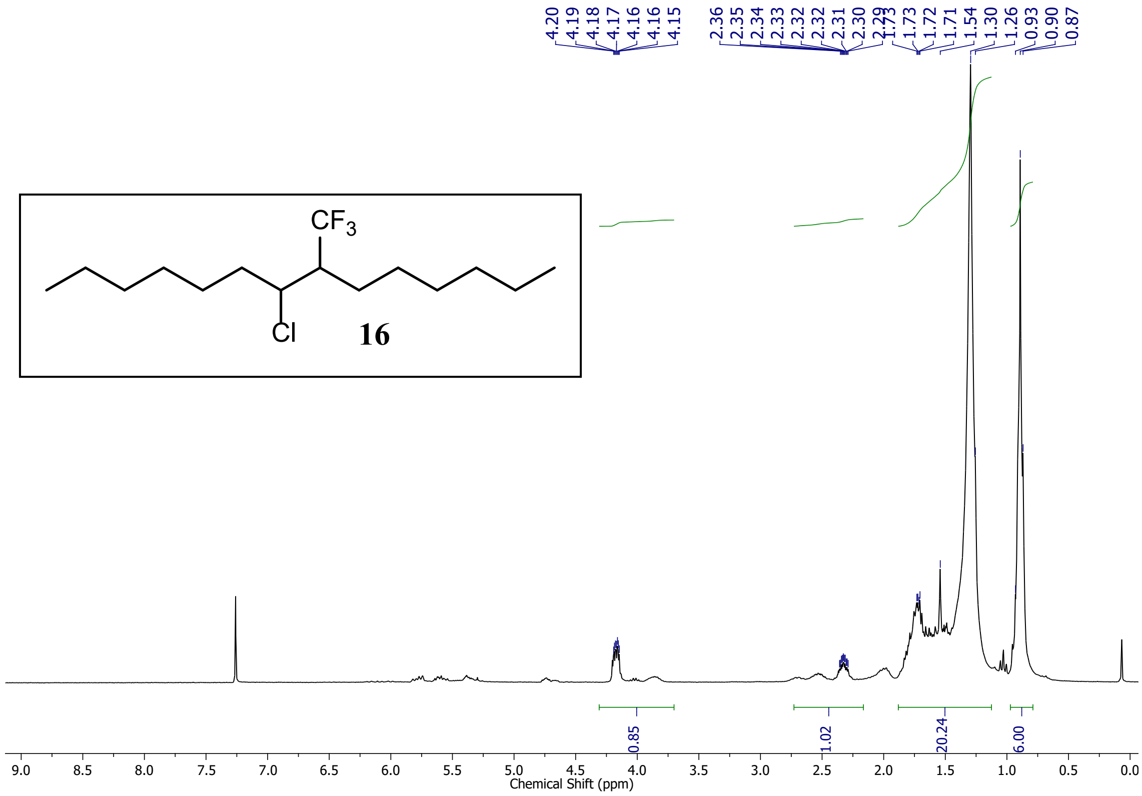

^{13}C–NMR **16**

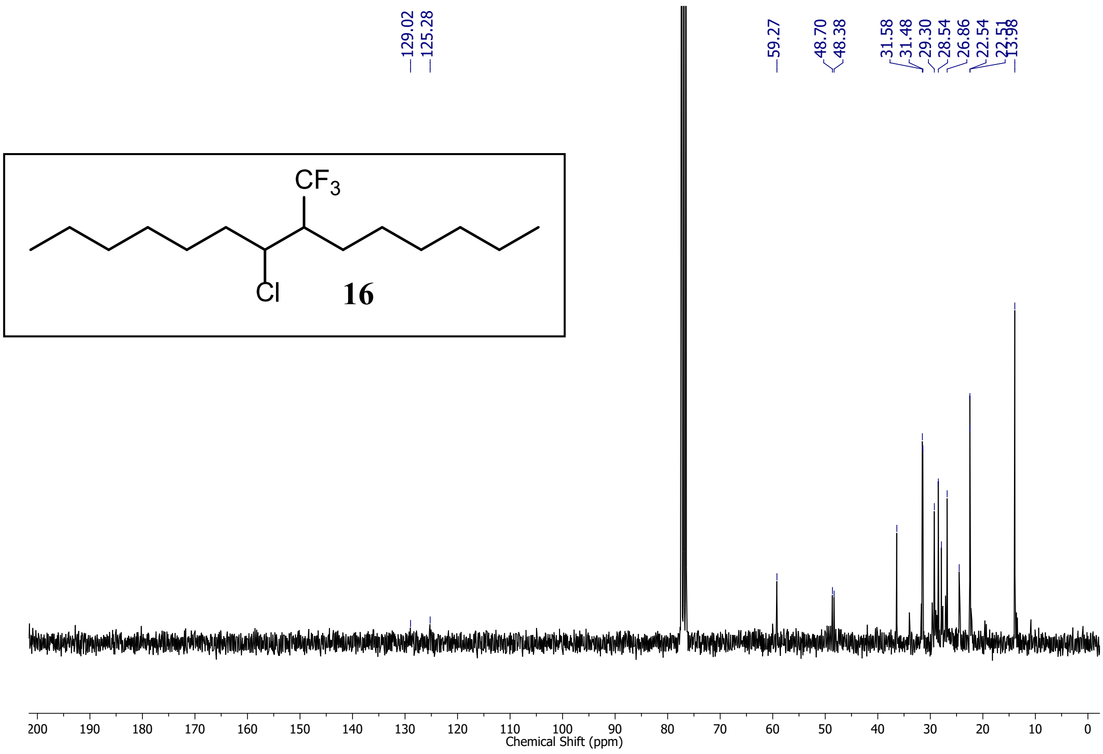

^{19}F–NMR **16**

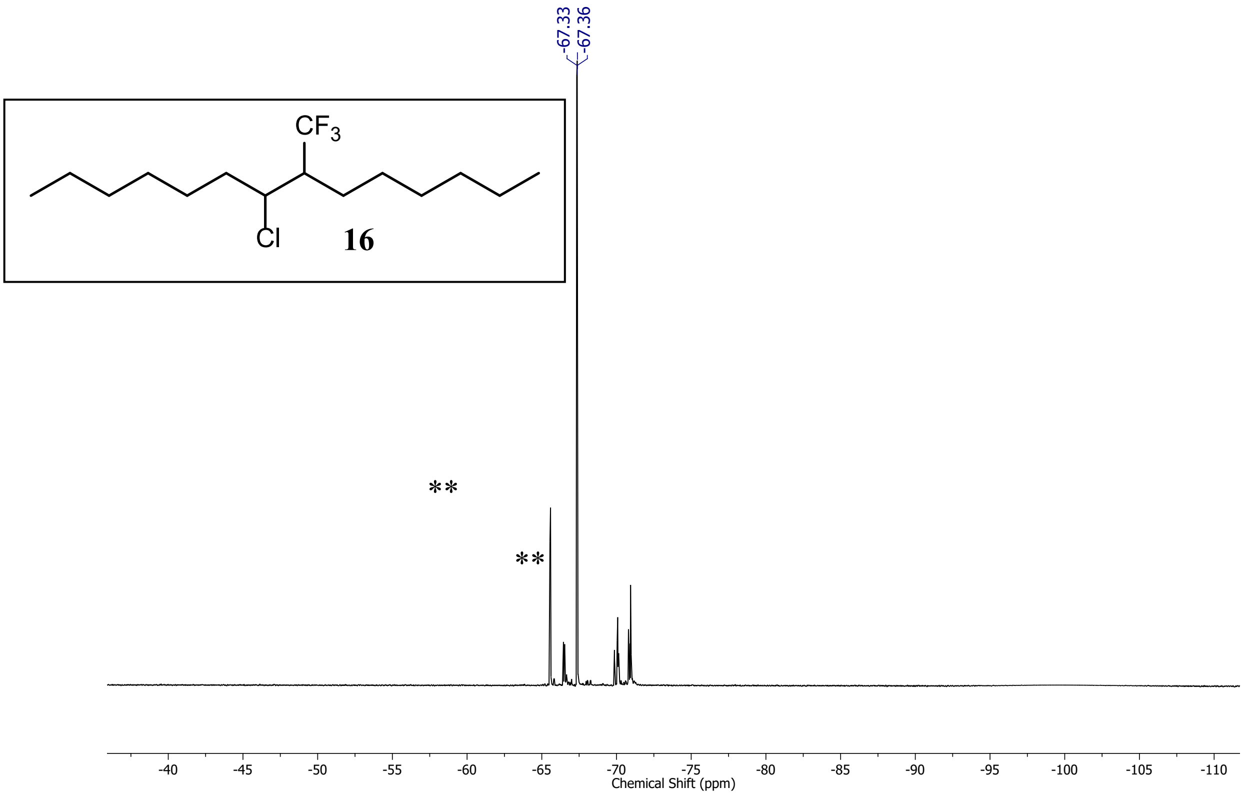

DEPT **16** (CH_2s downside)

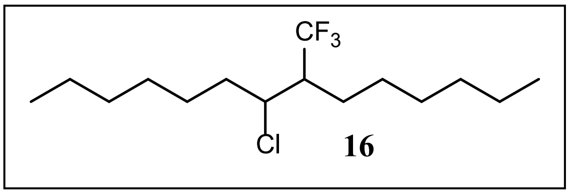

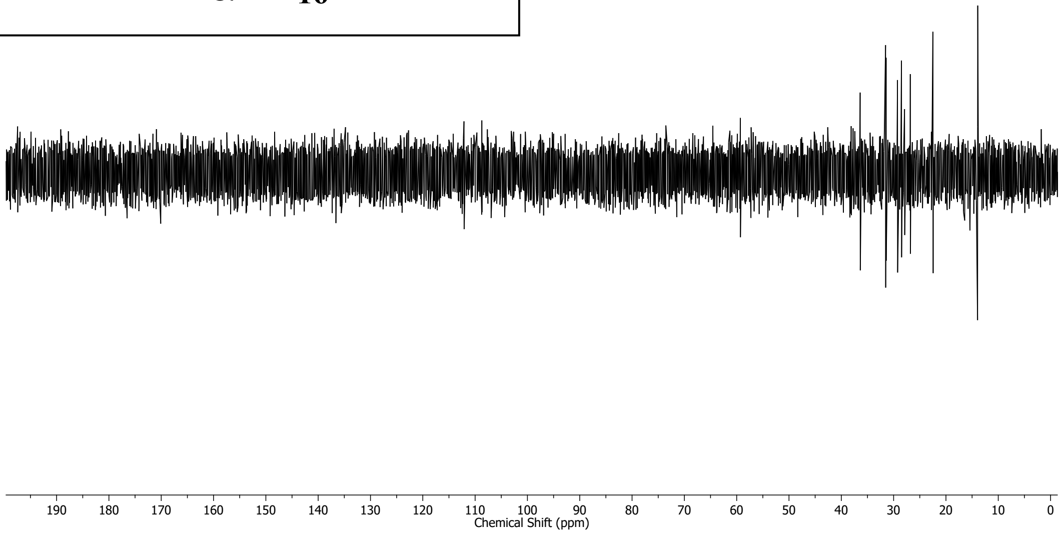

^{1}H–NMR **17**

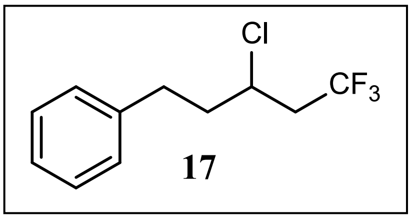

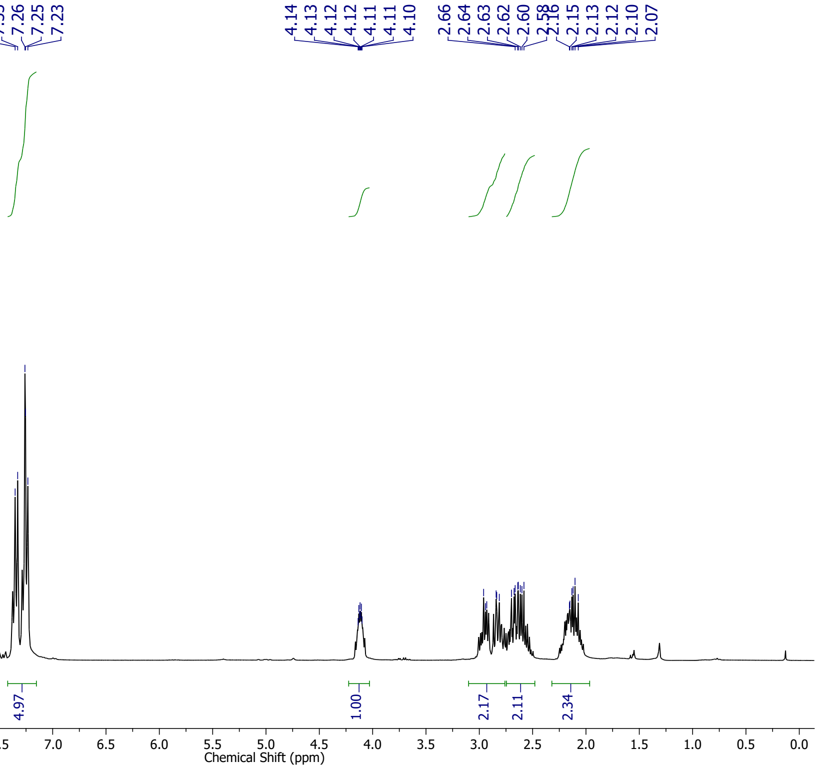

^{13}C–NMR **17**

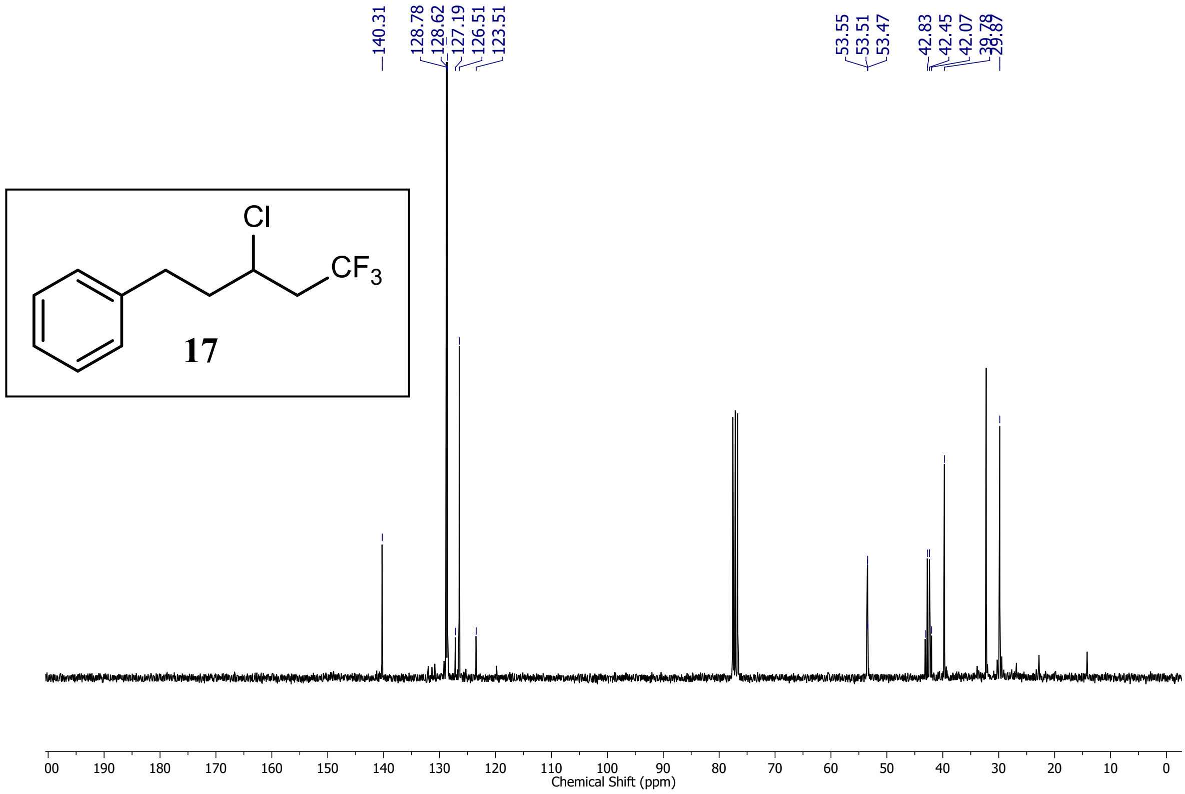

^{19}F–NMR **17**

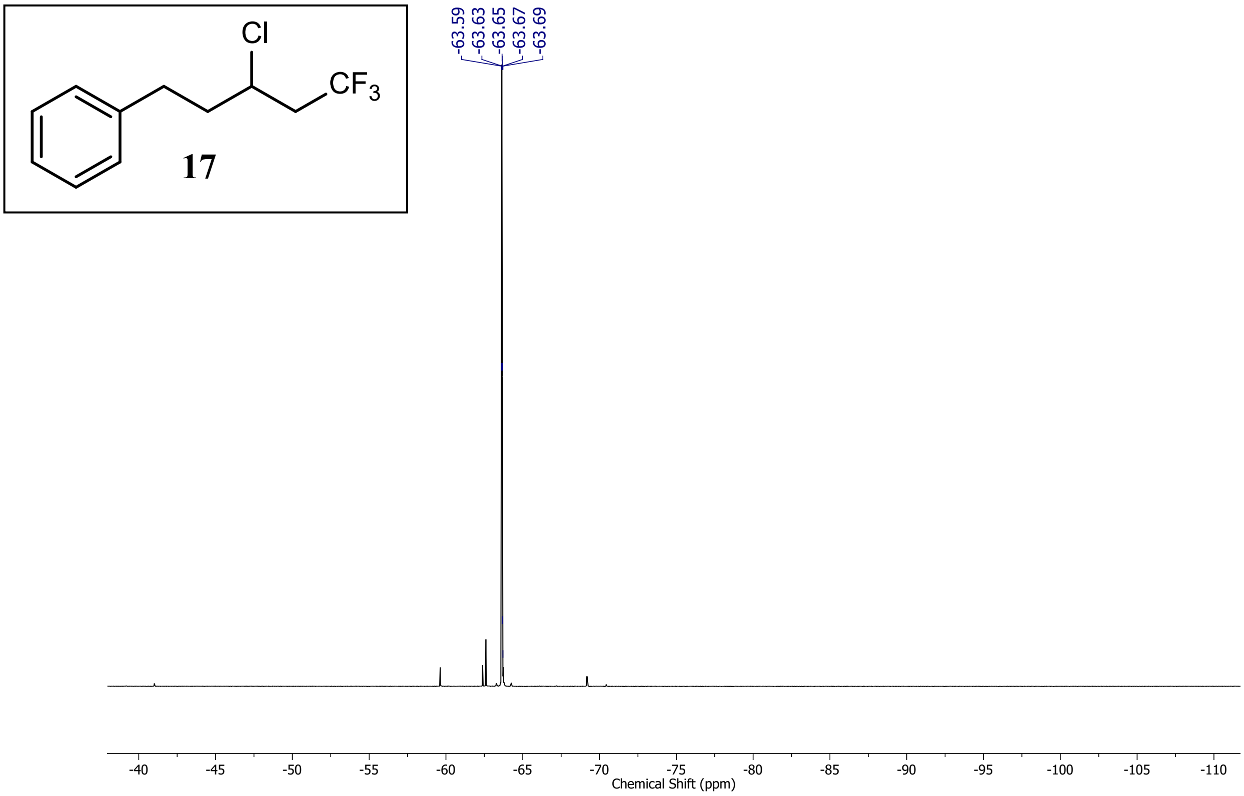

DEPT **17** (CH_2s downside)

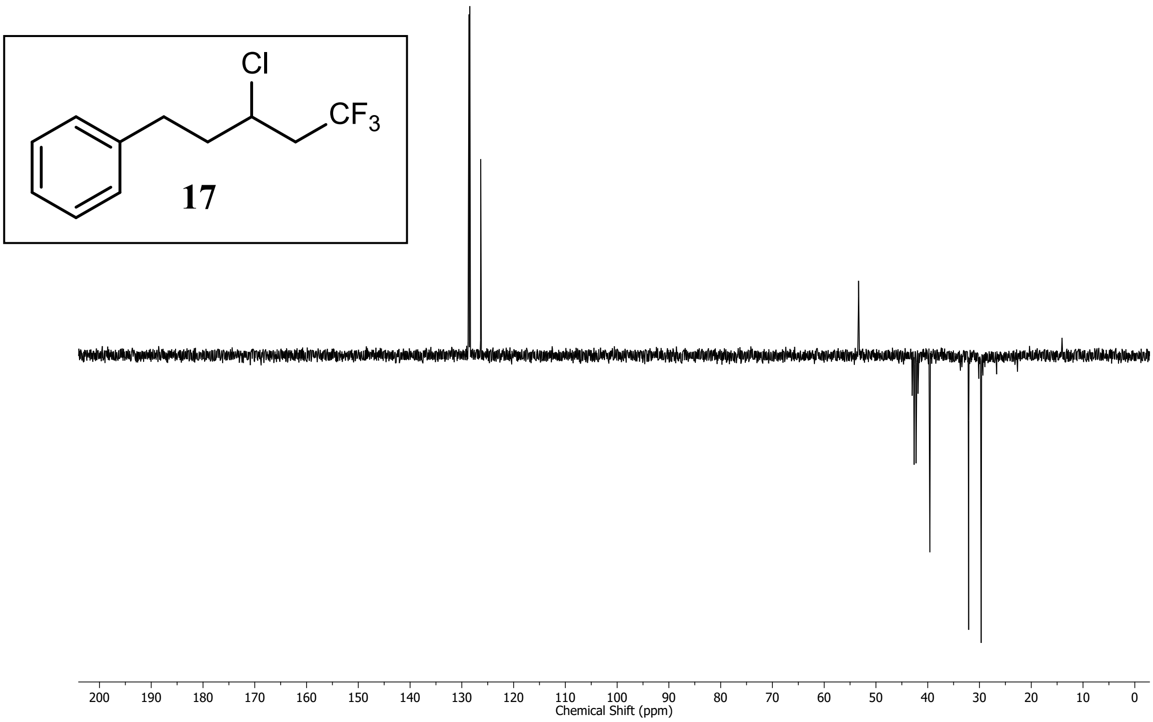

^{1}H–NMR **18**

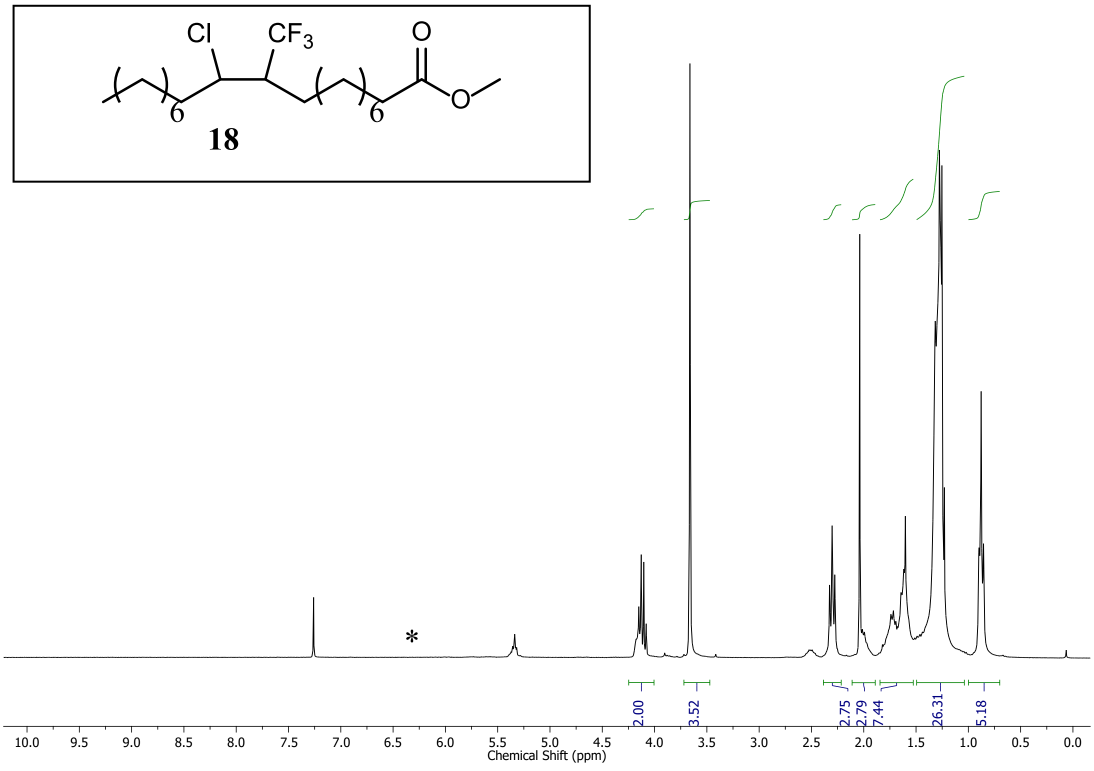

^{13}C–NMR **18**

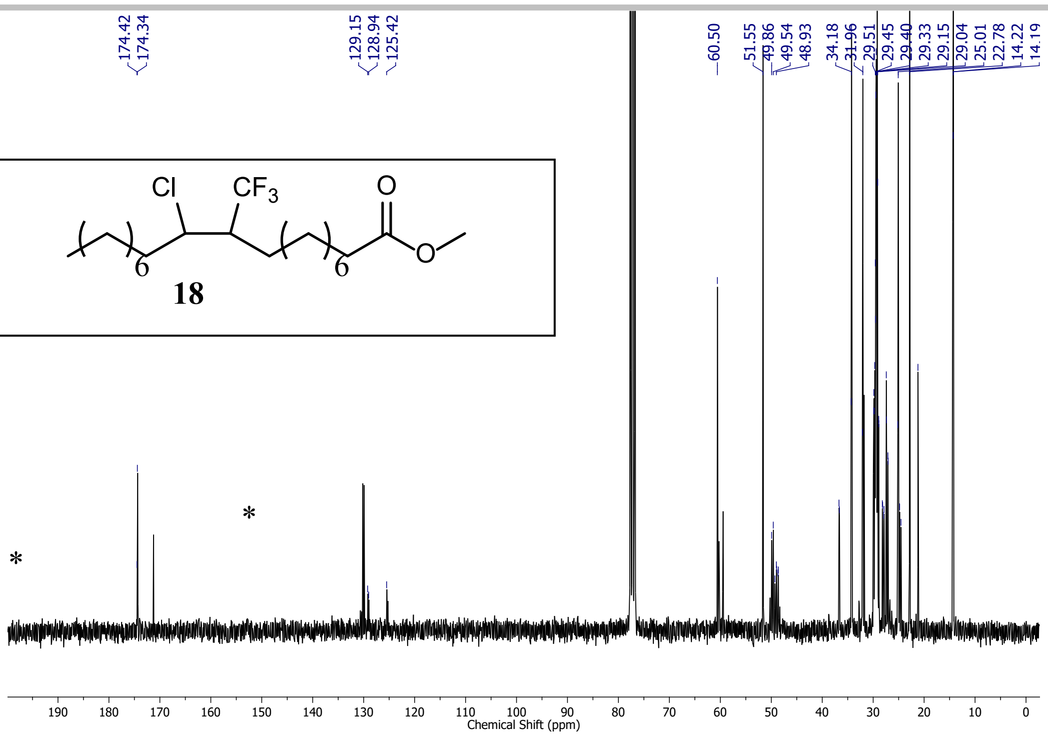

*Starting material

^{19}F–NMR **18**

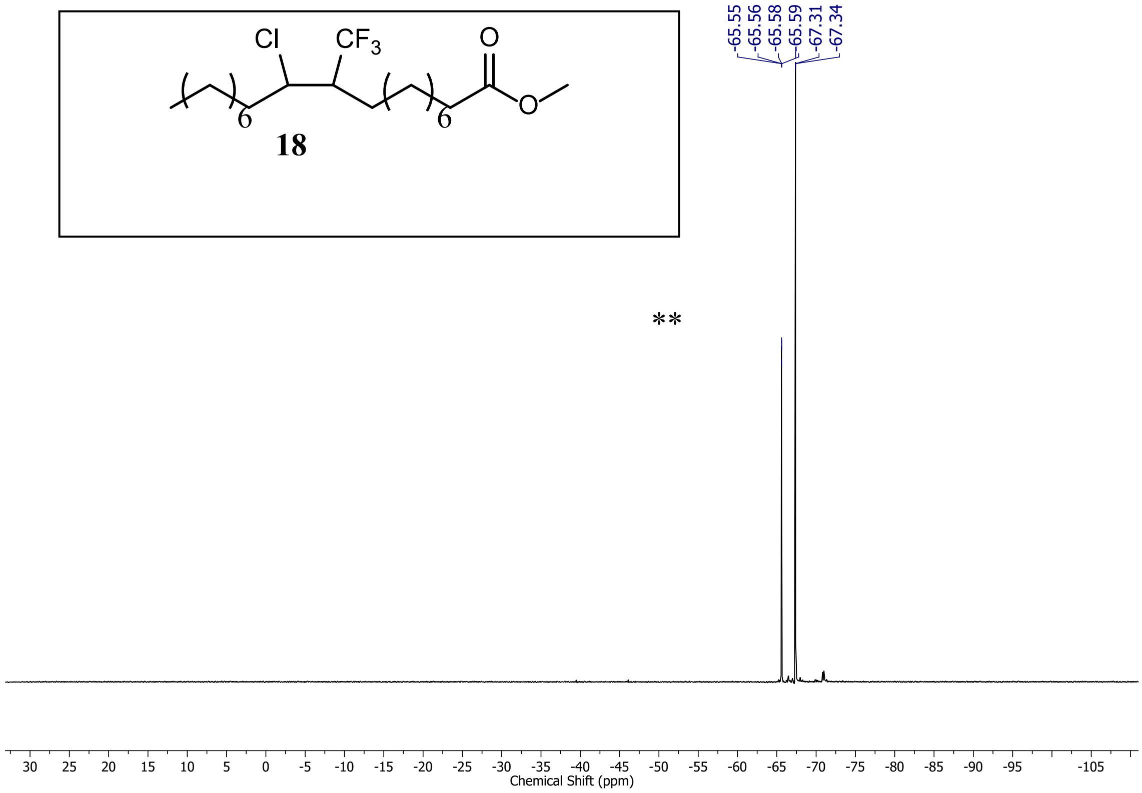

**Isomer

^{1}H–NMR **25**

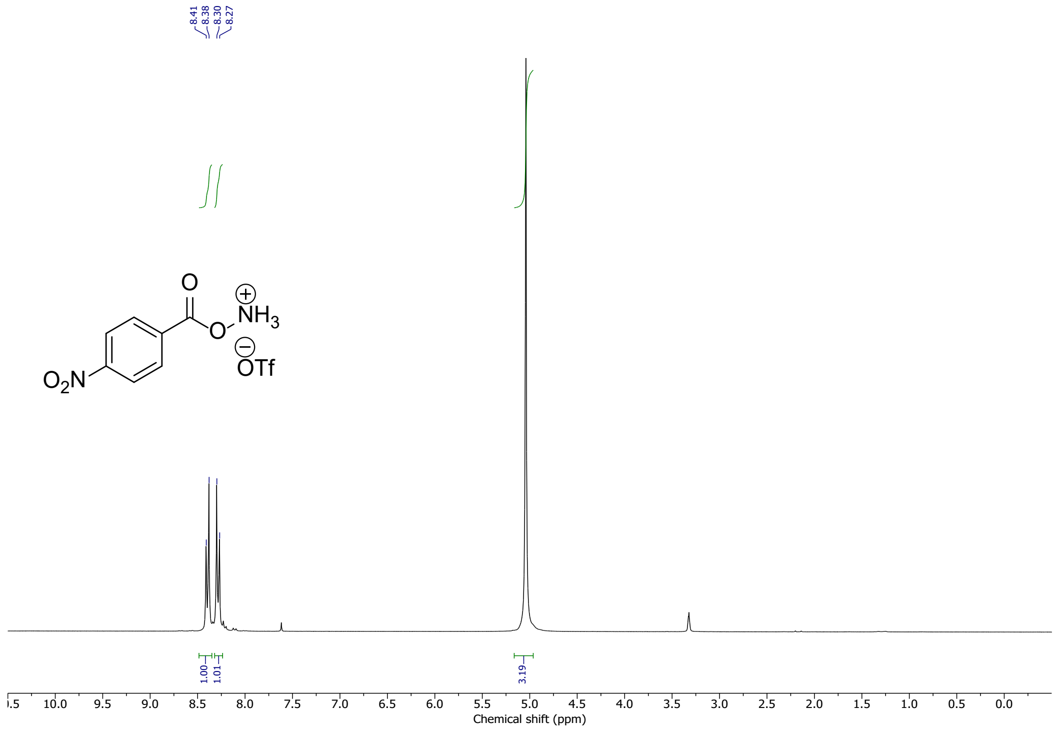

^{13}C–NMR **25**

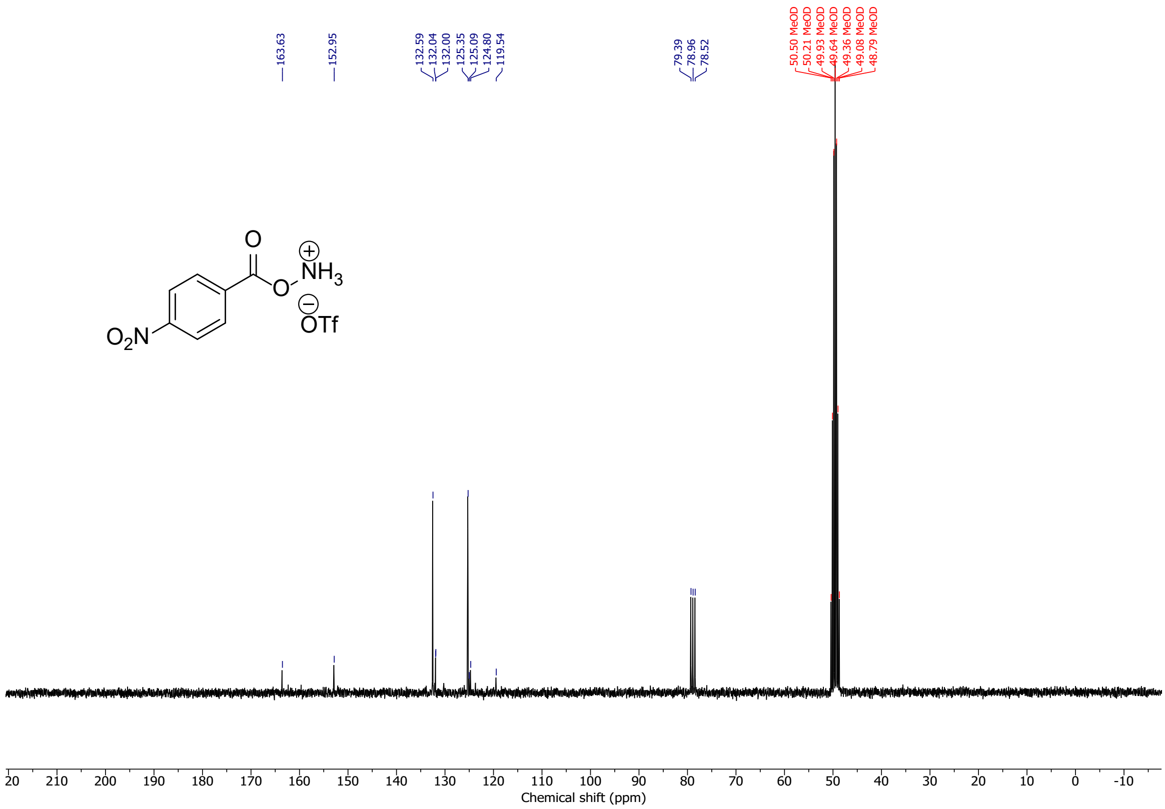

DEPT **25**

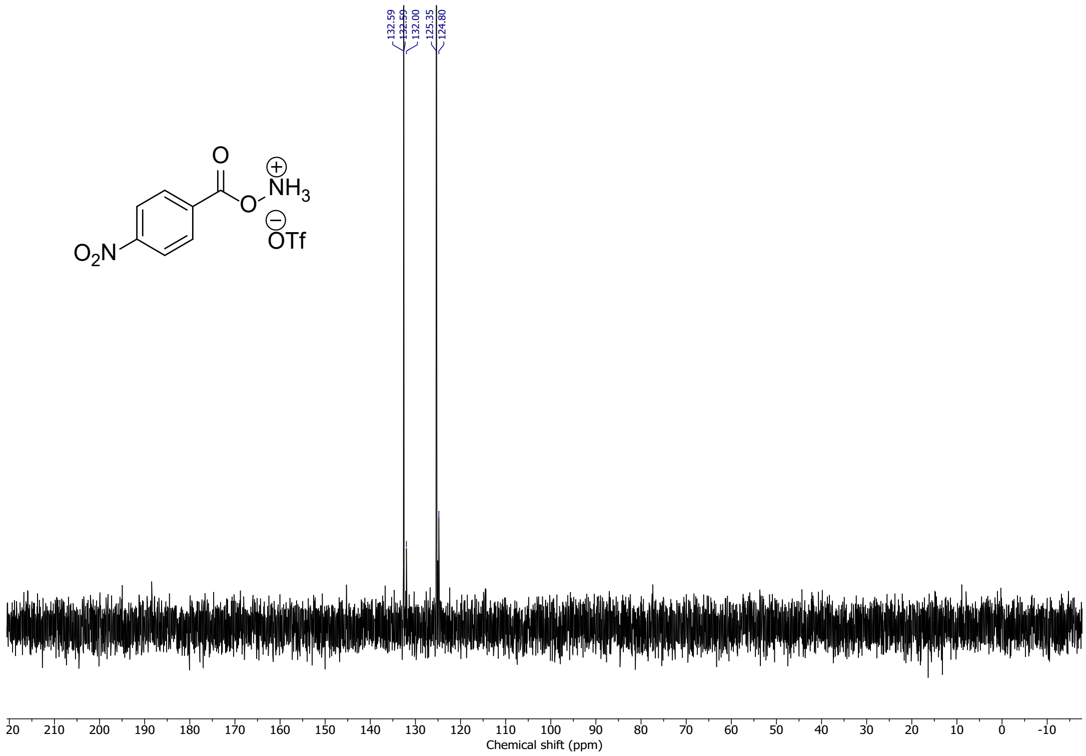

^{1}H–NMR **S1**

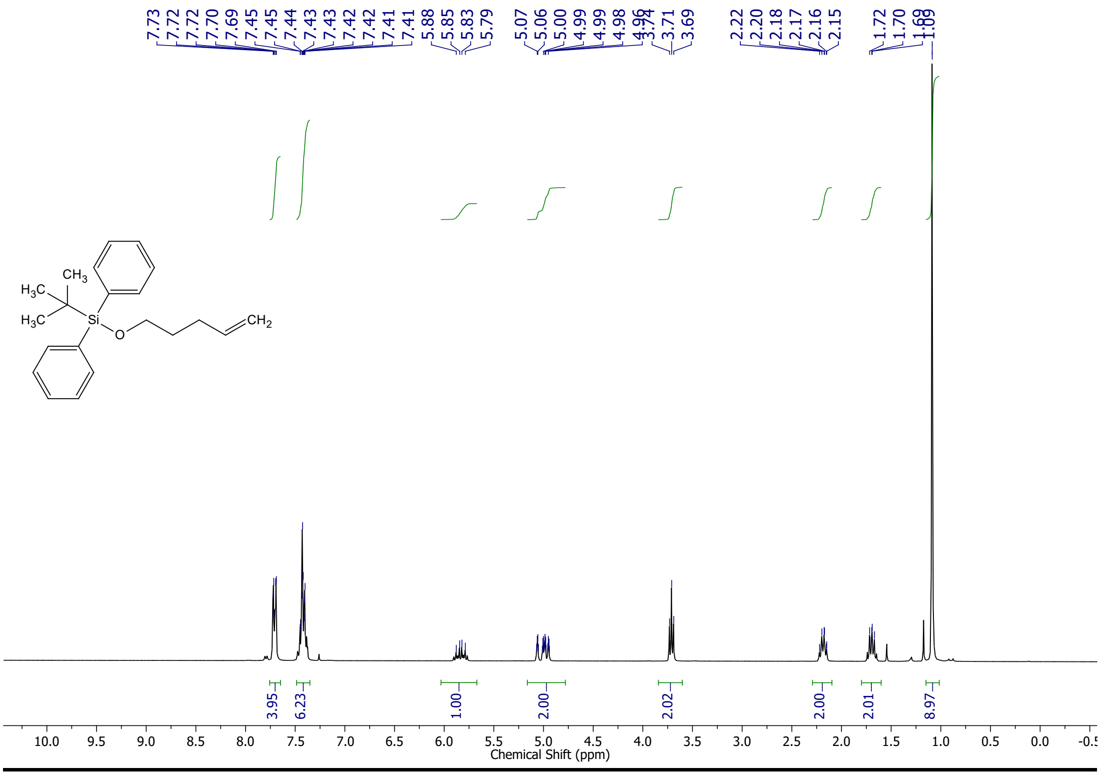

^{13}C–NMR **S1**

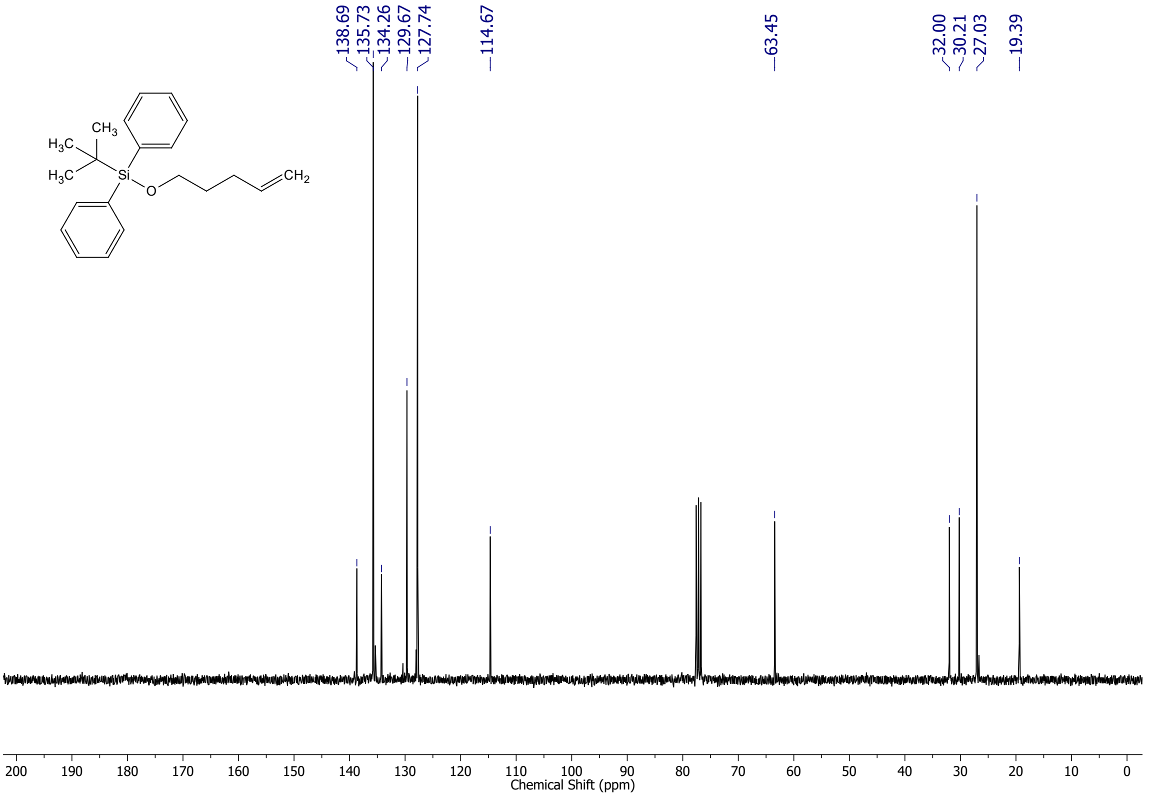

DEPT **S1** (CH_2s downside)

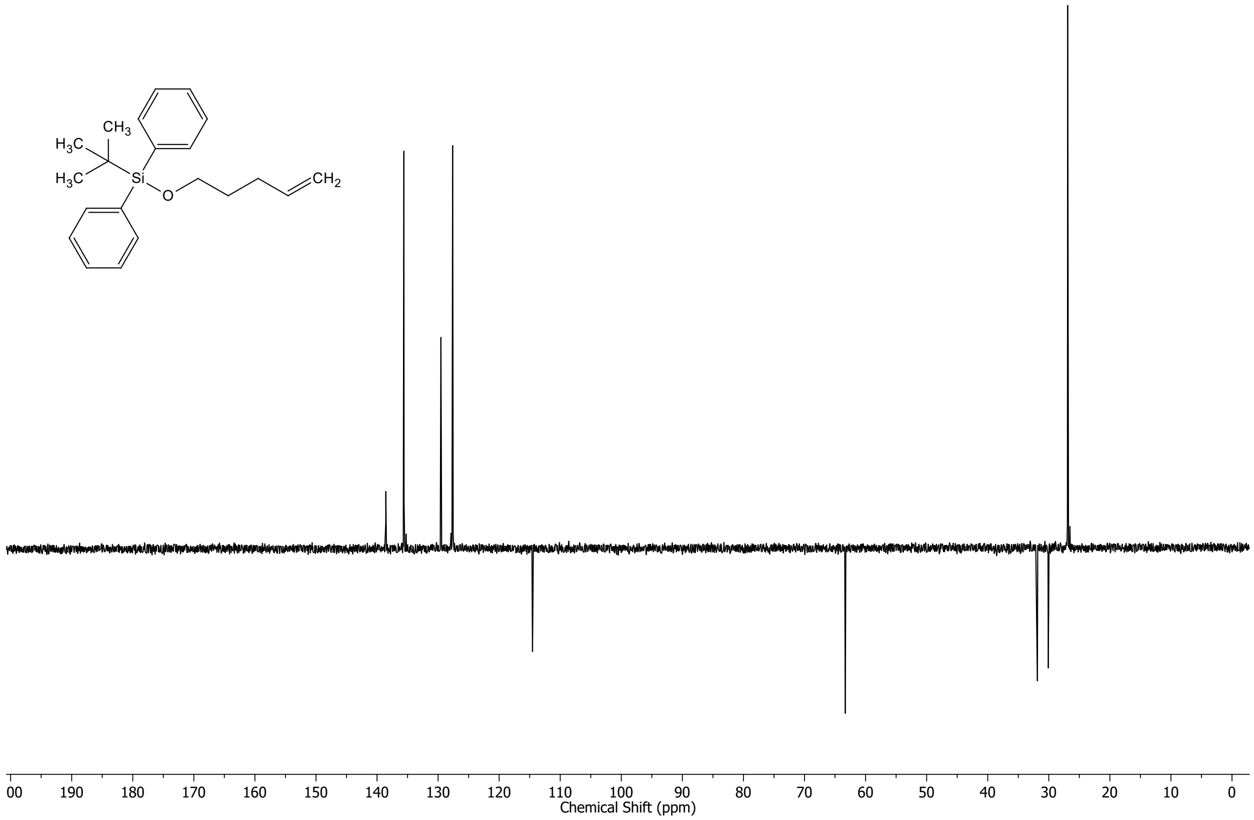

^{1}H–NMR **S2**

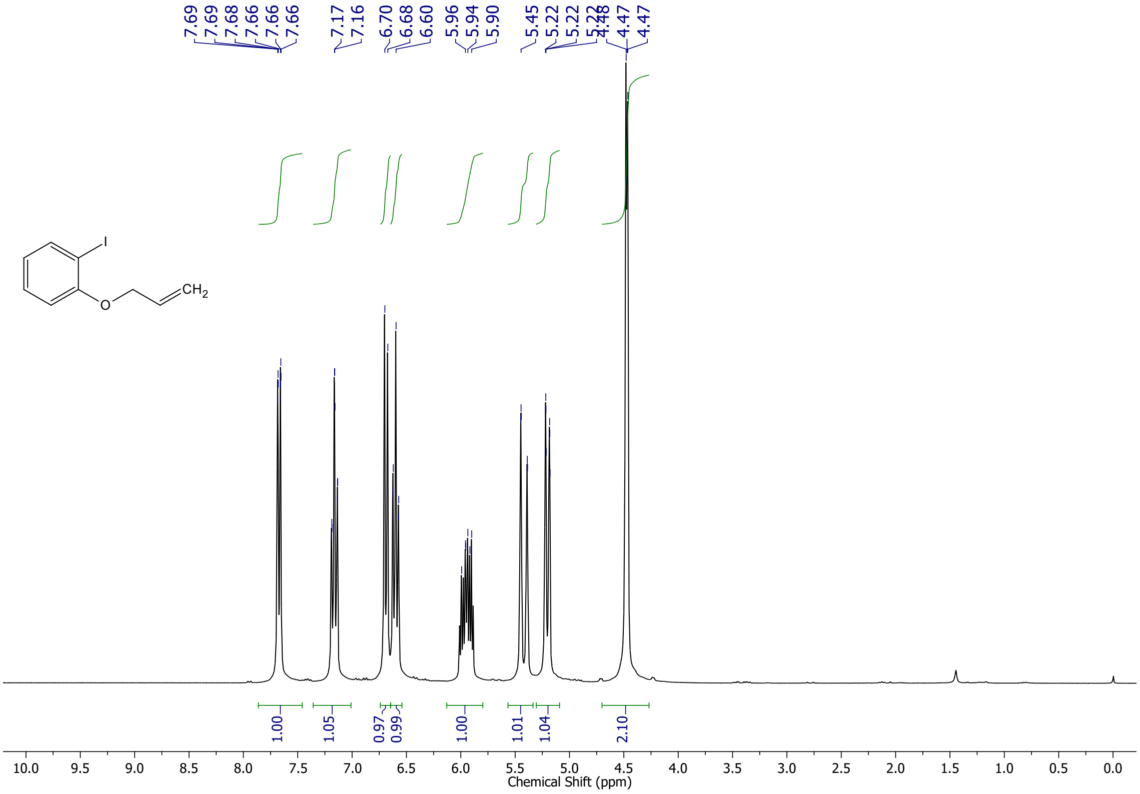

^{13}C–NMR **S2**

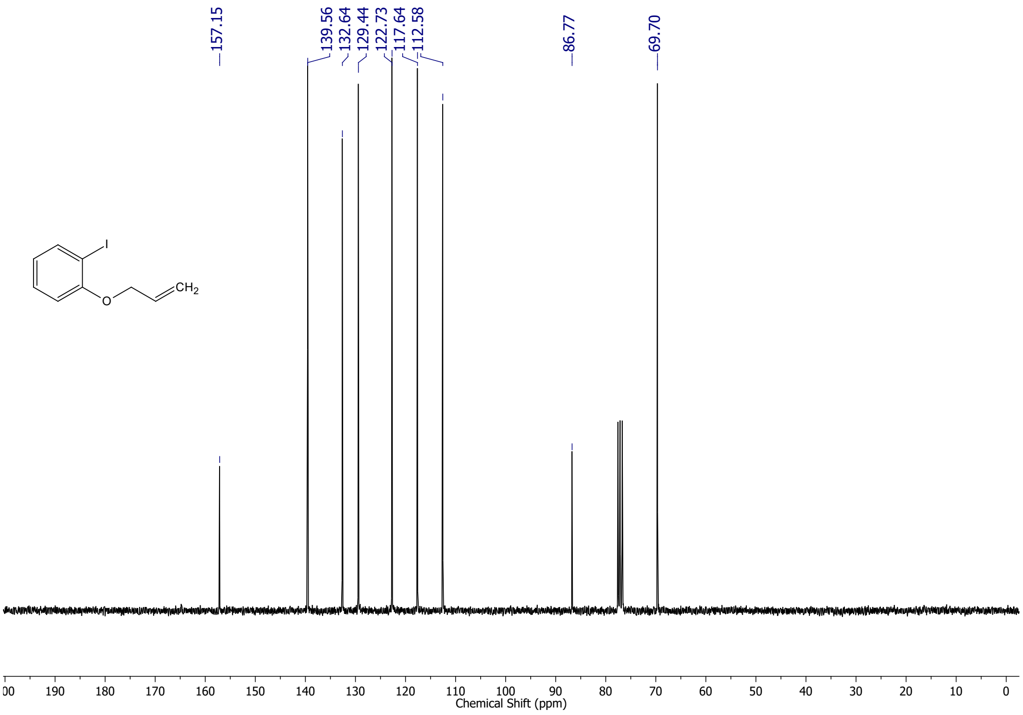

DEPT **S2** (CH_2s upside)

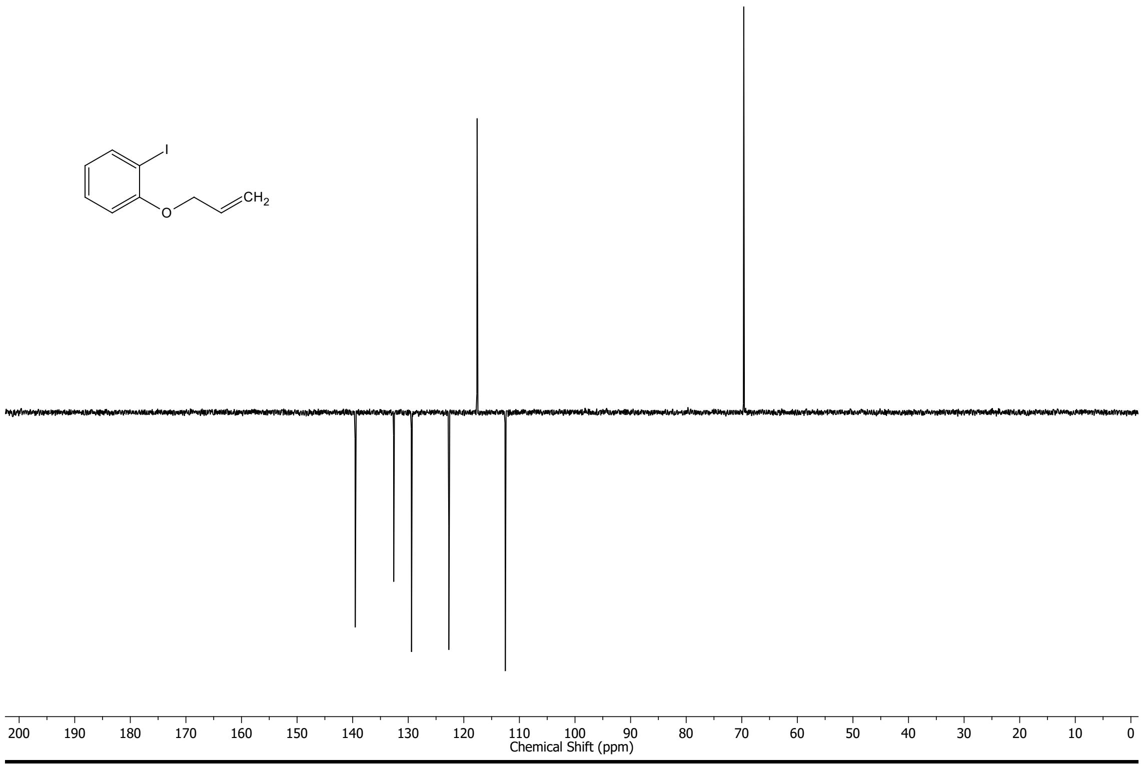

^{1}H–NMR **S3**

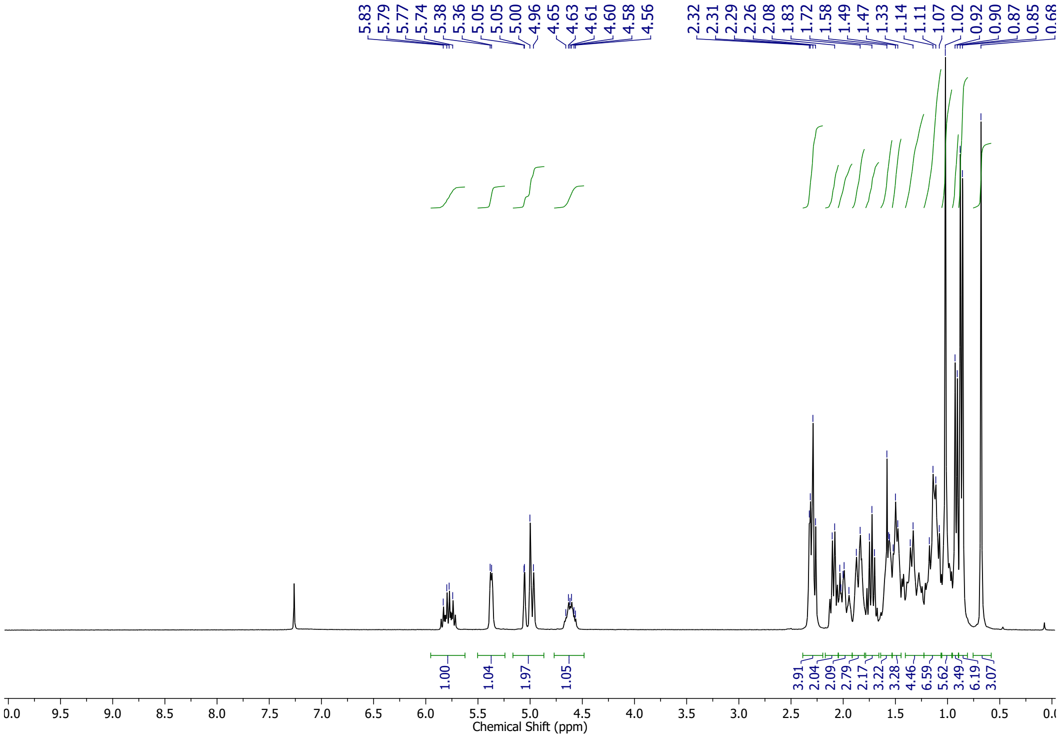

^{13}C–NMR **S3**

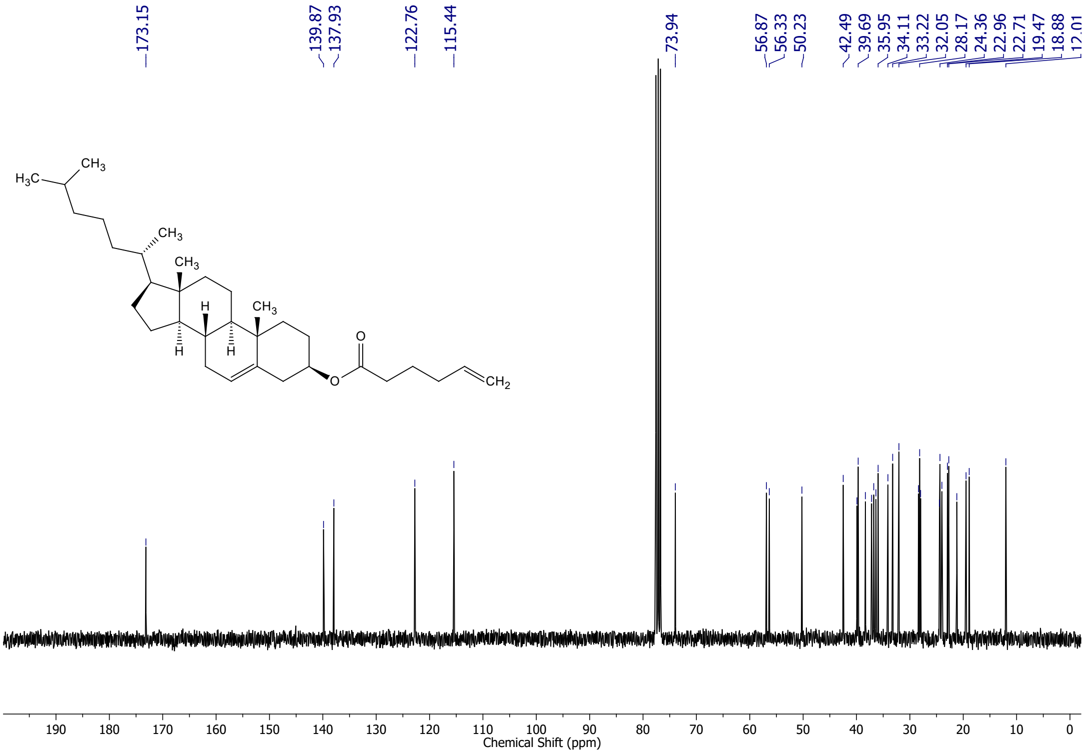

DEPT **S3** (CH_2s downside)

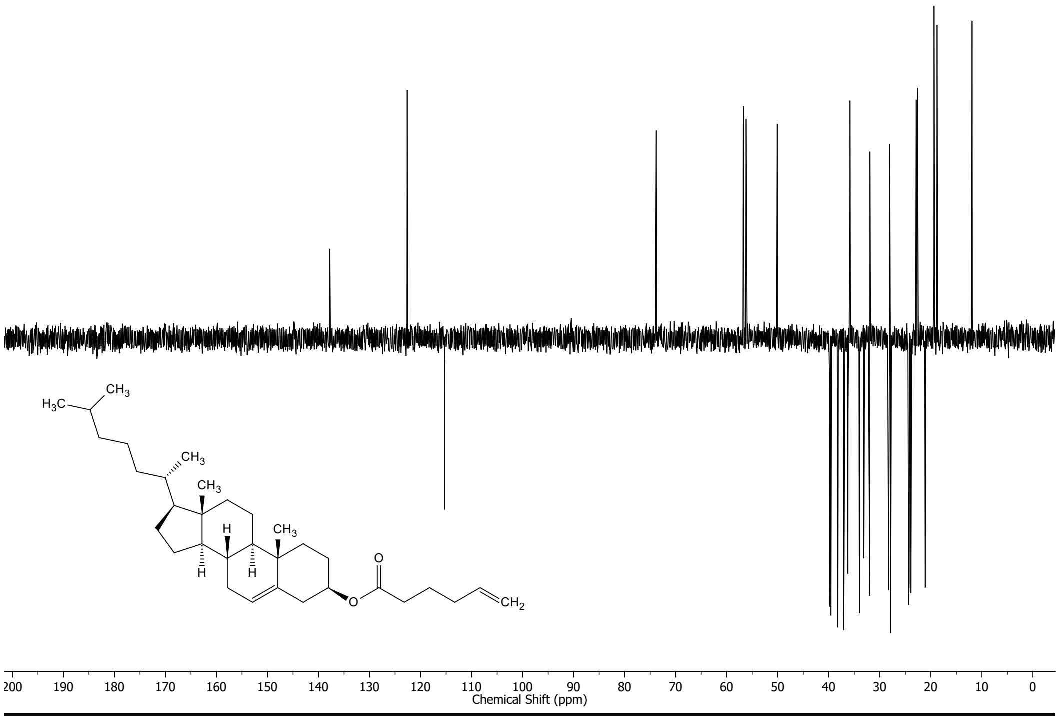

^{1}H–NMR **S4**

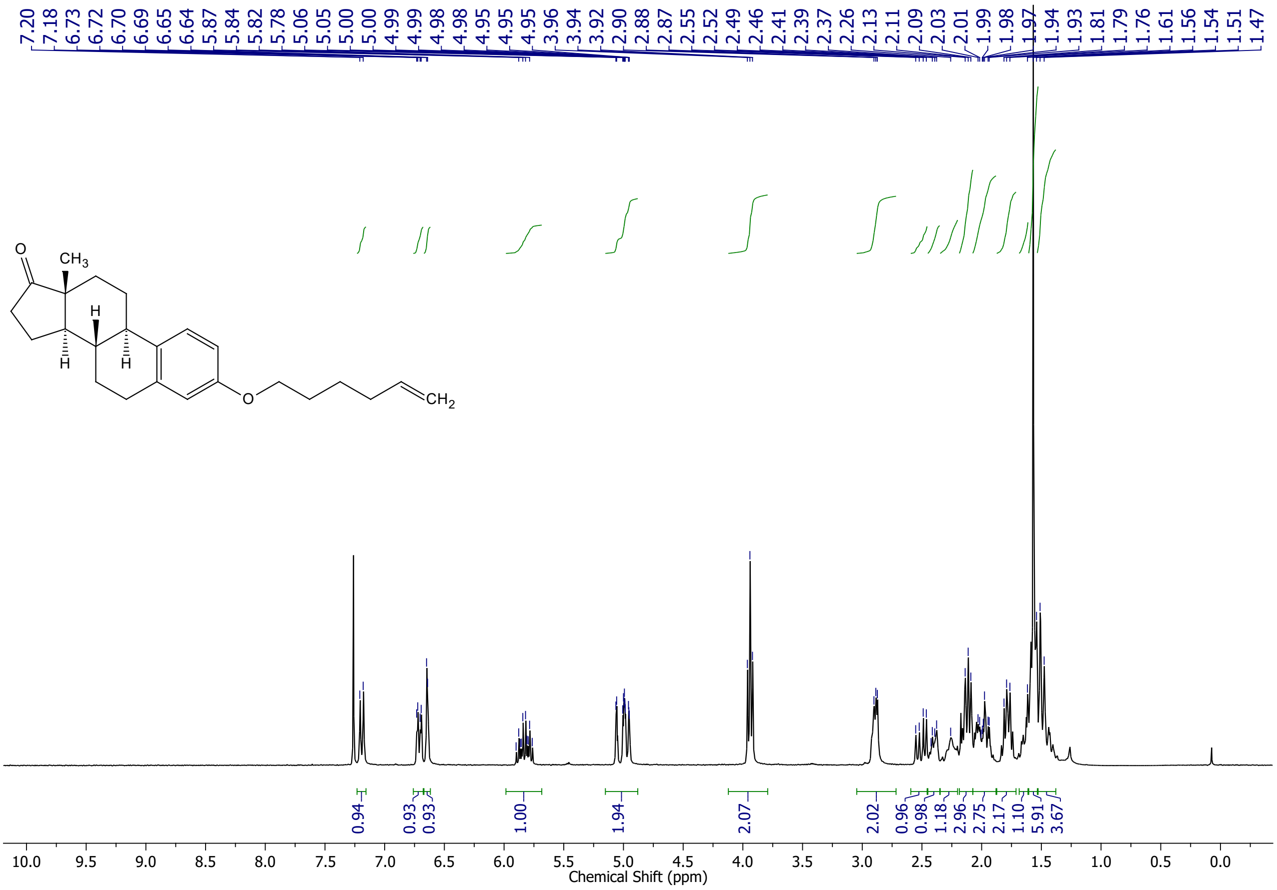

^{13}C–NMR **S4**

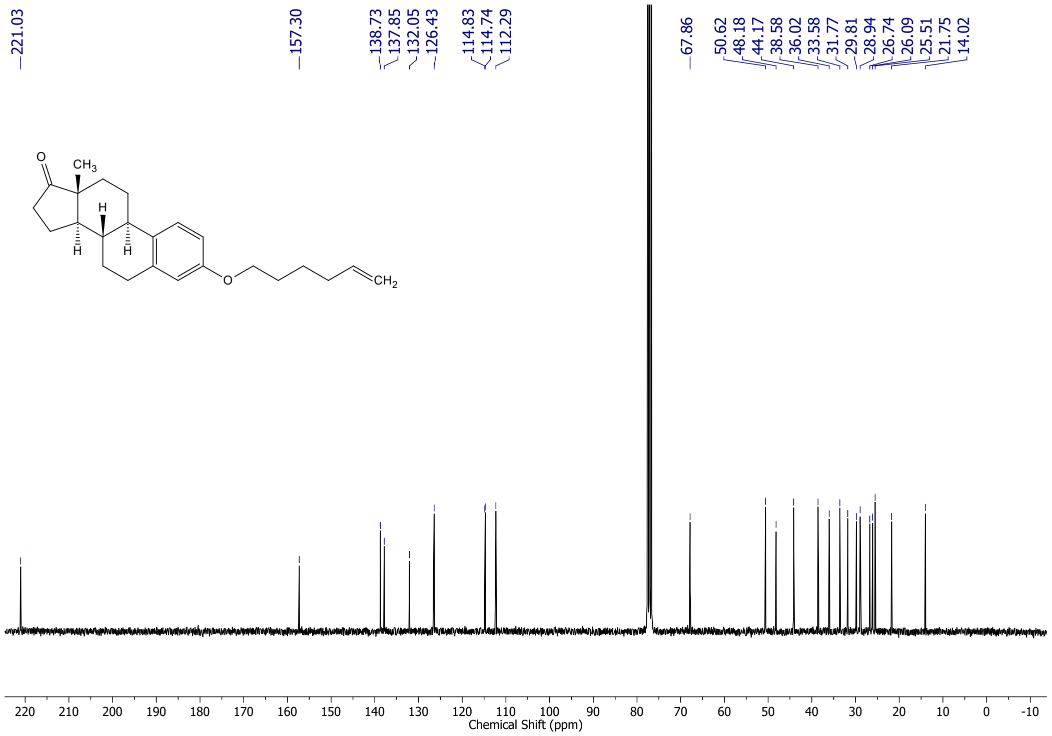

DEPT **S4** (CH_2s upside)

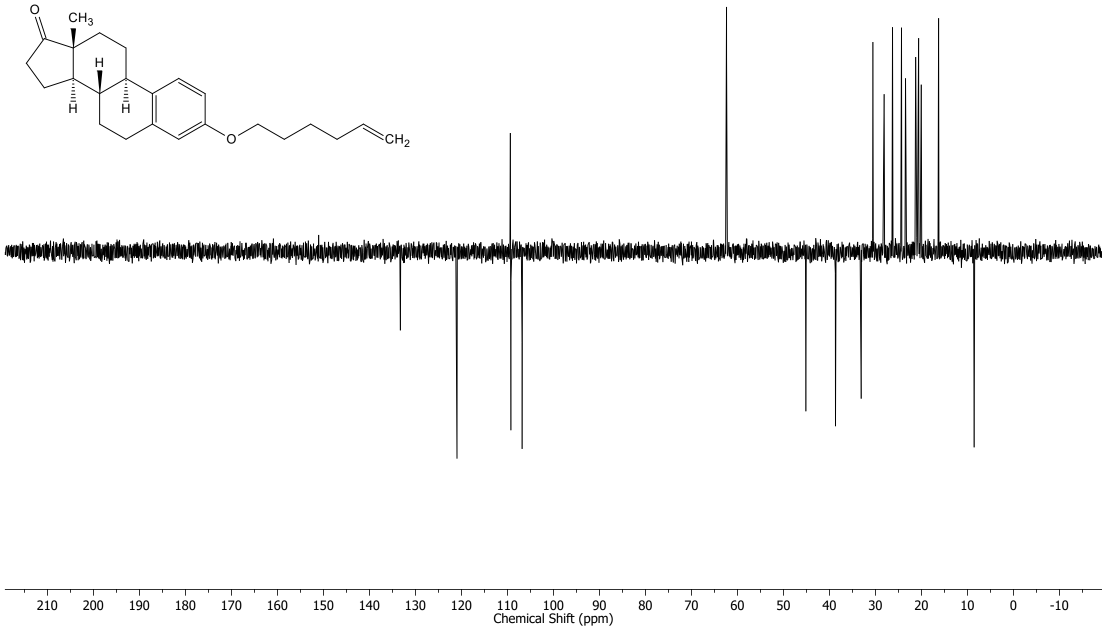

References

[1] a) G. Abellan, C. Neiss, V. Lloret, S. Wild, J. C. Chacon-Torres, K. Werbach, F. Fedi, H. Shiozawa, A. Gorling, H. Peterlik, T. Pichler, F. Hauke, A. Hirsch, *Angew. Chem. Int .Ed.* **2017**, *56*, 15267–15273; b) S. Wild, M. Fickert, A. Mitrovic, V. Lloret, C. Neiss, J. A. Vidal–Moya, M. A. Rivero–Crespo, A. Leyva–Perez, K. Werbach, H. Peterlik, M. Grabau, H. Wittkamper, C. Papp, H. P. Steinruck, T. Pichler, A. Gorling, F. Hauke, G. Abellan, A. Hirsch, *Angew. Chem. Int. Ed.* **2019**, *58*, 5763–5768.

[2] G. Abellan, M. Schirowski, K. F. Edelthalhammer, M. Fickert, K. Werbach, H. Peterlik, F. Hauke, A. Hirsch, *J. Am. Chem. Soc.* **2017**, *139*, 5175–5182.

[3] L. Legnani, G. Prina–Cerai, T. Delcaillau, S. Willems, B. Morandi, *Science* **2018**, *362*, 434–438.

[4] M. S. Kharasch, O. Reinmuth, W. H. Urry, *J. Am. Chem. Soc.* **1947**, *69*, 1105–1110.

[5] M. Asscher, D. Vofsi, *J. Chem. Soc.* **1963**, 1887–1896.

[6] J. Elzinga, H. Hogeveen, *J. Org. Chem.* **1980**, *45*, 3957–3969.

[7] F. Bellesia, L. Forti, F. Ghelfi, U. M. Pagnoni, *Synth. Commun.* **1997**, *27*, 961–971.

[8] F. de Campo, D. Lastécouères, J.–B. Verlhac, *Chem. Commun.* **1998**, 2117–2118.

[9] B. T. Lee, T. O. Schrader, B. Martin–Matute, C. R. Kauffman, P. Zhang, M. L. Snapper, *Tetrahedron* **2004**, *60*, 7391–7396.

[10] B. Boualy, M. A. Harrad, L. El Firdoussi, M. A. Ali, S. El Houssame, A. Karim, *Catal. Commun.* **2011**, *12*, 1295–1297.

[11] B. Chen, C. Fang, P. Liu, J. M. Ready, *Angew. Chem. Int. Ed.* **2017**, *56*, 8780–8784.

[12] a) C. P. Andrieux, A. Legorande, J. M. Saveant, *J. Am. Chem. Soc.* **1992**, *114*, 6892–6904; b) A. A. Isse, A. De Giusti, A. Gennaro, L. Falciola, P. R. Mussini, *Electrochim. Acta* **2006**, *51*, 4956–4964.

[13] M. Mastragostino, Casalbor.G, S. Valcher, *J. Electroanal. Chem.* **1973**, *44*, 37–45.

[14] D. T. Sawyer, G. Chlericato, C. T. Angelis, E. J. Nanni, T. Tsuchiya, *Anal. Chem.* **1982**, *54*, 1720–1724.

[15] a) F. Scholz, M. Hermes, *Electrochem. Commun.* **2000**, *2*, 814–814; b) L. Wang, Z. Sofer, M. Pumera, *ChemElectroChem.* **2015**, *2*, 324–327.

[16] M. C. Watts, L. Picco, F. S. Russell–Pavier, P. L. Cullen, T. S. Miller, S. P. Bartus, O. D. Payton, N. T. Skipper, V. Tileli, C. A. Howard, *Nature* **2019**, *568*, 216–220.

[17] a) J. Oliver–Meseguer, J. R. Cabrero–Antonino, I. Dominguez, A. Leyva–Perez, A. Corma, *Science* **2012**, *338*, 1452–1455; b) A. Leyva–Perez, J. Oliver–Meseguer, P. Rubio–Marques, A. Corma, *Angew. Chem. Int. Ed.* **2013**, *52*, 11554–11559; c) J. Oliver–Meseguer, L. C. Liu, S. Garcia–Garcia, C. Canos–Gimenez, I. Dominguez, R. Gavara, A. Domenech–Carbo, P. Concepcion, A. Leyva–Perez, A. Corma, *J. Am. Chem. Soc.* **2015**, *137*, 8310–8310.

[18] a) J. P. Collman, *Acc. Chem. Res.* **1975**, *8*, 342–347; b) M. S. Holzwarth, I. Alt, B. Plietker, *Angew. Chem. Int. Ed.* **2012**, *51*, 5351–5354; c) D. S. Carter, D. L. Van Vranken, *Org. Lett.* **2000**, *2*, 1303–1305.

[19] T. M. Bockman, H. C. Cho, J. K. Kochi, *Organometallics* **1995**, *14*, 5221–5231.

[20] A. Klein, Y. von Mering, A. Uthe, K. Butsch, D. Schaniel, N. Mockus, T. Woike, *Polyhedron* **2010**, *29*, 2553–2559.

Author Contributions

M. T.–S. and V. Ll. synthesized the catalysts, and performed and interpreted the catalytic and characterization experiments. B. G. M. and F. S. performed, interpreted and wrote the EPR part, and also revised the manuscript. F. H. performed the synthesis of FL–BP and A. H. supervised the synthetic and catalytic part. A. D.–C. performed, interpreted, supervised and wrote the electrochemistry part. G. A. supervised the FL–BP part work and wrote the manuscript. A. L.–P. performed and interpreted some catalytic experiments, supervised the catalytic part and wrote the manuscript.
